# RalGAP complexes control secretion and primary cilia in pancreatic disease

**DOI:** 10.26508/lsa.202403123

**Published:** 2025-06-09

**Authors:** Lisa H Apken, Hannah Barz, Stephanie Beel, Esther Michalke, René Rasche, Archana Verma, Andrea Ricker, Harald Nüsse, Jürgen Klingauf, Kornelius Kerl, Eva Wardelmann, Daniel Kümmel, Konrad Steinestel, Zoltán Pethő, Michael Meisterernst, Andrea Oeckinghaus

**Affiliations:** 1 Institute of Molecular Tumor Biology, Faculty of Medicine, University Münster , Münster, Germany; 2 Institute of Biochemistry, University Münster , Münster, Germany; 3 Department of Pediatric Hematology and Oncology, University Children’s Hospital Münster, Münster, Germany; 4 Institute of Medical Physics and Biophysics, University Münster , Münster, Germany; 5 Gerhard-Domagk-Institute of Pathology, Faculty of Medicine, University Münster , Münster, Germany; 6 Institute of Pathology and Molecular Pathology, Bundeswehrkrankenhaus Ulm, Ulm, Germany; 7 Institute of Physiology II, University Münster , Münster, Germany; 8 Department of Metabolism, Senescence and Autophagy, Research Center One Health Ruhr, University Alliance Ruhr and University Hospital Essen, University Duisburg–Essen, Essen, Germany

## Abstract

κB-Ras/RalGAP complexes limit the activity of Ral GTPases, which function in EGFR/Ras signaling. RalGAP expression is down-regulated in pancreatic cancer; however, the role of RalGAP and Ral GTPases in tumor development in vivo remained unclear. Here, we show that pancreatic RalGAPβ deficiency alone is sufficient to induce inflammation and neoplasia in vivo. We identify that this phenotype is triggered by disturbance of the secretory pathway and polarized exocytosis in acinar cells, demonstrating that RalGAP complexes uphold spatial control of Ral activity. We furthermore show that RALGAPβ deficiency results in defective primary cilium assembly, a process required for efficient acinar regeneration upon inflammation. Only primary cilium formation depends on κB-Ras proteins, suggesting that κB-Ras proteins are not essential for all RalGAP complex-controlled processes. In combination with an oncogenic *KRAS*^G12D^ mutation, RalGAPβ deficiency leads to a dramatic shortening of tumor latency and median survival. Our results highlight an important role of RalGAP/Ral signaling in upholding acinar cell identity and preventing pancreatic cancer development.

## Abstract (precis)

Loss of RalGAP impacts secretion, cell polarity, and primary cilium formation, results in pancreatitis and neoplasia development in the pancreas, and promotes mutant KRas-driven tumorigenesis.

## Introduction

The pancreas is a glandular organ with endocrine and exocrine functions (Atkinson et al, 2020). Acinar cells, polarized epithelial cells of the exocrine pancreas, store digestive enzymes such as amylase or trypsinogen in apically located zymogen granules and secrete them through compound exocytosis via the apical membrane into the acinar lumen (Pickett & Edwardson, 2006). Inappropriate activation of digestive enzymes inside the cells represents an early event in acute pancreatitis (Murtaugh & Keefe, 2015; Gorelick et al, 2018; Mayerle et al, 2019). Increased exocytosis (Voronina et al, 2022), endoplasmic reticulum (ER) stress (Logsdon & Ji, 2009; Borrello et al, 2022), and blockage of apical secretion with redirection of zymogen granules to basolateral membranes (Dolai et al, 2018, 2021) and subsequent release of zymogen granules into the interstitium are discussed as early pathophysiological events. Adult pancreatic acinar cells can transform transcriptionally and morphologically into duct-like cells in a process termed acinar-to-ductal metaplasia (ADM) upon inflammation or injury. Centroacinar cells, which reside at the junction of acinar cells and the adjacent ductal epithelium, have been reported as an alternative source for ADM (Stanger et al, 2005; Furuyama et al, 2011; Westphalen et al, 2016). ADM is generally reversible and facilitates organ regeneration (Storz, 2017; Marstrand-Daucé et al, 2023). However, upon chronic inflammation and injury or sustained oncogenic activation of the small GTPase KRas, ADM can become persistent and result in the formation of pancreatic neoplasia and cancer (Guerra et al, 2007, 2011; Ji et al, 2009; Kong et al, 2018).

Pancreatic cancer is highly lethal with a 5-yr survival rate of only 12% (Siegel et al, 2023) with pancreatic ductal adenocarcinoma (PDAC) representing the most common type (Ryan et al, 2014). PDAC cells show a ductal phenotype, but acinar-to-ductal metaplasia has been reported to precede development of precursor PanIN lesions (pancreatic intraepithelial neoplasia) and PDAC (Storz, 2017; Marstrand-Daucé et al, 2023). Lineage-tracing studies in mouse models suggest that acinar, centroacinar, and ductal cells can undergo malignant transformation, with models affecting acinar and centroacinar cells being most efficient in PanIN formation (Grimont et al, 2021). Mutations in *KRAS* occur early (stage 1 PanINs) and are found in very high frequency in PDAC (∼85–95%) (Cox et al, 2014), highlighting KRas signaling as a key oncogenic driver pathway (Luo, 2021). The expression of mutated KRas in mice recapitulates the histology of human disease, including PanIN development, but rarely results in invasive carcinoma development without additional genetic alterations (Hingorani et al, 2003).

KRas belongs to the family of small GTPases, hydrolytic enzymes that convert GTP into GDP (Goitre et al, 2014). In their GTP-bound “ON” state, GTPases interact with effector proteins to convey downstream signaling. GTPase-activating proteins (GAPs) are required to promote efficient GTP hydrolysis and thus represent negative regulators of GTPase-driven signaling (Cherfils & Zeghouf, 2013). The GDP-bound “OFF” state is reversed by guanine nucleotide exchange factors (GEFs), which facilitate the replacement of GDP by GTP (Cherfils & Zeghouf, 2013). The oncogenic hotspot mutations G12, G13, and Q61 lock KRas in its GTP-bound “ON” state that is able to engage downstream pathways such as PI3K/Akt, Raf/MAPK, and Ral GTPase signaling, which can contribute to tumorigenesis (Eser et al, 2014). In pancreatic cancer cell lines and patient tissue, Ral GTPases are more consistently hyperactivated than other Ras effectors (Lim et al, 2005, 2006; Jones et al, 2008).

RalGAP1 and RalGAP2, the GTPase-activating protein complexes controlling activity of the Ral GTPases RalA and RalB, are large heterodimeric complexes consisting of a common regulatory RalGAPβ (p170, RGC1) subunit and one of the two catalytic α-subunits RalGAPα1 (p240, GARNL1) or RalGAPα2 (p220, AS250) (Gridley et al, 2005, 2006; Shirakawa et al, 2009; Chen et al, 2011). The association of RalGAPβ is essential for GAP activity (Shirakawa et al, 2009). In addition, binding of the small κB-Ras GTPases to the α-subunits promotes RalGAP activity in cells with the underlying molecular reason remaining elusive (Oeckinghaus et al, 2014; Beel et al, 2020). Effectors of Ral GTPases include the exocyst subunits Sec5 and Exo84 (Moskalenko et al, 2003; Spiczka & Yeaman, 2008; He & Guo, 2009) and RalBP1/RLIP76 (Ral-binding protein 1), which connect Ral to the control of vesicle trafficking and exocytosis (Guo et al, 2000; Hsu et al, 2004; Wu & Guo, 2015) or actin dynamics (Cantor et al, 1995; Jullien-Flores et al, 1995; Awasthi et al, 2008; Fenwick et al, 2010) and endocytosis (Jullien-Flores et al, 2000; Rossé et al, 2003; Han et al, 2009; Johansson et al, 2019), respectively. Knockdown studies in PDAC cell lines established nonredundant functions for the Ral isoforms, with RalA mediating anchorage-independent proliferation and RalB cell survival, invasion, and metastasis (Lim et al, 2005, 2006; Falsetti et al, 2007; Neel et al, 2012). In addition, knockout of RalGAPβ in pancreatic tumor cell lines has been shown to promote migration, invasion, and metastasis in tissue culture and xenograft models (Yoshimachi et al, 2021). However, these model systems deliver an incomplete picture as they do not address processes occurring during pancreatic tumor development in vivo.

We previously published that κB-Ras deficiency interfered with acinar regeneration and promoted mutant KRas-induced cancer development in vivo, suggesting a tumor-suppressive role of the κB-Ras:RalGAP complexes (Beel et al, 2020). However, several important issues remained unaddressed. κB-Ras proteins function in both Ral and NF-κB signaling pathways (Oeckinghaus et al, 2014), and attribution of the observed in vivo phenotype of κB-Ras–deficient mice to deregulation of Ral signaling was circumstantial. In addition, the importance of κB-Ras proteins for RalGAP activity in the pancreas remained unclear. This limited assessment of the relevance of reduced RalGAP levels observed in human patients (Cao et al, 2021). Importantly, the mechanisms underlying defective acinar regeneration and promotion of tumor development had not yet been characterized.

We now demonstrate that RalGAPβ deficiency in the pancreas results in pancreatitis and neoplasia. RalGAPβ loss impacts acinar cell polarity and leads to aberrant exocytosis of digestive enzymes, resulting in tissue damage. In addition, RalGAP is required for primary cilium formation, a process that is known to be critical for effective acinar regeneration (Cano et al, 2006; Bangs et al, 2020). In agreement, RalGAPβ-deficient mice develop persistent ADM and inflammation upon a single episode of mild acute pancreatitis. Only regulation of primary cilium formation but not control of the secretory pathway depends on the presence of κB-Ras proteins, suggesting that κB-Ras is only essential for certain RalGAP-controlled processes. When combined with oncogenic KRas^G12D^ mutant expression, RalGAPβ loss dramatically promotes PDAC development and reduces median survival of mice. Our results thus identify RalGAP/Ral signaling as a risk factor during pancreatitis and a modulator of tumor initiation in the pancreas. Because low expression levels of RalGAP and κB-Ras have been reported in human tumor samples (Oeckinghaus et al, 2014; Cao et al, 2021; Yoshimachi et al, 2021), our findings have relevance for human disease and potential therapeutic strategies in the treatment of pancreatitis and PDAC.

## Results

### Pancreatic RalGAPβ deficiency promotes ADM and leads to pancreatitis and neoplasia

**Figure S1.**
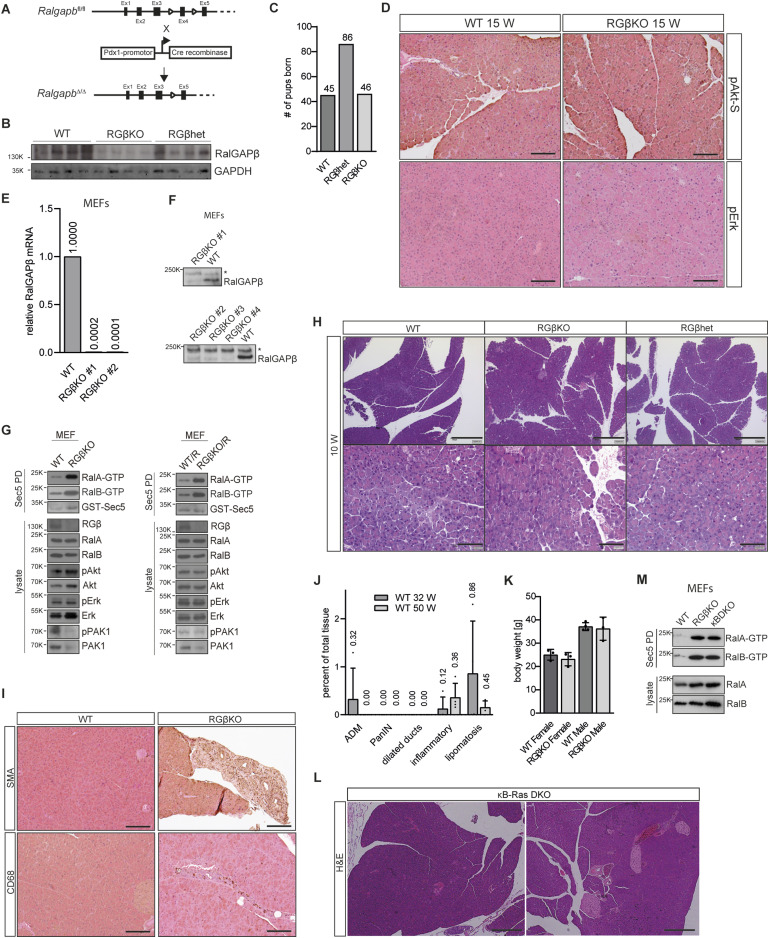
Pancreatic RalGAPβ deficiency promotes acinar-to-ductal metaplasia and leads to pancreatitis and neoplasia. **(A)** Schematic of conditional deletion of exon 4 of the RalGAPβ allele. **(B)** RalGAPβ protein levels in whole pancreas samples from four individual mice (7 wk) per genotype were analyzed by SDS–PAGE and immunoblot. **(C)** Analysis of the number of mice born with the indicated genotype. **(D)** pAkt substrate- and pErk-stained pancreas tissue sections of 15-wk-old WT and RGβKO mice with H&E counterstain. Scale bar = 200 μm. **(E)** qRT–PCR analysis of exon 2 deletion efficiency in RGβKO murine embryonic fibroblasts (MEFs) generated by CRISPR/Cas9 using a primer aligning within exon 2. Results are normalized to GAPDH mRNA levels. **(F)** Deletion of RalGAPβ was confirmed in different CRISPR RGβKO clonal lines via immunoblot. * unspecific band as a loading control. **(G)** RalA- and RalB-GTP levels were determined in WT and RGβKO MEFs with (right panel) and without (left panel) expression of exogenous KRas^G12D^ via GST-Sec5 pulldown, SDS–PAGE, and immunoblot. Input samples were blotted for activation of Akt, Erk, and PAK1 pathways. **(H)** H&E-stained pancreas tissue sections of 10-wk-old WT, RGβhet, and RGβKO mice. Top panel scale bar = 500 μm, bottom panel scale bar = 100 μm. **(I)** SMA- and CD68-stained pancreas tissue sections of 32-wk-old WT and RGβKO mice with H&E counterstain. Scale bar = 200 μm. **(J)** Quantification of the tissue area occupied by acinar-to-ductal metaplasia, PanIN lesions, dilated ducts, inflammation, and lipomatosis in 30 ± 2- and 50 ± 2-wk-old WT mice (30-wk WT: n = 4; 50-wk WT: n = 4). Periodic acid–Schiff (PAS)-stained pancreas sections. Given is mean with SD and individual values. **(K)** Bodyweight of female and male WT and RGβKO mice at 49–52 wk of age. n = 3 individual mice except WT male and n = 4 individual mice. Given is mean with individual values and SD. **(L)** Pancreas tissue sections of κB-Ras DKO animals (70 wk of age) were stained with H&E. Scale bar = 500 μm. **(M)** RalA- and RalB-GTP levels were determined in whole pancreas tissue samples from two individual mice per genotype (10–13 wk) via GST-Sec5 pulldown, SDS–PAGE, and immunoblot.

**Figure 1.**
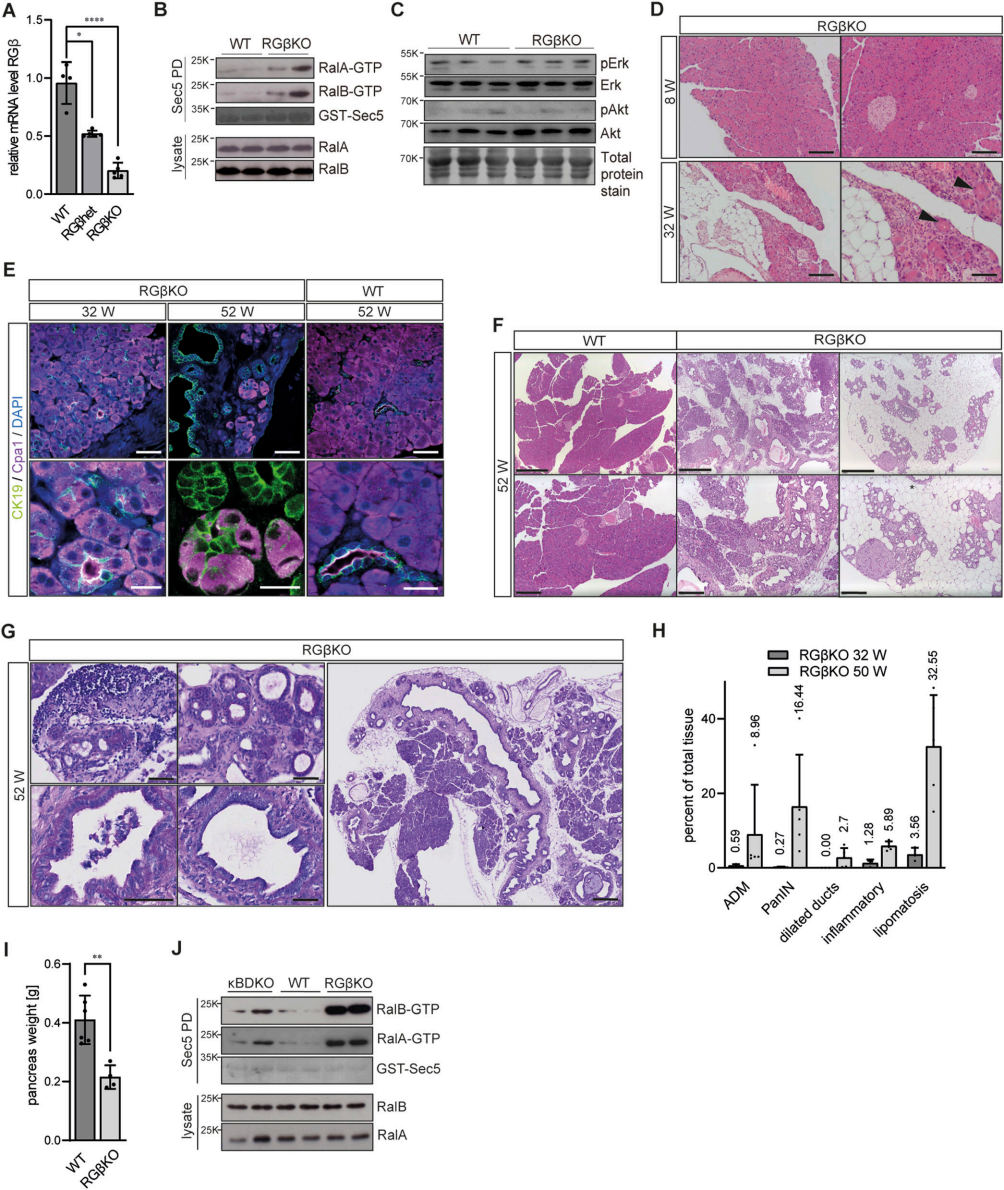
Pancreatic RalGAPβ deficiency promotes acinar-to-ductal metaplasia (ADM) and leads to pancreatitis and neoplasia. **(A)** mRNA levels of RalGAPβ were determined by qRT–PCR in whole pancreas samples (WT: n = 4; RGβKO and RGβhet: n = 5 individual mice; age: 7 wk). Given is mean with SD and individual values. *t* test: WT:RGβhet: *P = 0.0156; WT:RGβKO: ****P < 0.0001. **(B)** RalA- and RalB-GTP levels were determined in whole pancreas tissue samples from two individual mice (25 wk) per genotype via GST-Sec5 pulldown, SDS–PAGE, and immunoblot. **(C)** pErk, Erk, pAkt, and Akt protein levels in whole pancreas samples from three individual mice per genotype (11 wk of age) were analyzed by SDS–PAGE and immunoblot. **(D)** H&E-stained pancreas of RGβKO mice at the indicated age in weeks. Black arrows indicate ADM. Left panel scale bar = 100 μm, right panel scale bar = 50 μm. **(E)** Pancreatic sections of WT and RGβKO mice (32 and 52 wk) were stained for CK19 (cytokeratin-19, ductal) and Cpa1 (carboxypeptidase A1, acinar) and analyzed by confocal microscopy. Top panel scale bar = 50 μm, bottom panel scale bar = 20 μm. **(F)** H&E-stained pancreatic tissue sections of WT and two RGβKO mice at 52 wk of age. Top panel scale bar = 500 μm, bottom panel scale bar = 200 μm. **(G)** PAS staining of an RGβKO pancreas section (52 wk) shows examples of ADM and low-grade PanIN lesions with variable cellular mucin content and an overview panel (right). Left panel scale bar = 50 μm, right panel scale bar = 250 μm. **(H)** Quantification of the tissue area occupied by ADM, PanIN lesions, dilated ducts, inflammation, and lipomatosis in 30 ± 2- and 50 ± 2-wk-old RGβKO (30-wk RGβKO, n = 3; 50-wk RGβKO, n = 5) from periodic acid–Schiff (PAS)-stained pancreas sections. Given is mean with SD and individual values. WT analysis in Fig S1J. **(I)** Pancreas weight was determined after tissue resection from 52-wk-old WT (n = 6) and RGβKO (n = 4 individual mice) animals. Given is mean with SD and individual values. *t* test: **P = 0.0013. **(J)** RalA- and RalB-GTP levels were determined in whole pancreas tissue samples from two individual mice per genotype (10–13 wk) via GST-Sec5 pulldown, SDS–PAGE, and immunoblot.

To characterize the effects of reduced RalGAP complex activity in the pancreas, we crossed RalGAPβ floxed animals (Skorobogatko et al, 2018) with mice expressing Cre recombinase under control of the Pdx1 promoter (Gannon et al, 2000) (Fig S1A). The Pdx1 promoter drives the expression of Cre recombinase starting at day E8.5 in all cell types of the pancreas. As RalGAPβ is part of RalGAP1 and RalGAP2 complexes, this deletion interferes with GAP activity of both complexes toward Ral GTPases. Successful deletion of RalGAPβ was confirmed on both mRNA and protein levels in whole tissue samples (Figs 1A and S1B). Pancreatic RalGAPβ deficiency did not affect early viability, and conditional RalGAPβ-deficient mice (*Ralgapb*^fl/fl^ Pdx1-Cre^+^; RGβKO) were born at normal Mendelian ratios (Fig S1C). As anticipated, knockout of RalGAPβ led to a significant increase of RalA- and RalB-GTP levels in pancreatic tissue (Fig 1B). Activity of MAPK and PI3K/Akt signaling was not altered in RalGAPβ-deficient pancreas tissue (Figs 1C and S1D), demonstrating that the effect of RalGAP deletion is specific for Ral signaling downstream of Ras and no feedback mechanisms occur. This was confirmed in RalGAPβ-deficient murine embryonic fibroblasts (MEFs) that were generated by CRISPR/Cas9-mediated deletion of *Ralgapb* exon 3 (Fig S1E and F) and either did or did not express mutant KRas^G12D^ (Fig S1G). We did not detect an increase in phosphorylation of PAK1, which functions downstream of Rac1 GTPase. However, total levels of PAK1 were lower in RGβKO than in WT tissue, suggesting a possible impact on Rac1 signaling that warrants further investigation in the future.

Gross pancreatic architecture was not altered in young (<10 wk old) RGβKO animals when compared to heterozygous (*Ralgapb*^fl/+^ Pdx1-Cre^+^; RGβhet) or WT littermates (Figs 1D and S1H). However, starting from around 25–30 wk of age, we observed signs of ADM, immune infiltration, and lipomatosis in H&E-stained sections of RGβKO mice (Figs 1D and S1I). The presence of ADM lesions was confirmed by detecting the coexpression of cytokeratin-19 (CK19) and carboxypeptidase-A1 (Cpa1) by immunofluorescence staining (Fig 1E). This phenotype progressed with age (Fig 1E and F). At 50 ± 2 wk, we in addition observed dilated ducts, a hallmark of chronic pancreatitis (Esposito et al, 2020), as well as low-grade PanIN lesions, occupying on average about 16% of the total tissue area (Figs 1F–H and S1J). The extent of lipomatosis in RGβKO mice was variable (between 15% and 48% in the mice analyzed), but often affected whole pancreatic lobes with extensive replacement of the acinar tissue (Figs 1F and H and S1J). The pancreas weight of 52-wk-old RGβKO mice was thus reduced in comparison with littermate control animals (Fig 1I) because of pancreatic atrophy. Despite this extensive replacement of acinar cells, no body wasting was observed (Fig S1K), suggesting that the remaining acini were still sufficient to uphold an adequate level of nutrient uptake. This histological characterization demonstrates that loss of RalGAP activity alone is sufficient to induce ADM and neoplasia development in the adult pancreas, highlighting a potent impact of enhanced Ral GTPase signaling on tumor promotion in the pancreas.

Interestingly, pancreatic deletion of the RalGAP-regulating κB-Ras proteins (κB-Ras1/2 DKO) had not revealed any histological abnormalities up to an age of 25–30 wk (Beel et al, 2020). To confirm that we indeed detected a phenotypic difference between RalGAPβ and κB-Ras deficiencies, we examined κB-Ras1/2 DKO mice (κBDKO; *NKIRAS1*^−/−^ *NKIRAS2*^fl/−^ Pdx1-Cre^+^) up to an age of 70 wk. Also at this high age, κBDKO mice did not show any signs of pancreatitis or neoplasia development (Fig S1L). We found that RalGAPβ deficiency elicited a more potent induction of Ral activity in the pancreas than loss of κB-Ras proteins (Fig 1J). This could indicate that a certain level of Ral-GTP — only achieved by RalGAPβ loss — was required to trigger the observed phenotype. However, in κB-Ras1/2- and RalGAPβ-deficient murine embryonic fibroblasts Ral-GTP levels were enhanced to a comparable extent (Oeckinghaus et al, 2014) (Fig S1M). This demonstrated that RalGAPβ does not generally play a more important role for catalytic RalGAP activity. Rather, cell type–specific differences in the dependency on these two RalGAP complex components for control of total cellular Ral-GTP levels exist. A possible explanation for such a scenario would be that κB-Ras proteins are required for control of a subset of Ral functions that contribute differentially to total cellular Ral-GTP levels in distinct cell types.

### RalGAPβ deficiency deregulates polarized exocytosis of zymogen-containing vesicles

**Figure S2.**
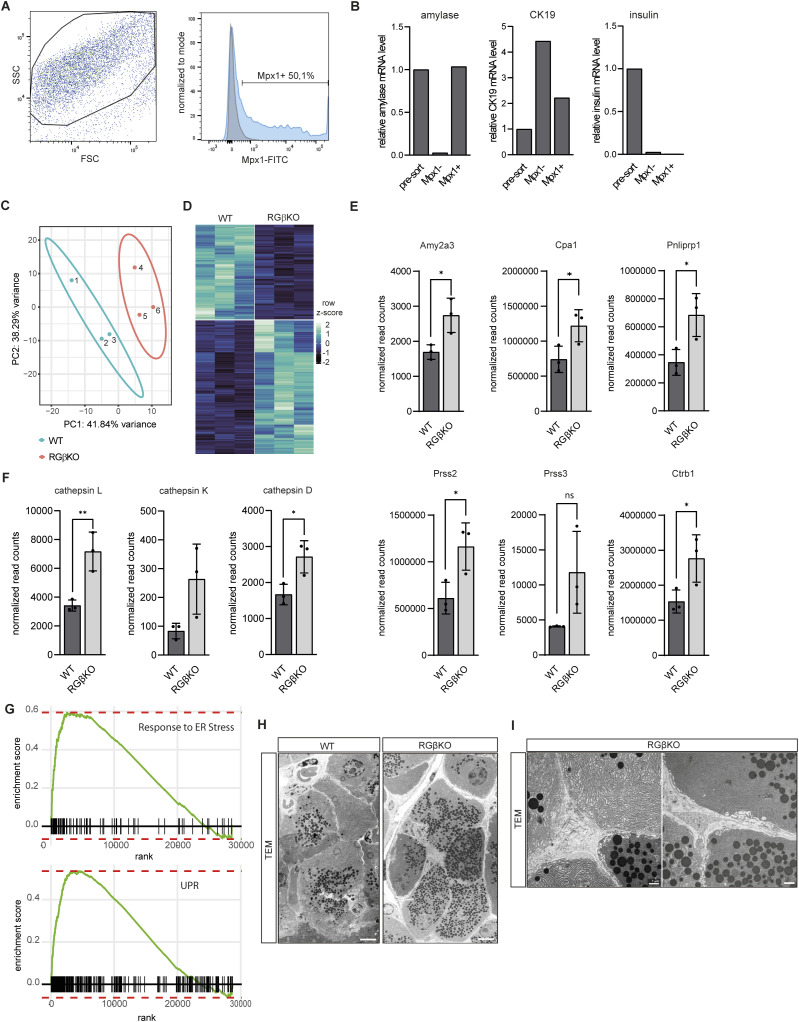
RalGAPβ deficiency deregulates polarized exocytosis of zymogen-containing vesicles. **(A)** Fluorescence-associated cell sorting gating strategy for isolation of primary acinar cells using MPx1 surface staining. **(B)** qRT–PCR analysis of amylase, CK19, and insulin mRNA levels in the sorted cell populations to demonstrate purity of the obtained cell samples. **(C)** Principal component analysis of RNA-sequencing samples obtained from fluorescence-associated cell sorting of acinar cells of WT (# 1–3) and RGβKO (# 4–6) mice. **(D)** Heatmap of row z-scored normalized read counts of differentially expressed genes (*P* < 0.05) between RGβKO and WT acinar cells (n = 3 mice per genotype). **(E)** Normalized read counts from RNA-sequencing analysis between RGβKO and WT acinar cells (n = 3 mice per genotype) for amylase 2a3 (Amy2a3), carboxypeptidase a1 (Cpa1), pancreatic lipase–related protein 1 (Pnliprp1), serine protease 2 (Prss2), serine protease 3 (Prss3), and chymotrypsinogen b1 (Ctrb1). Given is mean with SD and individual values. *t* test with Welch’s correction in case of unequal SDs for Amy2a3: *P = 0.0273, for Cpa1: *P = 0.0486, for Pnliprp1: *P = 0.0308, for Prss2: *P = 0.0349, for Prss3: ns *P* = 0.1492, for Ctrb1: *P = 0.0471. **(F)** Normalized read counts from RNA-sequencing analysis of RGβKO and WT acinar cells (n = 3 mice per genotype) for cathepsin L, cathepsin K, and cathepsin D. Given is mean with SDs and individual values. *t* test for cathepsin L: **P = 0.0097, cathepsin D: *P = 0.0270. **(G)** Enrichment plots for “response to endoplasmic reticulum stress” and “endoplasmic reticulum unfolded protein response” of gene ontology biological process (GOBP) analysis of RGβKO acinar cells in comparison with WT acinar cells. **(H)** Transmission electron microscopy image of acini from random-fed 12-wk-old WT and RGβKO mice reveals altered distribution of zymogen granules in acinar cells. Scale bar = 5 μm. **(I)** Transmission electron microscopy images of RGβKO acini (as in (H)) demonstrating localization of zymogen granules in close proximity to basolateral membranes of acinar cells. Scale bar = 1 μm.

**Figure 2.**
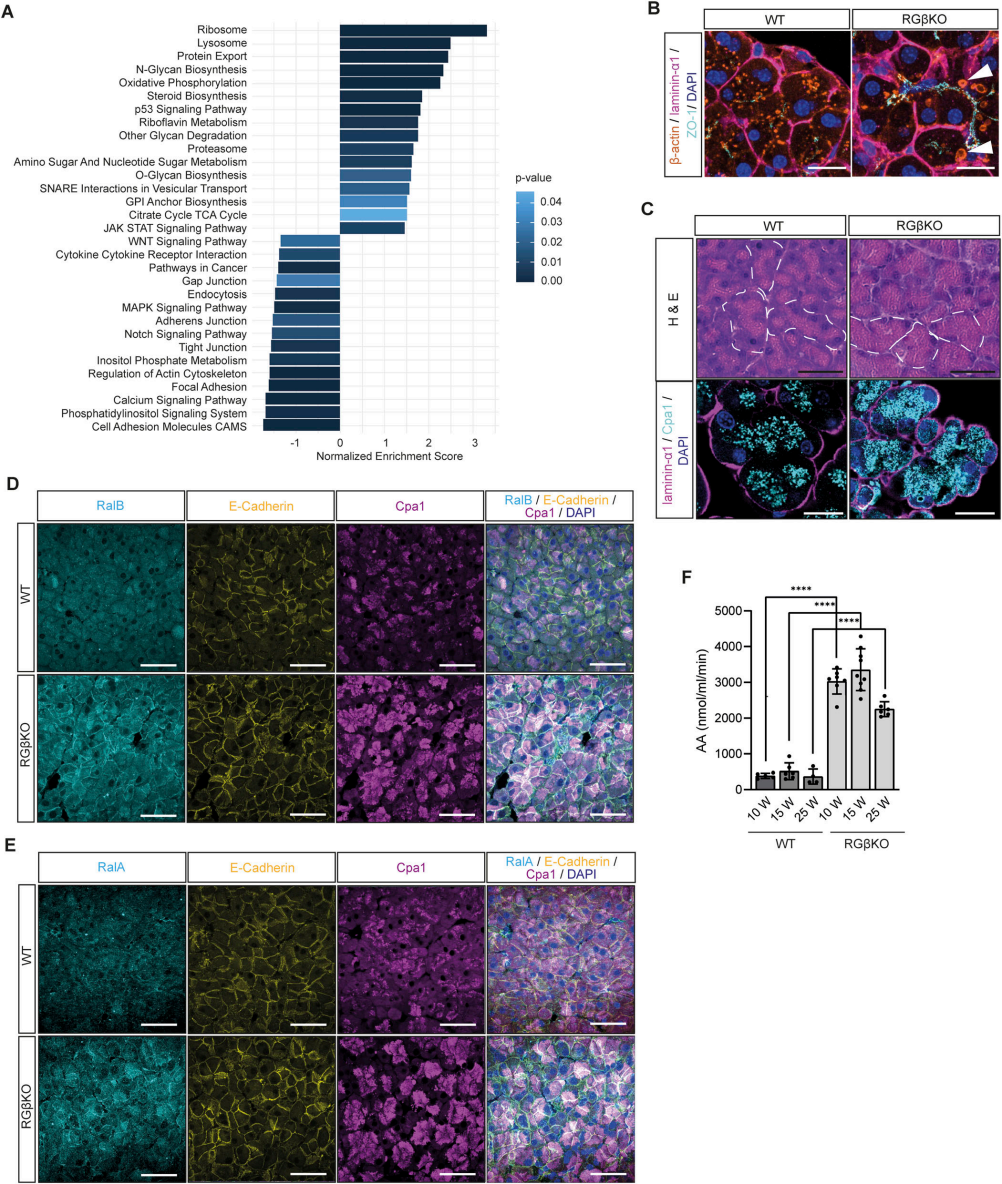
RalGAPβ deficiency deregulates polarized exocytosis of zymogen-containing vesicles. **(A)** KEGG pathway GSEA of primary RGβKO acinar cells in comparison with WT acinar cells with *P* < 0.05 and adjusted *P* < 0.25. n = 3 animals per genotype. **(B)** Pancreas sections from random-fed WT and RGβKO mice (13 wk) were stained for β-actin, laminin-α1, and ZO-1 and counterstained with DAPI to detect endocytic vacuoles (white arrows) by confocal immunofluorescence microscopy. Maximum intensity projection from z-stacks. Scale bar = 20 μm. **(C)** Pancreas sections of random-fed WT and RGβKO mice were H&E-stained (top panels) or stained for laminin-α1 (basal membrane) and Cpa1 (zymogen granules), counterstained with DAPI, and analyzed by confocal immunofluorescence microscopy. Maximum intensity projection from z-stacks. Top panel scale bar = 50 μm, bottom panel scale bar = 20 μm. **(D, E)** Pancreas sections of random-fed WT and RGβKO mice (15 wk) were stained for RalB (D) or RalA (E), Cpa1, E-cadherin (DAPI counterstain) and analyzed by confocal immunofluorescence microscopy. Maximum intensity projections of z-stacks. Scale bar = 50 μm. **(F)** Serum amylase levels were determined in random-fed WT and RGβKO mice of the indicated age. n = 4–9 individual mice per condition. Given is mean with SD and individual values. *t* test with Welch’s correction: **** all *P* < 0.0001.

We thus aimed to identify the molecular mechanisms underlying the phenotype of RalGAPβ-deficient mice. To this end, we first isolated primary acinar cells from young RGβKO mice and respective sex-matched littermate controls by fluorescence-associated cell sorting (FACS) (Dorrell et al, 2011) (Fig S2A) and performed RNA-seq analysis. Purity of the obtained cell population was hereby confirmed by qRT–PCR analysis of acinar, ductal, and Langerhans cell-specific marker gene expression (Fig S2B). We found 733 genes differentially expressed (*P* < 0.05), of which 429 genes were up- and 304 genes down-regulated in RGβKO acinar cells (Fig S2C and D). Gene set enrichment analysis (GSEA) revealed the KEGG pathways “ribosome,” “protein export,” and “SNARE interactions in vesicular transport” as significantly enriched in RGβKO cells (Fig 2A and Table S1). In addition, mRNA levels of digestive (pro)enzymes were up-regulated in RGβKO acinar cells (Fig S2E), suggesting the presence of a phenotype with enhanced exocytosis and secretion. Enrichment of the KEGG pathways “oxidative phosphorylation,” “riboflavin metabolism,” and “TCA cycle” hinted at a simultaneously enhanced metabolic activity of RGβKO cells. Further KEGG pathways enriched in RGβKO cells were “steroid biosynthesis,” “GPI anchor biosynthesis,” and “glycan biosynthesis,” all indicating a general deregulation of endoplasmic reticulum (ER)/Golgi-related processes connected to membrane protein synthesis. In line, our analysis also revealed an up-regulation of genes involved in lysosome function, including the lysosomal proteases cathepsin L, K, and D (Fig S2F), and a GO biological process (GOBP) gene signature connected to ER stress and the unfolded protein response (UPR) (Fig S2G and Table S2), hinting that RalGAPβ-deficient acinar cells might have to cope with overshooting protein production in the ER.

Table S1. KEGG pathway analysis of RGβKO acinar cells in comparison with WT acinar cells.

Table S2. GOBP pathway analysis of RGβKO acinar cells in comparison with WT acinar cells.

Based on these results, we first examined whether constitutively increased Ral activity upon RalGAPβ loss altered zymogen granule exocytosis, which represents the most prominent exocytic process in acinar cells. Indeed, we detected large, actin-coated endocytic vacuoles in RalGAPβ-deficient acinar cells (Fig 2B), indicative of an increased number of zymogen vesicles fusing during compound exocytosis, resulting in endocytic vacuoles that cannot be adequately cleared by actination and subsequent endocytosis (Chvanov et al, 2018; Voronina et al, 2022). This can be due to an increased number of zymogen granules or an enhanced exocytosis rate. In addition, we noticed in H&E-stained sections that eosinophilic zymogen granules were more diffusely localized in RalGAPβ-deficient acini (Fig 2C). WT controls showed the expected localization to apical regions. This was confirmed by immunofluorescence and transmission electron microscopy (TEM) of WT and RGβKO tissue sections (Figs 2C and S2H). Zymogen granules often filled the complete cellular lumen of RalGAPβ-deficient acinar cells (Fig S2H) and could be detected in close proximity to both basal and lateral membranes (Fig S2I). In addition, we observed an accumulation of RalA and RalB at both apical and basolateral acinar cell membranes in RGβKO but not WT animals (Fig 2D and E). This suggested that Ral-mediated processes, such as exocyst-mediated exocytosis, might be engaged aberrantly at all plasma membrane domains in the absence of RalGAP activity. Importantly, we detected high levels of amylase in the serum of unchallenged RGβKO animals (Fig 2F), which is likely driven by both enhanced and aberrantly basolaterally occurring exocytosis of digestive enzymes. Amylase serum levels were already enhanced in 10-wk-old RGβKO mice, at which age no gross alterations in tissue architecture, significant cell death, or inflammation were found. Abnormal exocytosis thus precedes and likely triggers the observed pancreatitis phenotype and does not represent a secondary effect caused by acinar cell death. Presumably, this phenomenon is not relevant prenatally as the secretory function of acinar cells is not active in utero (Uchiyama & Watanabe, 1984), explaining why phenotypic effects are detected in adult animals and progress with age. From these data, we conclude that RalGAP complex activity is involved in upholding spatial control of Ral activity in acinar cells, thus ensuring proper directionality of zymogen vesicle fusion. Interestingly, we had not observed changes in serum amylase levels in κBDKO mice (Beel et al, 2020), suggesting that functional differences must exist between loss of κB-Ras and RalGAPβ.

### RalGAPβ deficiency has broad effects on the secretory pathway and cell–cell contacts

**Figure 3.**
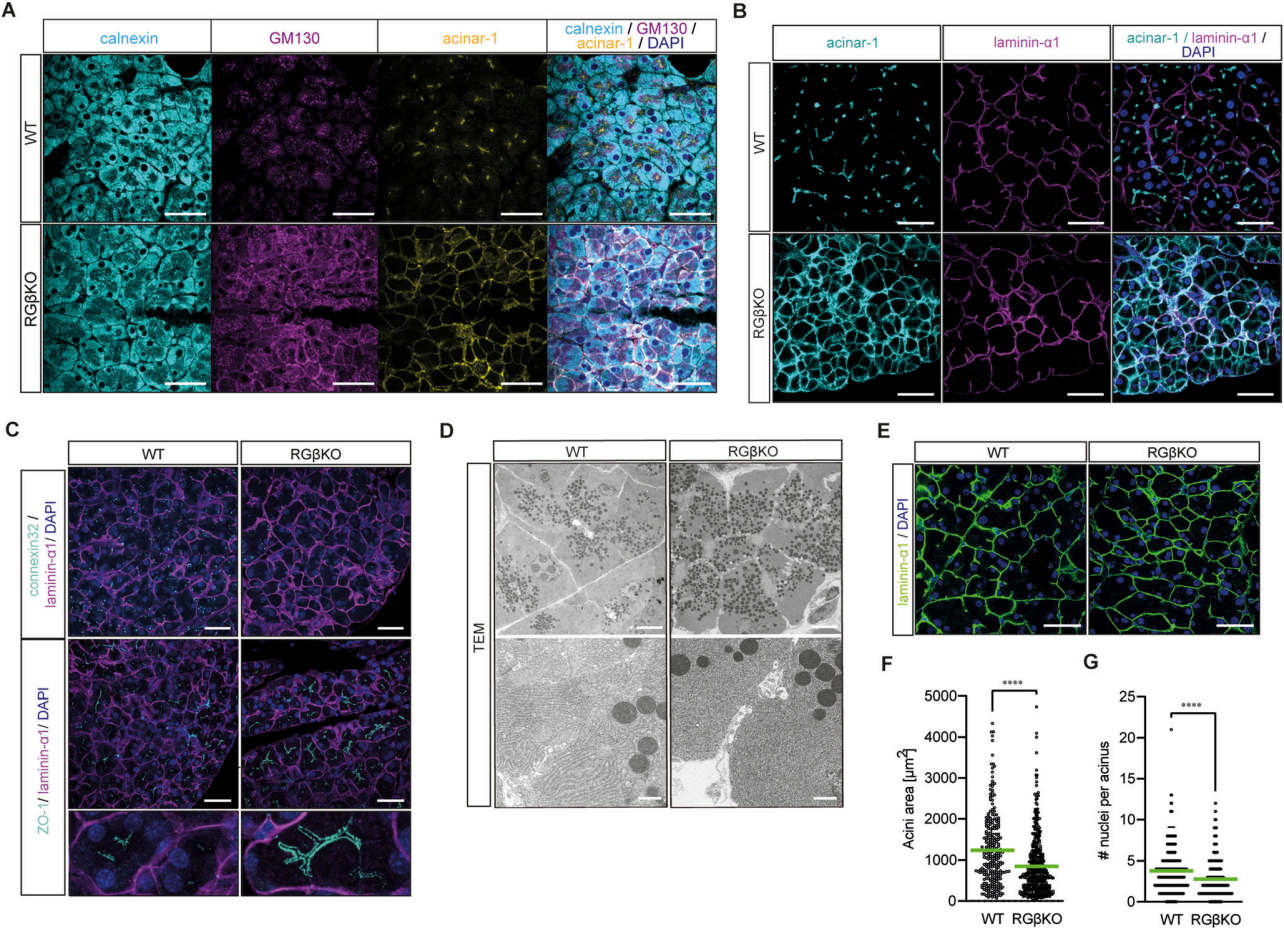
RalGAPβ deficiency has widespread effects on the secretory pathway and cell–cell contacts. **(A)** Pancreas sections of 15-wk-old WT and RGβKO mice were stained for calnexin (ER), GM130 (Golgi), and acinar-1 (apical acinar plasma membrane), counterstained with DAPI, and analyzed by confocal immunofluorescence microscopy. Maximum intensity projection. Scale bar = 50 μm. **(B)** Pancreas sections from WT and RGβKO mice (13 wk) were stained for acinar-1 (apical marker) and laminin-α1, counterstained with DAPI, and analyzed by confocal immunofluorescence microscopy. Scale bar = 50 μm. **(C)** Pancreas sections of 15-wk-old WT and RGβKO mice were stained for connexin-32 (gap junctions), ZO-1 (tight junctions), and laminin-α1 (basal acinar cell membrane), counterstained with DAPI, and analyzed by confocal immunofluorescence microscopy. Maximum intensity projections. Top panel scale bar = 50 μm, bottom panel scale bar = 200 μm. **(D)** Transmission electron microscopy images demonstrating enlarged acinus lumen that extends downward along basolateral membranes in the pancreas of a random-fed 12-wk-old RGβKO mouse in comparison with WT. Top panel scale bar = 5 μm, bottom panel scale bar = 1 μm. **(E)** Pancreas sections of 15-wk-old WT and RGβKO mice were stained for laminin-α1 (DAPI counterstain) and analyzed by confocal immunofluorescence microscopy. Scale bar = 50 μm. **(F)** Area of individual acini (as defined by laminin-α1 outline staining) was determined using QuPath. A minimum of 15 acini per image were quantified in five to six separate pictures of two individual mice per genotype. Given is mean and individual values for single acini. *t* test: ****P < 0.0001. **(G)** Number of nuclei per acinus was counted using QuPath. A minimum of 15 acini per image were analyzed in five to six separate pictures of two individual mice per genotype. Given is mean and individual values for single acini. *t* test: ****P < 0.0001.

**Figure S3.**
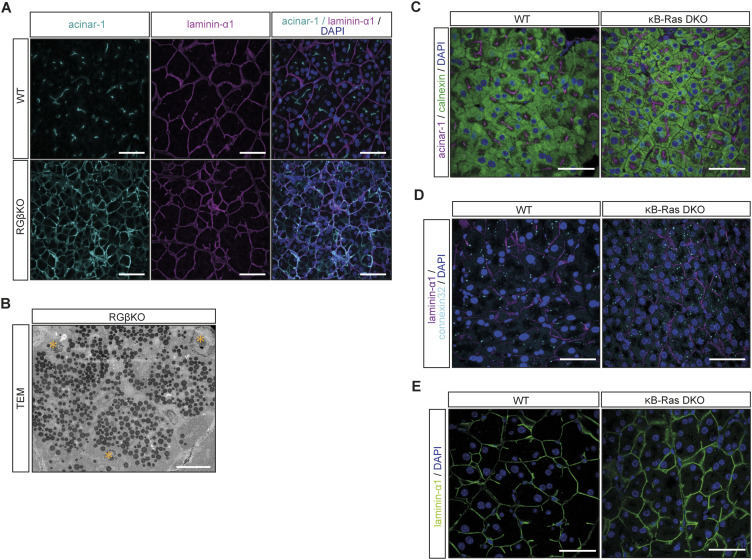
RalGAPβ deficiency has widespread effects on the secretory pathway and cell–cell contacts. **(A)** Pancreas sections from WT and RGβKO mice (4 wk) were stained for acinar-1 (apical marker) and laminin-α1, counterstained with DAPI, and analyzed by confocal immunofluorescence microscopy. Scale bar = 50 μm. **(B)** Transmission electron microscopy image of an acinus of a random-fed RGβKO mouse. Positions of nuclei are marked by yellow asterisks. **(C)** Pancreas sections from WT and κB-Ras DKO mice were stained for acinar-1 and calnexin (with DAPI counterstain) and analyzed by confocal immunofluorescence microscopy. Maximum intensity projection. Scale bar = 50 μm. **(D)** Pancreas sections of 15-wk-old WT and κB-Ras DKO animals were stained for laminin-α1 and connexin-32 (with DAPI counterstain) and analyzed by confocal fluorescence microscopy. Scale bar = 50 μm. **(E)** Pancreas sections of 15-wk-old WT and κB-Ras DKO animals were stained for laminin-α1 (with DAPI counterstain) and analyzed by confocal fluorescence microscopy. Scale bar = 50 μm.

To further characterize the impact of RalGAPβ loss on the secretory protein machinery in acinar cells, we visualized ER and Golgi in WT and RGβKO acinar cells via immunofluorescence microscopy of respective marker proteins calnexin and GM130 (Fig 3A). We detected no obvious differences in the intracellular localization of calnexin/ER. The normally Golgi-resident GM130, however, was partially localized to the plasma membrane in RGβKO cells. Furthermore, the apical membrane protein marker acinar-1 showed the expected exclusive apical localization in WT cells, but additional strong basolateral staining in RGβKO cells (Fig 3B). This phenomenon was already present in the mice before weaning age (Fig S3A). Basal positioning of nuclei was not affected (Fig S3B). Thus, in agreement with our RNA-seq analysis, RalGAPβ loss not only results in an up-regulation of zymogen vesicle secretion, but also impacts Golgi organization and acinar cell polarity.

In the list of KEGG pathways enriched in WT versus RGβKO acinar cells, terms connected to cell adhesion (especially tight and gap junction) were most prominent (Fig 2A). This led us to examine tissue sections for cellular contacts of RalGAPβ-deficient acinar cells, which also critically depend on a properly controlled secretory pathway. The tight junction protein ZO-1 was detected apically in WT and RGβKO acini, but was spread basolaterally away from the apices in RGβKO acini (Fig 3C). This demonstrates a lateralization of tight junctions, resulting in an enlarged lumen that extends downward along basolateral membranes. In line, TEM imaging detected a lateralization of microvilli and less-defined luminal spaces in the RalGAPβ knockout tissue (Fig 3D). Although tight junctions were mislocalized but present, gap junctions were almost completely lost in RGβKO acini in comparison with WT tissue as determined by connexin-32 (Cnx32) staining (Fig 3C). The loss of Cnx32-positive gap junctions is known to interfere with appropriate acinar cell–cell communication and to increase basal zymogen secretion in mice (Chanson et al, 1998; Frossard et al, 2003). Although we cannot explain the mechanism behind gap junction loss at this point, we hypothesize that it could be due to problems in connexon trafficking and/or assembly in the deregulated secretory pathway. We also detected a stronger deposition of laminin-α1 in the RalGAPβ-deficient pancreata with fewer laterally directed ramifications (Fig 3E). Generally, RalGAPβ-deficient acini (as defined by laminin-α1 staining of the basolateral membranes) were smaller in size. This could be attributed to a reduced cell number per acinus rather than a reduction in cell size (Fig 3E–G), again suggesting locally misguided secretion. Neither appropriate localization of the apical marker acinar-1, nor the presence of gap junctions or the level and localization of laminin deposition were altered in κBDKO pancreatic tissue (Fig S3C–E). This confirms that κB-Ras proteins are not essential to control the secretory pathway in acinar cells and suggests that RalGAP complexes exert this function in a κB-Ras–independent manner.

In summary, these experiments demonstrate that in pancreatic acinar cells, RalGAPβ deficiency not only enhances exocytosis of vesicles — as had already been previously described for GLUT4-containing vesicles in case of RalGAPβ loss in adipose tissue (Skorobogatko et al, 2018) — but also impacts protein sorting and cell polarity. We hypothesize that this causes misdelivery of both secreted and transmembrane proteins, thus affecting cell–cell contacts and the extracellular matrix.

### RalGAPβ deficiency prevents recovery of pancreatic tissue after mild acute pancreatitis

**Figure S4.**
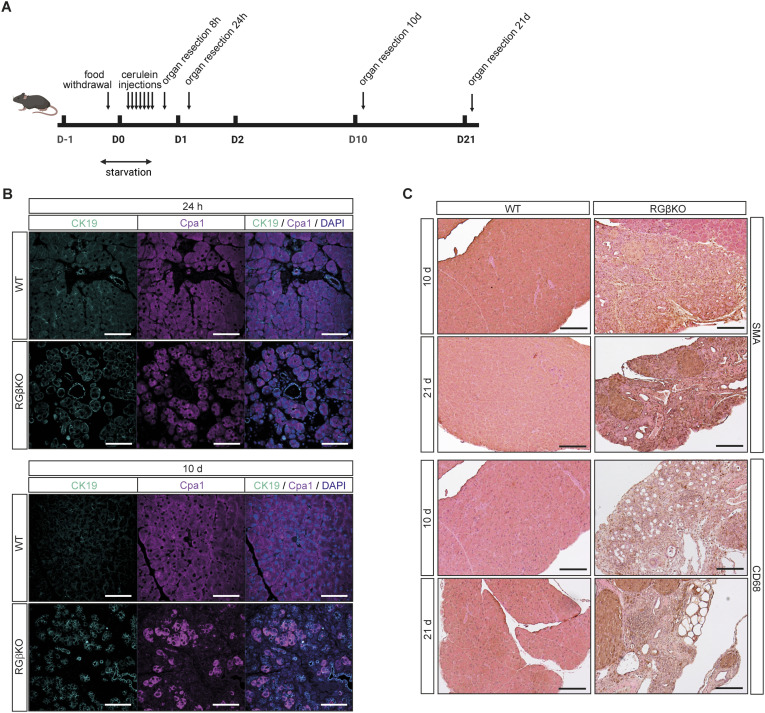
RalGAPβ deficiency prevents recovery of pancreatic tissue after mild acute pancreatitis. **(A)** Schematic of experimental flow of cerulein treatments and sample generation. Created with BioRender.com. **(B)** Pancreas sections of cerulein-treated (24 h or 10 d after the first injection) WT and RGβKO animals were stained for CK19 and Cpa1 (with DAPI counterstain) and analyzed by confocal fluorescence microscopy. Scale bar = 50 μm. **(C)** Pancreas sections of cerulein-treated (10 or 21 d after the first injection) WT and RGβKO mice were stained for SMA and CD68 with H&E counterstain. Scale bar = 200 μm.

**Figure 4.**
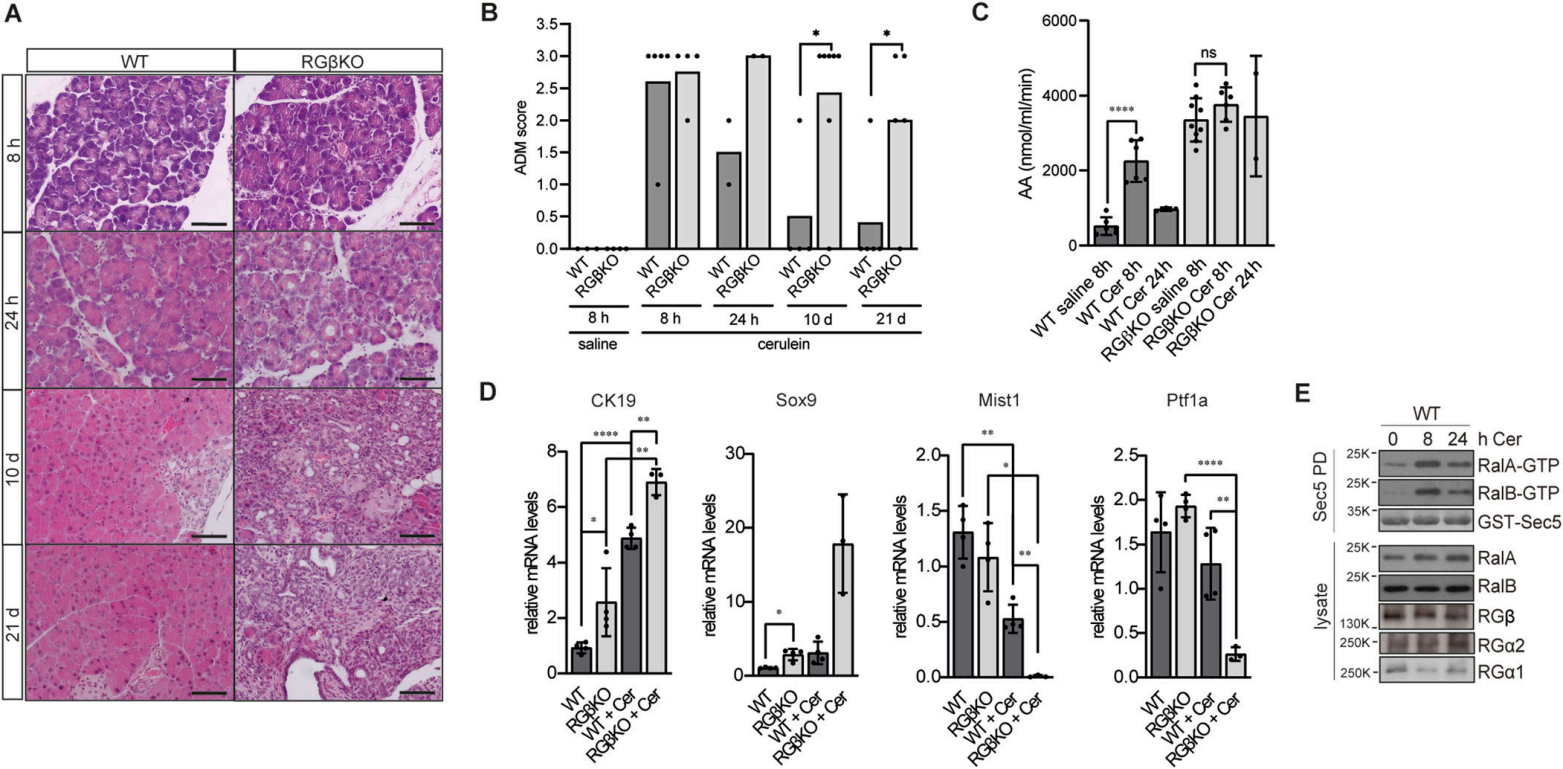
RalGAPβ deficiency prevents recovery of pancreatic tissue after mild acute pancreatitis. **(A)** 17- to 25-wk-old WT and RGβKO mice received 7 h cerulein injections. Pancreata were harvested and analyzed histologically 8 h, 24 h, 10 d, or 21 d after the first injection. Exemplary H&E-stained tissue sections are shown. Scale bar = 100 μm. **(B)** Acinar-to-ductal metaplasia (ADM) score documenting the percentage of tissue area affected by ADM of pancreata obtained from WT and RGβKO mice at 8 h, 24 h, 10 d, or 21 d after the first cerulein injection and saline-treated control animals. 0: ≤ 2%, 1: 2–15%, 2: 15–50%, 3: ≥ 50% of tissue area affected by ADM. Given is mean and individual values. *t* test: WT:RGβKO, 10 d: *P = 0.02; WT:RGβKO, 21 d: *P = 0.046. **(C)** Serum amylase levels were determined in WT and RGβKO mice 8 and 24 h after the first cerulein (Cer) injection and saline-treated control animals. n = 2–9 individual mice per condition. Given is mean with SD and individual values. *t* test with Welch’s correction in case of unequal SDs: WT:WT, 8-h Cer: ****P < 0.0001, RGβKO:RGβKO, 8-h Cer: ns *P* = 0.1709. **(D)** mRNA levels of CK19, Sox9, Mist1, and Ptf1a were determined by qRT–PCR in whole pancreas samples from WT and RGβKO mice obtained after cerulein (Cer) or saline injection. n = 4 individual mice per condition except RGβKO+Cer, n = 3. Given is mean with SD and individual values. *t* test with Welch’s correction in case of unequal SDs: CK19 (WT:RGβKO: *P = 0.381; WT:WT+Cer: ****P < 0.0001; RGβKO:RGβKO+Cer: **P = 0.0024; WT+Cer:RGβKO+Cer: **P = 0.0014), Sox9 (WT:RGβKO: *P = 0.0183), Mist1 (WT:WT+Cer: **P = 0.0012; RGβKO:RGβKO+Cer: *P = 0.0059; WT+Cer:RGβKO+Cer: **P = 0.0039), Ptf1a (RGβKO:RGβKO+Cer: ****P < 0.0001; WT+Cer:RGβKO+Cer: **P = 0.0082). **(E)** RalA- and RalB-GTP levels were determined in whole pancreas tissue samples from WT mice at 0, 8, and 24 h after the first cerulein injection via GST-Sec5 pulldown, SDS–PAGE, and immunoblot.

We next asked how RalGAPβ deficiency would affect outcome in case of an episode of acute pancreatitis. To induce a mild pancreatitis, young WT and RGβKO mice received repeated intraperitoneal injections with cerulein (Fig S4A). Induction of tissue injury and acinar regeneration were monitored histologically and scored in a blinded fashion based on the relative tissue area affected by ADM (score 0: 0–2%; score 1: 2–15%; score 2: 15–50%; score 3: >50%). The ADM score was slightly but not significantly higher in RGβKO mice in comparison with control littermates upon pancreatitis induction (Fig 4A and B), suggesting that RalGAPβ deficiency does not render the pancreas more susceptible to injury. Amylase levels were increased in serum taken from WT animals 8 h after the first cerulein injection, but could not be further enhanced in RGβKO animals (Fig 4C). Acinar regeneration was already evident in WT animals 24 h after the first cerulein injection, whereas tissue alterations further progressed in RGβKO mice (Figs 4A and S4B). qRT–PCR analysis of whole tissue samples demonstrated that the expression of key ductal genes such as CK19 and Sox9 was much more pronounced in RGβKO pancreas. In line, the expression of transcription factors that are central to upholding the acinar cell state, such as Mist1 and Ptf1a, was down-regulated more, confirming persistent ADM (Fig 4D). At 10 and 21 d after cerulein injections, pancreatic tissue in WT animals was mostly indistinguishable from the tissue of untreated mice. However, in RGβKO mice, we observed persistent ADM and inflammation that were accompanied by mild fibrosis and fatty replacement (Figs 4A and B and S4C), demonstrating that lack of RalGAP activity renders the cerulein-induced metaplastic process irreversible. We find that in WT animals, RalA- and RalB-GTP levels are enhanced in the acute ADM phase after cerulein injection, but decline when regeneration starts (Fig 4E), supporting a role of Ral GTPases during cerulein response. This phenotype of persistent ADM very closely resembled what we had previously observed for κBDKO mice (Beel et al, 2020). This led us to conclude that the RalGAPβ deficiency–specific loss of cell polarity and controlled exocytosis was not a prerequisite underlying the lack of acinar plasticity. Rather, a function of Ral GTPases that is controlled by κB-Ras–containing RalGAP complexes must be responsible for this aberrant regeneration phenotype. It is conceivable that deregulated exocytosis in RGβKO mice represents an internal ADM trigger, which in combination with a lack in plasticity results in the progressive accumulation of metaplastic lesions as observed in aging RGβKO mice.

### RalGAPβ deficiency enhances ADM via both major Ral downstream pathways

**Figure 5.**
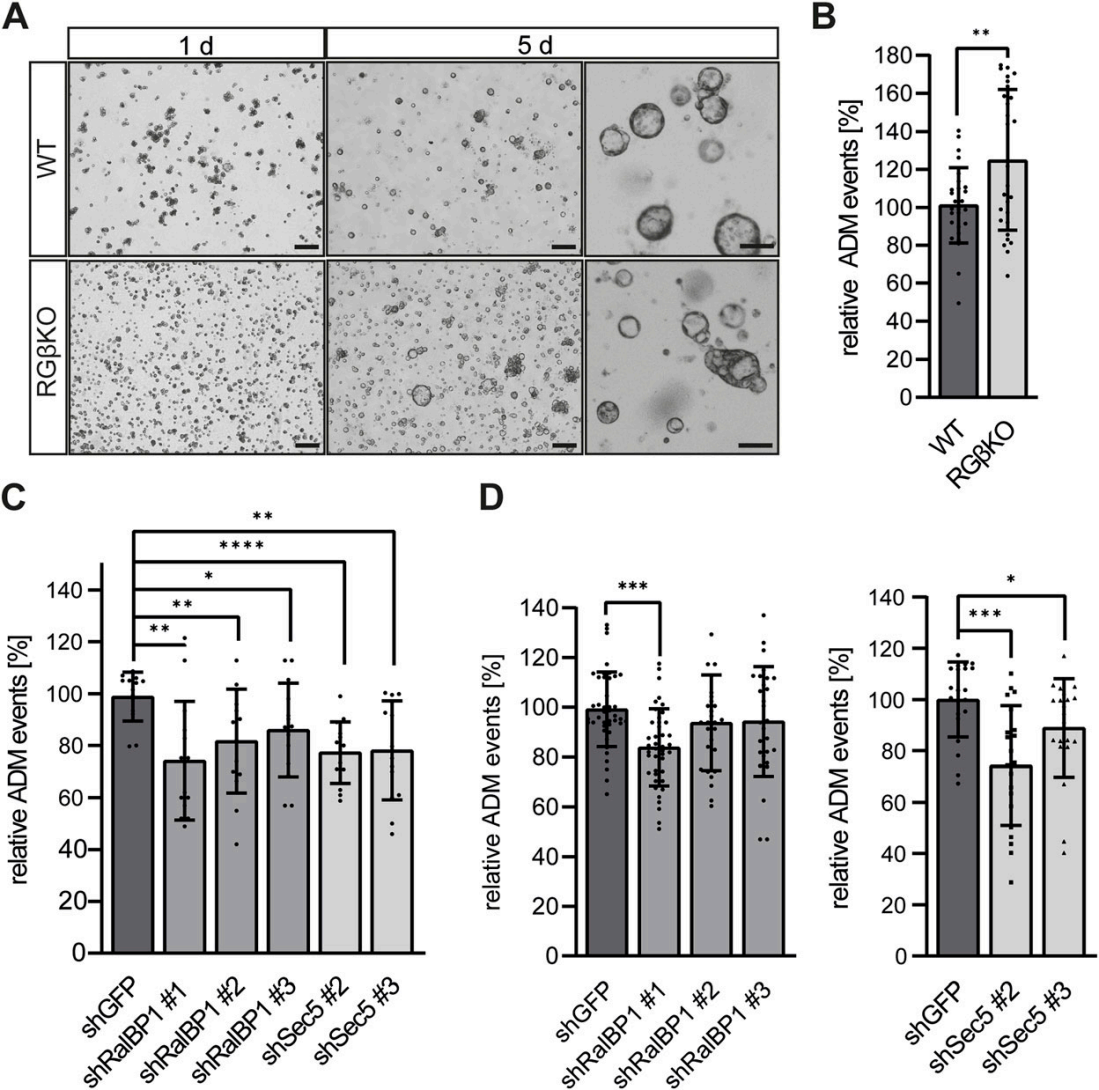
RalGAPβ deficiency enhances acinar-to-ductal metaplasia (ADM) via both major Ral downstream pathways. **(A)** Exemplary images of acini isolated from WT and RGβKO mice (9–16 wk old), after embedment in Cultrex BME for 1 and 5 d. Scale bars = 200 μm, right panel scale bars = 100 μm. **(B)** ADM events of acini from (A) were quantified relative to the total number of acini seeded after 5–6 d of 3D culture. The mean of the relative WT ADM events was set as 100%, and all percentages of ADM events are depicted normalized to the mean. n = 4 individual mice per condition in four individual experiments with seven fields of view analyzed per animal. Given is mean with SD and individual relative values. *t* test with Welch’s correction: **P = 0.0046. **(C)** Acini were isolated from 16- to 20-wk-old RGβKO mice. shRNA-mediated knockdown was achieved by lentiviral transduction. Acini were embedded in Cultrex BME and analyzed for ADM events after 6 d. The mean of the relative shGFP ADM events was set as 100%, and all percentages of ADM events are depicted normalized to the mean. n = 2 individual mice in two individual experiments with seven fields of view analyzed per animal. Given is mean with SD and individual relative values. *t* test with Welch’s correction in case of unequal SDs: shGFP:shRalBP1 #1: **P = 0.0016; shGFP:shRalBP1 #2: **P = 0.0091; shGFP:shRalBP1 #3: *P = 0.0283; shGFP:shSec5 #2: ****P < 0.0001; shGFP:shSec5 #3: **P = 0.0017. **(D)** Acini were isolated from KRas^G12D^-expressing mice (WT/R; 10–26 wk old). shRNA-mediated knockdown was achieved by lentiviral transduction. Acini were embedded in Cultrex BME and analyzed for ADM events after 5–6 d. The mean of the relative shGFP ADM events was set as 100%, and all percentages of ADM events are depicted normalized to the mean. n = 6 individual mice in individual experiments for shRalBP1 #1 and 4 repeats for shRalBP1 #2 and #3 with 7 fields of view analyzed per animal. Given is mean with SD and individual relative values. *t* test with Welch’s correction in case of unequal SDs: shGFP:shRalBP1 #1: ***P < 0.001. n = 3 individual mice in individual experiments for shSec5 #2 and #3 with seven fields of view analyzed per animal. Given is mean with SD and individual relative values. *t* test with Welch’s correction in case of unequal SDs: shGFP:shSec5 #2: ***P = 0.0001; shGFP:shSec5 #3: *P = 0.0425.

**Figure S5.**
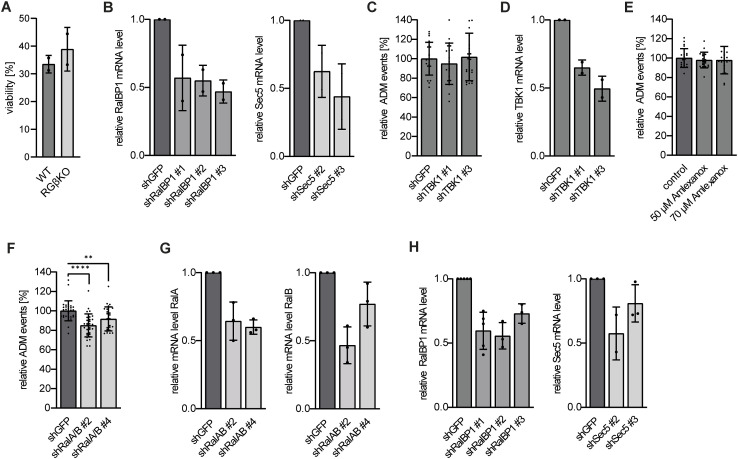
RalGAPβ deficiency enhances acinar-to-ductal metaplasia (ADM) via both major Ral downstream pathways. **(A)** In Cultrex BME-embedded WT and RGβKO acini were isolated from the matrix by enzymatic digestion and stained with trypan blue solution, and live cells were counted in a Neubauer chamber (n = 2). **(B)** Relative mRNA levels were determined by qRT–PCR in RGβKO acini at 72 h after lentiviral transductions introducing several shRNAs targeting shRalBP1 (left panel) or Sec5 (right panel). n = 2 individual mice in two individual experiments. Given is mean with SD and individual relative values. **(C)** Acini were isolated from 10- to 18-wk-old KRas^G12D^-expressing mice. shRNA-mediated knockdown was achieved by lentiviral transduction. Acini were embedded in Cultrex BME and analyzed for ADM events after 5–6 d. The mean of the relative shGFP ADM events was set as 100%, and all percentages of ADM events are depicted normalized to the mean. n = 3 individual mice per condition in three individual experiments with seven fields of view analyzed per animal. Given is mean with SD and individual relative values. **(D)** Relative TBK1 mRNA levels were determined by qRT–PCR in KRas^G12D^-expressing acini 72 h after lentiviral transductions. n = 2 individual mice in two individual experiments. Given is mean with SD and individual relative values. **(E)** Acini were isolated from 15- to 17-wk-old KRas^G12D^-expressing mice, embedded in Cultrex BME with 50 or 70 μM amlexanox, and analyzed for ADM events after 5 d. The mean of the relative control ADM events was set as 100%, and all percentages of ADM events are depicted normalized to the mean. n = 3 individual mice in three individual experiments with seven fields of view analyzed per animal. Given is mean with SD and individual relative values. **(F)** Acini were isolated from 14- to 26-wk-old KRas^G12D^-expressing mice. shRNA-mediated knockdown was achieved by lentiviral transduction. Acini were embedded in Cultrex BME and analyzed for ADM events after 5–6 d. The mean of the relative control ADM events was set as 100%, and all percentages of ADM events are depicted normalized to the mean. n = 5 individual mice per condition for shRalA/B #2, n = 4 for shRalA/B #4 with seven fields of view analyzed per animal. Given is mean with SD and individual relative values. *t* test shGFP:shRalA/B #2: ****P < 0.0001; shGFP:shRalA/B #4: **P = 0.0064. **(G)** Relative RalA (left panel) and RalB (right panel) mRNA levels were determined by qRT–PCR in KRas^G12D^-expressing acini 72 h after lentiviral transductions. n = 3 individual mice in three individual experiments. Given is mean with SD and individual relative values. **(H)** Relative mRNA levels were determined by qRT–PCR in KRas^G12D^-expressing acini 72 h after lentiviral transductions introducing shRNAs targeting RalBP1 (left panel) or Sec5 (right panel). n = 3 individual mice in three individual experiments. Given is mean with SD and individual relative values.

We next tested whether we could also observe enhanced and/or persistent metaplasia in an in vitro ADM assay system, where metaplasia occurs independent of the organ environment. Primary acini were retrieved through careful enzymatic digestion and seeded in a 3D Cultrex extracellular matrix, which triggers metaplastic conversion of acini to duct-like ring structures. Analyzing conversion events relative to the total number of acinar clusters, we found that RalGAPβ-deficient acini formed duct-like ring structures more efficiently than WT acini (Fig 5A and B). Differences in cell viability between WT and RalGAPβ-deficient acini were not observed (Fig S5A), suggesting that this phenomenon was independent of cell injury. We noted that despite similar digestion conditions, RGβKO acini were smaller in size than those from WT animals (Fig 5A), which correlates to the in vivo observations (see also Fig 3E–G). The observed increase in transdifferentiated acini in RGβKO samples in this system was rather mild, which we hypothesize to be due to the fact that like in the in vivo model, RalGAPβ deficiency might affect ADM regeneration more than induction. Indeed, similar 3D culture systems have been shown to be sensitive to interference with regenerative pathways, for example, primary cilium formation (Bangs et al, 2020). Although we cannot conclude from our dataset whether generation or regeneration of ADM is affected by RalGAPβ deficiency, our results demonstrate, that the effect of RalGAPβ deficiency on ADM was cell-intrinsic and occurred independent of potential effects from the organ environment.

We used this in vitro system to ask which effectors might be involved in the ADM function of Ral GTPases. We used shRNAs to down-regulate the main Ral effectors, RalBP1 and Sec5, in primary acini via lentiviral transduction and then assayed the ability of acini to form duct-like structures. Knockdown efficiencies of ∼50% could be achieved in this 3D culture system (Fig S5B), which for both effectors led to a significant impairment in the formation of duct-like ring structures by RGβKO acini (Fig 5C). This suggests that both Ral downstream functions can contribute to upholding an ADM state in this in vitro system. Knockdown or amlexanox-mediated inhibition of TBK1, which has been reported as an exocyst-independent signaling mediator downstream of Sec5 engagement, had no effect on ADM in this assay (Fig S5C–E). This suggests relevance of exocyst function downstream of Sec5. To our knowledge, an involvement of the exocyst in ADM control has not been explored to date. The small GTPase Rac1, for which RalBP1 harbors GAP activity, has previously been implicated in actin reorganization and morphological changes during ADM (Heid et al, 2011). Although Rac1 activation has been attributed mainly to PI3K signaling in this context (Wu et al, 2014), it is conceivable that the Ral/RalBP1/Rac axis might also be relevant in controlling Rac activity. With respect to pancreatic tumor development, we obtained similar results when we knocked down Ral GTPases, RalBP1 or Sec5 in KRas^G12D^-expressing acini (Figs 5D and S5F–H), highlighting that these Ral-regulated processes are also important in the case of (persistent) ADM initiated by oncogenic KRas mutation.

In summary, our results confirm that up-regulation of Ral GTPase activity promotes ADM in a cell-intrinsic fashion and highlight that engagement of at least two Ral downstream pathways has the potential to support this function in vitro.

### Primary cilium formation is defective in both RalGAP- and κB-Ras–deficient cells

**Figure S6.**
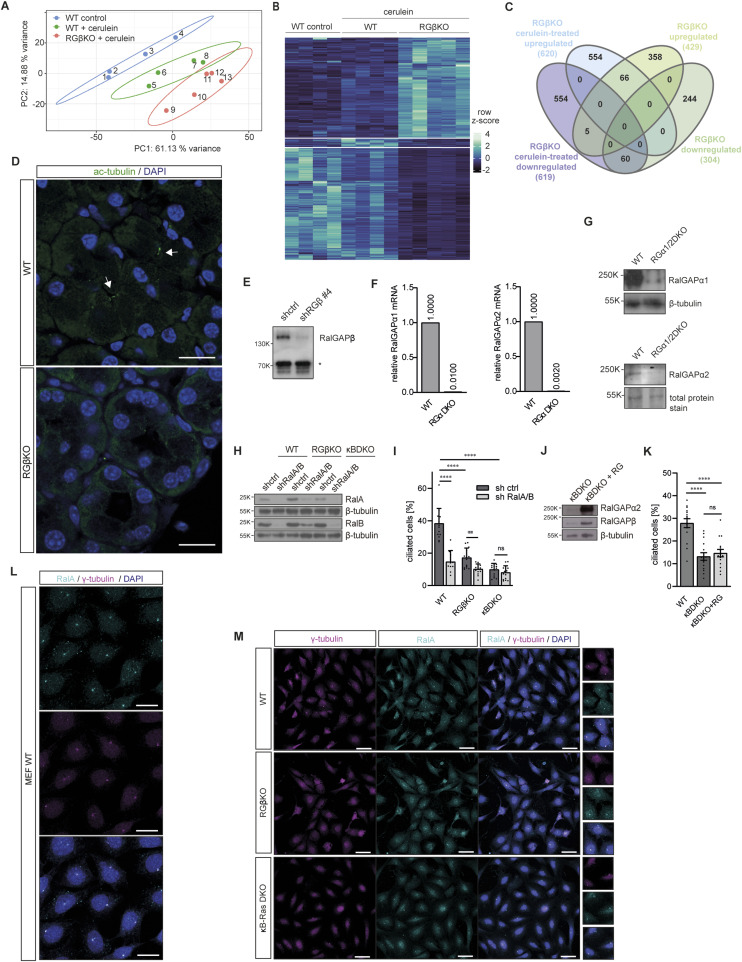
Primary cilium formation is defective in both RalGAP- and κB-Ras–deficient cells. **(A)** Principal component analysis of RNA-sequencing samples obtained from fluorescence-associated cell sorting of acinar cells of 0.9% saline control–treated WT mice (#1–4), cerulein-treated WT mice (#5–8), and cerulein-treated RGβKO mice (#8–13). Samples were isolated 24 h after the first cerulein injection. **(B)** Heatmap of row z-scored normalized read counts of differentially expressed genes (*P* < 0.05) in primary acinar cells obtained via fluorescence-associated cell sorting of digested pancreatic tissue from 0.9% saline control–treated WT mice (#1–4), cerulein-treated WT mice (#5–8), and cerulein-treated RGβKO mice (#8–13). Heatmap was generated with the differentially expressed genes from the cerulein-treated samples. **(C)** Venn diagram comparing significantly (*P* < 0.05) up- and down-regulated differentially expressed genes between the cerulein-treated and unchallenged RGβKO acinar cells by gene names. **(D)** Pancreas sections from WT and RGβKO mice were stained for acetylated tubulin with DAPI counterstain and analyzed by confocal immunofluorescence microscopy. Scale bar = 20 μm. **(E)** Stable RGβ knockdown MEFs, generated through lentiviral transduction, were lysed, and the expression level of RalGAPβ was analyzed by immunoblot. * unspecific band as a loading control. **(F)** qRT–PCR analysis of the deletion efficiency of RGα1 and RGα2 exons in generated clonal CRISPR MEF cell line using primers that align within the deleted exon. **(G)** RGαDKO MEFs were lysed and expression levels of RGα1 and RGα2 analyzed by immunoblot. **(H)** RalA and RalB (shRalA/B #4) were knocked down in WT, RGβKO, and κBDKO MEFs via lentiviral transduction. The shRalA/B MEFs were lysed with respective control MEFs (shctrl), and expression levels of RalA and RalB were analyzed by immunoblot. **(I)** MEFs (as in (H)) were serum-starved for 48 h, stained for γ-tubulin and Arl13b, and analyzed by confocal immunofluorescence microscopy. Cells with detectable primary cilia were counted in z-stack maximum intensity projections from five pictures per cell line. n = 3 independent experiments. Given is mean and individual values with SD. *t* test with Welch’s correction in case of unequal SDs: WT shctrl:WT shRalA/B: ****P < 0.0001; WT shctrl:RGβKO shctrl: ****P < 0.0001; WT shctrl: κBDKO shctrl: ****P < 0.0001; RGβKO shctrl:RGβKO shRalA/B: ***P = 0.0006; κBDKO shctrl:κBDKO shRalA/B: ns *P* = 0.2303. **(J)** RalGAP2 complex (RG) was overexpressed in κBDKO MEFs by transfection with pITR-ALFA-RalGAPβ and pITR-ALFA-RalGAPα2 plasmids. The cells were lysed, and expression levels of RalGAPβ and RalGAPα2 were analyzed by immunoblot. **(K)** MEFs (as in (J)) were serum-starved for 48 h, stained for γ-tubulin and Arl13b, and analyzed by confocal immunofluorescence microscopy. Cells with detectable primary cilia were counted in z-stack maximum intensity projections from five pictures per cell line. n = 3 independent experiments. Given is mean and individual values with SD. *t* test: WT:κBDKO: ****P < 0.0001; WT: κBDKO+RG: ****P < 0.0001; κBDKO: κBDKO+RG: ns *P* = 0.5369. **(L)** WT MEFs were serum-starved for 48 h, stained for RalA and γ-tubulin, and analyzed by confocal immunofluorescence microscopy. Cells were counterstained with DAPI. Scale bar = 20 μm. **(M)** WT, RGβKO, and κB-Ras DKO MEFs were serum-starved for 48 h, stained for RalA and γ-tubulin, and analyzed by confocal immunofluorescence microscopy. Cells were counterstained with DAPI. n = 3 independent experiments. Scale bar = 50 μm.

**Figure 6.**
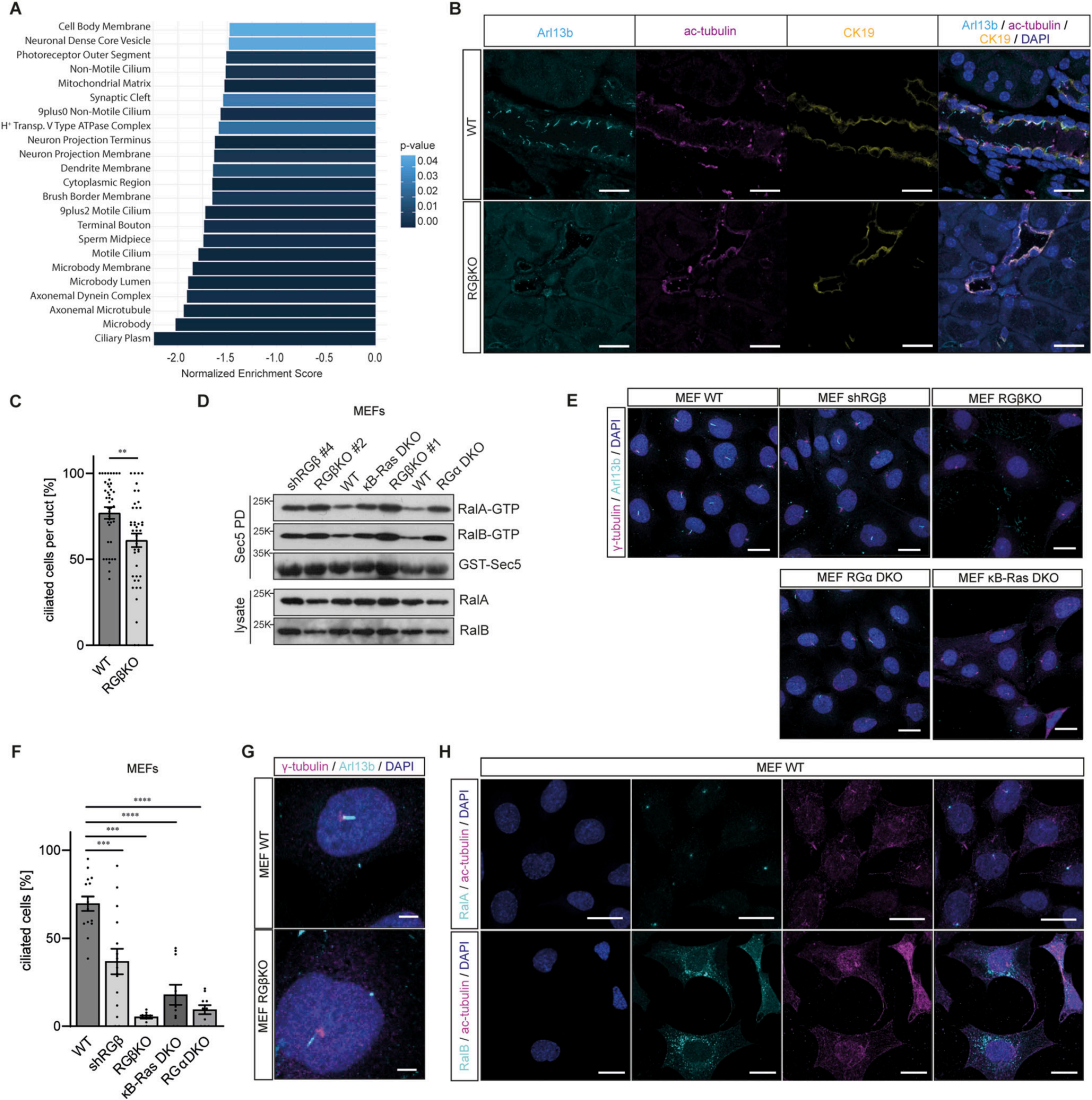
Primary cilium formation is defective in both RalGAP- and κB-Ras–deficient cells. **(A)** Gene ontology cellular component (GOCC) analysis of cerulein-treated RGβKO in comparison with WT acinar cells with *P* < 0.05 and *Q* < 0.25. n = 4 WT, n = 5 RGβKO mice. **(B)** Pancreas sections from 13- to 15-wk-old mice were stained to visualize primary cilia via detection of Arl13b and acetylated tubulin. CK19 staining was performed to identify pancreatic ducts. Sections were analyzed by confocal immunofluorescence microscopy. Maximum intensity projection. Scale bar = 20 μm. **(C)** Ducts were analyzed in Arl13b-, acetylated tubulin–, and CK19-stained pancreas sections from WT and RGβKO mice by confocal immunofluorescence microscopy. Ductal primary cilia were counted in z-stack maximum intensity projections from 10 pictures per mouse yielding 43 ducts per genotype. Given is mean with SD and individual values. *t* test: **P = 0.0028. n = 3 mice per genotype. **(D)** RalA- and RalB-GTP levels were determined in WT, RGβKO, shRGβ, κB-Ras DKO, and RGα DKO MEFs via GST-Sec5 pulldown and immunoblot. **(E)** WT, RGβKO, shRGβ, κB-Ras DKO, and RGα DKO MEFs were serum-starved for 48 h, stained for γ-tubulin and Arl13b, and analyzed by confocal immunofluorescence microscopy. Scale bar = 20 μm. **(F)** WT, RGβKO, shRGβ, κB-Ras DKO, and RGα DKO MEFs were serum-starved for 48 h, stained for γ-tubulin and Arl13b, and analyzed by confocal immunofluorescence microscopy. Cells with detectable primary cilia were counted in z-stack maximum intensity projections from five pictures per cell line. Given is percentage of ciliated cells of total cells present in each field of view. n = 3 independent experiments for WT and shRGβ, n = 2 for all other genotypes. Given is mean and individual values with SD. *t* test with Welch’s correction in case of unequal SDs: WT:RGβKO: ****P < 0.0001; WT:shRGβ: ***P = 0.0007; WT:RGαDKO: ****P < 0.0001; WT:κBRas DKO: ****P < 0.0001. **(G)** Enlarged view of cells as in (E). Scale bar = 5 μm. **(H)** WT MEFs were serum-starved for 48 h and stained for acetylated tubulin and RalA (upper panel) or RalB (lower panel). Scale bar = 20 μm.

To identify deregulated processes relevant for regeneration, we isolated primary acinar cells from cerulein-injected RGβKO mice and respective sex-matched littermate controls by FACS (Dorrell et al, 2011) and performed RNA-seq analysis. We investigated changes in gene expression signatures during acinar regeneration, comparing WT and acinar cells isolated 24 h after the start of the cerulein treatment. We found 1,239 annotated genes differentially expressed (*P* < 0.05), of which 620 genes were up- and 619 genes down-regulated in cerulein-treated RGβKO acinar cells (Fig S6A and B). Comparison of the differentially regulated genes in the unchallenged and cerulein-treated RGβKO acinar cells revealed an overlap of only 66 genes for the up-regulation and 60 genes for the down-regulation (Fig S6C), confirming that most of the differentially expressed genes were affected by the cerulein treatment and not predisposed by the RGβKO genotype. Interestingly, gene set enrichment analysis revealed a prominent down-regulation of GO cellular component (GOCC) terms associated with assembly of the primary cilium (e.g., ciliary plasm, axonemal microtubule, axonemal dynein complex, nonmotile cilium) (Fig 6A and Table S3). The primary cilium is an antenna-like sensory organelle that responds to mechanical and chemical stimuli and is critical for the regulation of signaling during development, organ maintenance, and regeneration (Fry et al, 2014; Mill et al, 2023). Unlike duct cells and islets of Langerhans cells, acinar cells do not possess a primary cilium under physiological conditions. However during ADM, the transdifferentiated duct-like cells assemble a primary cilium, which acts as a critical signaling hub for developmental pathways required for acinar regeneration (Seeley et al, 2009). In line, deficiency of proteins necessary for primary cilium formation has been reported to result in chronic pancreatitis, cyst formation, and lipomatosis (Cano et al, 2004, 2006; Augereau et al, 2016). Based on this gene expression analysis, we performed histological stainings and detected primary cilia in WT but not RGβKO acini 24 h after cerulein treatment (Fig S6D). However, primary cilium numbers were so low, likely because of the late time point of analysis, that a reliable quantification was not possible. We therefore decided to analyze whether we could detect a defect in primary cilium formation in the RalGAPβ-deficient duct cells in unchallenged mice, assuming that such a role of Ral GTPase signaling would likely be conserved between cell types. Indeed, the number of ductal cells with primary cilia was significantly reduced in RGβKO animals (Fig 6B and C).

Table S3. GOCC pathway analysis of cerulein-treated RGβKO acinar cells in comparison with treated WT acinar cells.

To further corroborate this result, we examined RalGAPβ knockout MEFs and MEFs with a stable knockdown of RalGAPβ generated via lentiviral transduction (Fig S6E), both of which led to a comparable increase in Ral-GTP levels (Fig 6D). Cells were serum-starved for 48 h to induce primary cilium formation. Both RalGAPβ-lacking cell lines developed significantly fewer primary cilia than WT MEFs (Fig 6E and F). Also, the cilia that were detectable appeared thinner and shorter than those found in WT cells (Fig 6G). To confirm that this effect was due to a GAP-mediated effect toward Ral GTPases and not a RalGAP complex–independent function of RalGAPβ, we generated RGα1/2 double-deficient (RGα DKO) MEFs by sequential CRISPR/Cas9-mediated gene editing (Fig S6F and G). Double deficiency of both RalGAPα subunits led to a comparable increase in Ral-GTP levels as loss of RalGAPβ or κB-Ras in MEFs (Fig 6D), again highlighting that in MEFs, almost all Ral activity detected depends on the presence of κB-Ras proteins. RGα DKO cells also showed a decreased ability to form primary cilia upon starvation (Fig 6E and F). Importantly, we also observed hampered primary cilium formation in κB-Ras DKO MEFs upon starvation (Fig 6E and F), demonstrating that this function of RalGAP/Ral signaling requires the presence of κB-Ras proteins. Knockdown of RalA and RalB in WT and RGβKO MEFs reduced primary cilium formation (Fig S6H and I). Thus, primary cilia cannot form when Ral GTPases are absent, but their formation is also disturbed when Ral-GTP levels cannot be properly regulated. No significant effect of Ral knockdown was observed in κBDKO MEFs (Fig S6H and I), likely because of the already very low primary cilium levels in these cells. Overexpression of RalGAPβ:RalGAPα2 in κB-Ras1/2-deficient MEFs was not able to rescue primary cilium formation (Fig S6J and K), suggesting that κB-Ras proteins are essential for this function of the RalGAP complexes. We therefore hypothesize that κB-Ras association might anchor RalGAP complexes to the growing cilia to control subcellular Ral activity at this organelle. Unfortunately, antibodies to probe this hypothesis by staining endogenous proteins are lacking. In contrast, RalGAP/Ral function in secretion occurs independently of κB-Ras proteins. Based on the facts that (1.) defective primary cilium formation is common to both κB-Ras and RalGAP knockout systems and that (2.) primary cilia have a known role in organ regeneration as initiating hub for regenerative signaling such as Hedgehog and Notch signaling (Lodh et al, 2014), we suggest that defective primary cilium formation underlies the impaired acinar regeneration capabilities of RalGAPβ- and κB-Ras1/2–deficient mice after acute pancreatitis.

**Figure S7.**
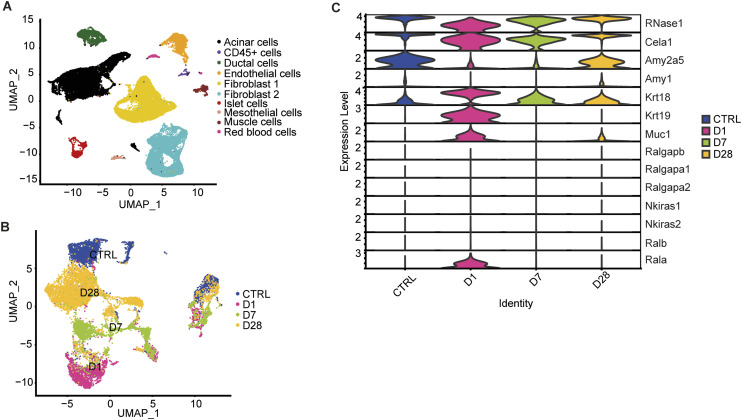
scRNA-seq data of primary acinar cells during the regeneration process. Clustering of the scRNA-seq data (GSE181276, Del Poggetto et al, 2021) was performed on murine pancreatic cells collected at day 1 (D1), day 7 (D7), and day 28 (D28) after acute pancreatitis induction with cerulein (8 h injections for 2 d) along with an unchallenged control group (CTRL). **(A)** UMAP of combined dataset (n = 33,681), colored by cell types. **(B)** UMAP plot of the acinar and ductal cell subsets after reclustering, colored by different time points. **(C)** Violin plot showing the expression of acinar (RNase1, Cela1, Amy2a5, Amy1) and ductal markers (Krt18, Krt19, Muc1) and RalGAP/Ral signaling components in the acinar compartment across the different time points.

With respect to Ral isoform involvement, we find RalA localizing to the basal body of primary cilia, colocalizing with γ-tubulin upon starvation of MEFs, whereas RalB was found at vesicular structures in the cytoplasm (Figs 6H and S6L), which might indicate that primary cilium formation is specifically controlled by the RalA isoform. RalA localization to the primary cilium was not altered upon loss of RalGAPβ or κB-Ras1/2 (Fig S6M), suggesting that the RalGAP:κB-Ras complex likely controls activity and not subcellular localization of RalA. In this context, it is interesting to note that an increase of RalA transcripts is detectable in scRNA-seq data of primary acinar cells during the regeneration process, whereas RalB expression remains unaltered (GSE181276 [Del Poggetto et al, 2021]) (Fig S7A–C). mRNA levels of RalGAP complex components are unchanged in acinar cells during regeneration, suggesting that regulation of GAP activity is not mediated via transcriptional control in this context.

### RalGAPβ deficiency promotes KRas^G12D^-initiated pancreatic cancer development

**Figure S8.**
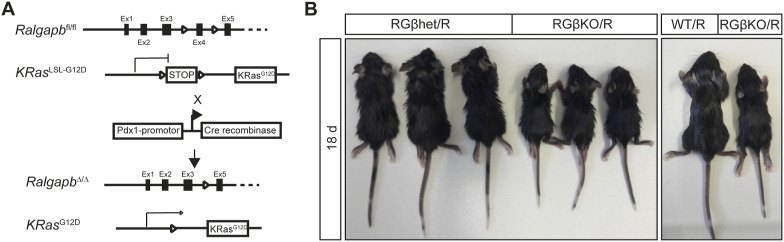
RalGAPβ deficiency promotes KRas^G12D^-initiated pancreatic cancer development. **(A)** Schematic of conditional deletion approach of exon 4 of the RalGAPβ allele and conditional expression of KRas^G12D^. **(B)** Pictures of 2 litters of RGβhet/R, RGβKO/R, and WT/R mice (18 d of age).

**Figure 7.**
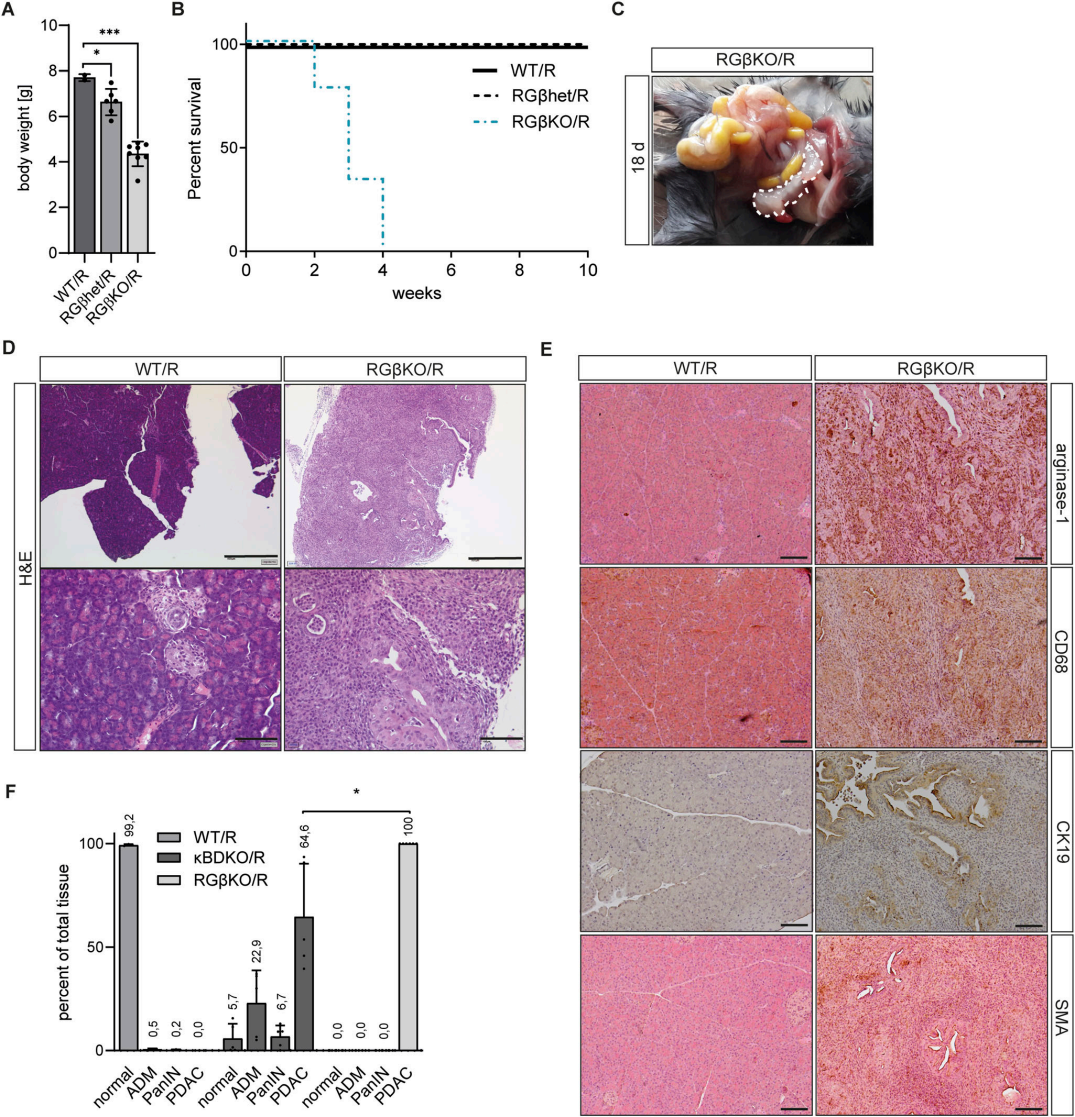
RalGAPβ deficiency promotes KRas^G12D^-initiated pancreatic cancer development. **(A)** Bodyweight of WT/R, RGβhet/R, and RGβKO/R mice (18–21 d of age). Given is mean with SDs. *t* test: WT/R:RGβhet/R: *P = 0.0484, WT/R:RGβKO/R: ***P < 0.001. **(B)** Percent survival of WT/R, RGβhet/R, and RGβKO/R mice. n = 9 animals per group. **(C)** Exemplary picture of pancreas with prominent tumor (dashed line) of an RGβKO/R animal at necropsy (18 d old). **(D)** H&E-stained pancreas tissue sections of WT/R and RGβKO/R mice (18–20 d old). Top scale bar = 500 μm, bottom scale bar = 100 μm. **(E)** Pancreas sections of WT/R and RGβKO/R animals (18–20 d old) stained for arginase-1, CD68, CK19, and SMA. Scale bar = 200 μm. **(F)** Quantification of the tissue area occupied by normal tissue, acinar-to-ductal metaplasia, PanIN lesions, and pancreatic ductal adenocarcinoma (PDAC) in WT/R (n = 3), κBDKO/R (n = 5), and RGβKO/R (n = 6) (18–28 d old) PAS-stained pancreas sections. Given is mean with SD and individual values. *t* test with Welch’s correction in case of unequal SDs: κBDKO/R (PDAC):RGβKO/R (PDAC): *P = 0.036.

We had previously identified that κB-Ras loss promoted KRas^G12D^-driven pancreatic cancer development in a genetically modified mouse model, which we attributed to loss of RalGAP complex function. Having identified that RalGAPβ deficiency has additional cellular effects in comparison with κB-Ras loss, we wanted to understand how this would affect tumor development triggered by KRas mutation. We crossed RGβKO mice with animals carrying the LSL-*KRas*^G12D^ allele (Fig S8A), which allows for conditional expression of the oncogenic KRas^G12D^ mutant protein (Hingorani et al, 2003). KRas^G12D^ expression in the pancreas triggers slow development of PanINs in mice, which progress to invasive PDAC only rarely and with advanced age (Hingorani et al, 2003). Mice that lacked RalGAPβ and simultaneously expressed the KRas^G12D^ mutant in the pancreas (*Ralgapb*^fl/fl^ *KRas*^WT/LSL-G12D^ Pdx1-Cre^+^; RGβKO/R) were visibly smaller than their WT (*KRas*^WT/LSL-G12D^ Pdx1-Cre^+^; WT/R) and RalGAPβ heterozygous knockout (*Ralgapb*^fl/+^ *KRas*^WT/LSL-G12D^ Pdx1-Cre^+^; RGβhet/R) littermates (Figs 7A and S8B) and quickly became moribund with a median survival of 21 d (Fig 7B). Upon necropsy, pancreatic tumors could readily be observed (Fig 7C), but no metastases were detected on liver or lung. Histological analyses confirmed strong pancreatic tumor development with a complete loss of normal pancreatic tissue architecture in RGβKO/R animals (Fig 7D). Tumor cells showed duct cell characteristic CK19-positive staining (Fig 7E). Tumor development was accompanied by significant fibrosis and infiltration with CD68-positive immune cells (Fig 7E). The large number of detected arginase-1–positive cell hints that most of these cells are likely M2-polarized, protumorigenic macrophages. Pancreatic tissue of only KRas^G12D^-expressing mice (WT/R) was as expected largely indistinguishable from WT tissue with only occasional lesions observable at this young age (Fig 7D). Although κB-Ras deficiency also promoted tumorigenesis, the percentage of tissue occupied by ADM/PanIN/PDAC in κBDKO/R mice (*NKIRAS1*^−/−^ *NKIRAS2*^fl/−^ *KRas*^WT/LSL-G12D^ Pdx1-Cre^+^) was lower than in RGβKO/R animals (Fig 7F). We thus hypothesize that both mechanisms — deregulation of the secretory pathway and defective primary cilium formation — in RGβKO mice are relevant to promote the tumor phenotype. As the RGβKO/R mice are smaller than their littermates and tumor development occurs so early, we cannot exclude that effects on pancreatogenesis, for example, branching morphogenesis or the formation of a ramified ductal tree, might be causal or contribute to tumor development. As these processes might be different from those in adult tissue, further analyses will be required to characterize the impact of RalGAPβ loss in the fully formed pancreas.

In summary, our data suggest that KRas mutant–driven activation of the Ral pathway is under constant repression through RalGAP complexes. Down-regulation of RalGAP as observed in human patient samples (Cao et al, 2021) might thus confer a state with heightened sensitivity to oncogenic stimulation and contribute to tumor development.

## Discussion

Here, we demonstrate that RalGAPβ deletion in vivo affects exocytosis, cell polarity, cell–cell contacts, and primary cilium formation. We suggest that in the RalGAPβ-deficient pancreas, enhanced and misrouted secretion triggers tissue damage and inflammation, whereas defective primary cilium formation prevents subsequent acinar regeneration. As a result, reduction of RalGAPβ expression alone is sufficient to induce pancreatitis and neoplasia, demonstrating the importance of this understudied Ras effector pathway in pancreatic pathology. In agreement, RalGAPβ loss dramatically accelerates tumor development when combined with mutant KRas^G12D^ expression. We determine that primary cilium formation is dependent on κB-Ras proteins, whereas control of the secretory pathway through RalGAP is not, which suggests that κB-Ras proteins control only a subset of RalGAP functions.

With our results, we provide evidence for a tumor-suppressive function of RalGAP complexes in the pancreas in vivo. To date, the role of Ral GTPase signaling in the context of pancreatic cancer had only been addressed in tumor cell line studies (Lim et al, 2006) where knockout of RalGAPβ led to enhanced migration and invasion in vitro, enhanced growth upon subcutaneous grafting in vivo, and promotion of primary growth and metastasis when PDAC cells were injected in the spleens of nude mice (Yoshimachi et al, 2021). Our current study addresses the in vivo effects of RalGAPβ loss in the pancreas in genetically modified mouse models, which allows us to study their impact on physiological functions, as well as tissue inflammation and (early) tumor development. When combined with oncogenic KRas^G12D^ mutant expression, RalGAPβ deletion promoted tumor development, resulting in fully invasive PDAC at an age of 3 wk in all animals examined. A similarly striking effect on PDAC onset has been observed upon homozygous PTEN loss in combination with KRas^G12D^ expression (*KRas*^WT/LSL-G12D^ *Pten*^fl/fl^ Pdx1-Cre^+^) (Hill et al, 2010). These animals also become moribund before weaning, presenting with exocrine atrophy, ADM, and neoplasia. Also, PTEN deletion alone in the developing pancreas leads to progressive replacement of acinar tissue and the development of proliferative ductal structures, with centroacinar cells being reported as the cell of origin (Stanger et al, 2005). Thus, both Ras downstream pathways, Ral and PI3K signaling, represent important brakes counteracting KRas^G12D^-driven neoplasia development.

As our study lacks an analysis of pancreas development, we cannot exclude effects of combined RalGAPβ loss and KRas mutation on pancreatogenesis that might be causal for or contribute to the early PDAC development. These events could be different from processes required for tumorigenesis in the adult pancreas, or adult pancreatic cells could be refractory to these changes as has been observed for KRas mutation, which can trigger PanIN and PDAC development when expressed in embryonic stages in acinar/centroacinar cells, but remains without effect when expressed in adult cells (Guerra et al, 2007). An analysis of pancreas development in RGβKO/R mice and/or deletion of RalGAPβ in the adult pancreas will be required to clearly delineate whether and how the phenotype we observed mechanistically relates to tumor formation in a fully formed pancreas. We did not detect metastases in RGβKO/R animals despite the locally invasive PDAC development, although a role of Ral GTPases in metastasis of human PDAC cell lines has been suggested previously (Yoshimachi et al, 2021; Cao et al, 2023). We hypothesize that the very fast primary tumor development and death of the animals might prevent metastasis formation simply by not allowing enough time for additionally required processes such as epithelial–mesenchymal transition to occur. It is, however, conceivable that in fully transformed human PDAC cell lines, effects of Ral signaling on migration would also be reflected in increased metastasis formation.

How κB-Ras proteins promote RalGAP activity in cells remains elusive to date. Based on our current results, different scenarios could explain the distinct phenotypes of RGβKO(/R) and κB-Ras DKO(/R) animals. Enhancement and loss of polarization of exocytosis could require a certain threshold level of Ral activity that is only obtained by loss of the complex subunit RalGAPβ, but not the RalGAP “regulator” κB-Ras, as the latter results in a much weaker increase in Ral-GTP levels in the pancreas. However, a similar increase in Ral-GTP levels is observed in κB-Ras DKO and RGβKO MEFs despite comparable deletion efficiencies in the two systems. We therefore suggest that the total cellular Ral-GTP level reflects the sum of subsets of Ral signaling events. Some of these are controlled by κB-Ras GTPases, and some are not, and the subsets contribute differentially to total Ral-GTP levels in a cell type–specific fashion. We hypothesize that κB-Ras GTPases are required for primary cilium formation during acinar regeneration, whereas RalGAPβ function is broadly required for control of Ral activity, for example, to regulate exocytosis. Such a scenario would explain the massive increase of Ral-GTP levels upon RalGAPβ loss in acinar cells, a cell type that is specialized in exocytosis. In this context, κB-Ras proteins could be involved in the localization of RalGAP complexes to regulate Ral activity at specific cellular sites, such as the basal body/primary cilium. The establishment of better tools to visualize RalGAP complex components will be required to address the role of κB-Ras.

Control of zymogen granule exocytosis by Ral GTPases in pancreatic acinar cells has to our knowledge not been investigated, but does not come as a surprise based on the established connection of Ral GTPases to exocyst functions (Shirakawa & Horiuchi, 2015). However, the exocyst has mostly been connected to delivery of basolateral and secretory proteins (Lipschutz et al, 2003; Niedziółka et al, 2024). Here, we find that enhanced Ral-GTP levels result in increased production of an apical membrane protein and its misdelivery to basolateral membranes. It will be interesting to further investigate how exactly enhanced Ral-GTP levels alter exocyst component localization and/or exocyst assembly in RGβKO cells. The same holds true for the observed effects on cell–cell contacts in RalGAPβ-deficient acinar tissue. Gap junctions are required for concerted secretory action of acinar cells in the acinus structure (Chanson et al, 1998). It is possible that RalGAPβ deficiency leads to aberrant assembly of connexons (hemi channels), to problems in connexon trafficking through the Golgi stacks, or to inappropriate insertion into plasma membranes so that efficient formation of gap junctions and aggregation into plaques are prevented. Gap junctions are required for secretion induced by Ca^2+^-mobilizing secretagogues, but down-regulated during its course (Chanson et al, 1991, 1998; Bosco et al, 1994; Yule et al, 1996). Artificially generated absence of gap junctions results in higher basal secretion, whereas induced secretion remains unaltered or reduced (Chanson et al, 1998). This suggests that enhanced secretion upon uncoupling of acinar cells might represent a means to uphold secretion during maximal stimulation. In addition, loss of connexin-32 expression in mice leads to increased severity of pancreatitis and persistent tissue damage (Frossard et al, 2003). We thus expect that the loss of gap junctions in RGβKO acinar cells contributes to the observed enhanced serum amylase levels. However, it remains to be determined whether fewer gap junctions are an initial cause or a secondary effect of enhanced secretion. Based on the fact that κB-Ras1/2 double knockout animals have normal gap junctions but defective acinar regeneration, we assume that the defective regeneration of animals with κB-Ras or RalGAPβ loss is not directly or at least not exclusively connected to loss of cellular communication.

With our study, we report for the first time an involvement of Ral GTPase signaling in the formation of primary cilia. The exocyst has been shown to be required for primary cilium assembly and maintenance by tethering vesicles to the ciliary base, delivering both plasma membrane and soluble proteins (Rogers et al, 2004; Zuo et al, 2009, 2019; Babbey et al, 2010; Das & Guo, 2011; Fogelgren et al, 2011; Niedziółka et al, 2024), and for stimulus-induced primary cilium recycling after its internalization (Rivera-Molina et al, 2021). Our data demonstrate that primary cilium formation is disturbed in RalGAPβ-, RalGAPα1/2-, and κB-Ras1/2–deficient cells. We suggest that this effect is mediated by an inability to control RalA activity, which we detect localized to the centrosome/basal body in starved cells. Interestingly, a primary cilium defect was also described for KRas^G12D^-expressing ductal cysts (Bangs et al, 2020) and PanINs are devoid of primary cilia in *KRas*^WT/LSL-G12D^ Pdx1-Cre^+^ mice (Seeley et al, 2009). Furthermore, KRas knockdown in a human PDAC cell line increased cilium formation (Kobayashi et al, 2017). This effect of KRas signaling has been ascribed to downstream activation of PI3K/AKT, MEK/ERK, and Aurora kinase A (Seeley et al, 2009; Deng et al, 2018; Kiseleva et al, 2023). Based on our results, it is conceivable that KRas signaling also uses the Ral pathway for control over primary cilia. In this context, Ral activation could be triggered directly by KRas via RalGEFs such as RalGDS or indirectly through phosphorylation of RalGAPs by Akt kinases or Aurora kinase A (Lim et al, 2010). Defective cilium formation is common to RalGAPβ and κB-Ras deficiency, and primary cilium formation is required for acinar regeneration, which is impaired in both mouse models. We suggest this as the underlying mechanism for the lack in acinar plasticity. In line, primary cilium loss, generated through deletion of genes essential for cilium formation, in the pancreas results in a similar phenotype of ADM, lipomatosis, and inflammation (Cano et al, 2006; Augereau et al, 2016). Interestingly, one study suggests that defective primary cilium formation in ductal cells is responsible for this mainly acinar cell phenotype (Augereau et al, 2016). Indeed, based on our data we cannot exclude that defective primary cilium formation in ductal cells contributes to the pancreatitis phenotype of RGβKO animals. Regarding a role of Ral GTPases in primary cilium formation, it might also be interesting to revisit that RalGAPα1 was originally identified as a brain-expressed candidate gene for abnormal brain development and neurological deficits (Schwarzbraun et al, 2004), to which primary cilia are also closely connected. Indeed, biallelic variants of RalGAPα1 have been reported in children with neurological deficits, epilepsy, and muscular hypotonia (Wagner et al, 2020), symptoms that are reminiscent of the neurological manifestation of ciliopathies, a group of developmental and degenerative disorders related to primary cilium dysfunction (Hildebrandt et al, 2011).

## Materials and Methods

### Mice

*RalGAPb*^fl/fl^ mice (Skorobogatko et al, 2018) were generously provided by Dr. A Saltiel, University of California, and crossed with Pdx1-Cre (Gannon et al, 2000) and *KRas*^LSL-G12D^ (Hingorani et al, 2003) mice (all from C57BL/6 strain background). All mice were housed in individually vented cages (IVC) containing nesting material. Constant ambient temperature (22°C ± 2°C), constant humidity (55% ± 10%), and a 12-h light/12-h dark cycle were provided. The animals were generally group-housed unless males showed aggressive behavior toward cage mates. Both sexes were used for experiments, and littermates of the same sex were randomly assigned to experimental groups. The age of animals in specific experiments is provided in the respective figure legend or the method details. Acute pancreatitis was induced by the administration of 7 h intraperitoneal injections of cerulein (HY-A0190; 100 ng/g bodyweight in 0.9% saline; MedChemExpress) after a fasting period of 12 h (water ad libitum). Feeding was resumed after the last injection. Control animals received injections with 0.9% saline. Pancreata were analyzed 8, 24 h, 10 , and 21 d after the first injection. ADM scoring was performed in a blinded fashion. Animals not involved in cerulein experiments received food ad libitum. All animal experiments were approved by the local animal use and care committee (LANUV) and the office of animal welfare of the University Clinic Münster.

### Cell lines

HEK293FT (RRID:CVCL_6911; Sex: Female) cells were obtained from Thermo Fisher Scientific (R70007). Mouse embryonic fibroblasts (MEFs) were generated from a C57BL/6 mouse and κB-Ras double knockout (DKO) MEFs from a *NKIRAS1*^−/−^ *NKIRAS2*^−/−^ embryo at day E12.5 and SV40-immortalized (Oeckinghaus et al, 2014). CRISPR/Cas9-mediated knockout of RalGAPβ, RalGAPα1, and RalGAPα2 was performed as described below to generate the MEF cell lines RGβKO, RGα1KO, and RGα1/2 DKO. shRNA-mediated knockdown of RalGAPβ (RGβ) in WT MEFs and that of RalA/RalB in WT, RGβKO, and κBDKO MEFs (shRalA/B #4) were achieved via lentiviral transduction as described below. Knockdown cells were kept under constant selection pressure (1.5 μg/ml puromycin). All cell lines were cultured in DMEM high glucose (D6546; Sigma-Aldrich) supplemented with 10% FBS (S1810; Biowest), 10 mM L-glutamine (G7513; Sigma-Aldrich), and 50 U penicillin–streptomycin (15070063; Thermo Fisher Scientific) at 37°C with 5% CO_2_.

### Bacterial strains

*Escherichia coli* DH5α or Stbl3 (for lentiviral plasmids) was used for plasmid amplification and *E. coli* BL21(DE3) for the expression of recombinant proteins. Both strains were made competent by chemical treatment and transformed by heat shock at 42°C using standard protocols.

### Plasmids

GST-tagged rat Sec5 was expressed from a modified pCDF vector with a GST-PreScission tag. shRNA targeting sequences were chosen using the InvivoGen siRNA wizard tool (https://www.invivogen.com/sirnawizard) and shRNA oligonucleotides designed with overhangs compatible with AgeI and EcoRI restriction sites. The forward and reverse primers (each 10 μM f.c.) were annealed at 95°C for 5 min with NEB Buffer 2.1 (B7202; New England Biolabs). The annealed product was ligated (T4 DNA Ligase, M0202S; New England Biolabs) into an AgeI-HF (R3552S; New England Biolabs)– and EcoRI-HF (R3101S; New England Biolabs)–digested pLKO.1 puro plasmid (gift from Bob Weinberg; Addgene plasmid # 8453 [Stewart et al, 2003]). For further instructions, visit the Addgene protocol for pLKO.1 cloning and lentiviral production: https://www.addgene.org/protocols/plko. Used oligonucleotides are listed in Table S4. For CRISPR knockouts, sgRNA sequences were chosen with the CRISPick tool (Doench et al, 2016; Sanson et al, 2018) (https://portals.broadinstitute.org/gppx/crispick/public), designed with BbsI-compatible overhangs, and annealed as described above. The annealed product was ligated into BbsI (ER1011; Thermo Fisher Scientific)-digested px458 or px459 plasmids (gifts from Feng Zhang; Addgene plasmids # 48138 and # 62988 [Ran et al, 2013]).

Table S4. shRNA oligonucleotide sequences.

### CRISPR/Cas9-based generation of RalGAP knockout MEFs

SV40-immortalized WT MEFs were simultaneously transfected with px458 and px459 plasmids containing gRNAs targeting upstream (RGα1sg1 cagcacggagagacccacgt) and downstream (RGα1sg2 tgtgtgctccttgtaaacag) of the ATG-containing exon 1 of RGα1 to achieve complete exon deletion. Transfection was performed using the jetPRIME DNA and siRNA transfection reagent (101000046; Polyplus) as specified by the manufacturer. 24 h after transfection, GFP-positive cells were FACS-sorted and returned to culture to recover overnight. Cells were then selected for 36 h with 2 μg/ml puromycin. When cells started to return to normal growth, single-cell clones were obtained by limited dilution. Successful deletion of RGα1 was tested using PCR screening of gDNA samples with primers targeting outside of the deleted region (mRGα1 screen 2F aagactacgggttccactgg, mRGα1 screen 2R tgctggagatctggtgctag) and confirmed by qRT–PCR using primers targeting inside the deleted exon (mRGα1RTEx1_F3 gacgtgaagaagtccaccc, mRGα1RTEx1_R3 ggtcaatagattctgcattctcg) and immunoblot. RGα1/2 DKO MEFs were generated as described above using px458 and px459 vectors containing gRNAs targeting upstream (RGα2sg1 tcaggcacagaggttacccg) and downstream (RGα2sg2 gcggaccagaggcacccgtg) of the ATG-containing exon 1 of RGα2. These plasmids were transfected into a clonal RGα1 knockout cell line generated previously. Successful deletion of RGα2 was tested using PCR screening (mRGα2 screen 4F gagtgagagatggtagttcaacg, mRGα2 screen 4R ggaggtcactgcgcagactcgaagc) and confirmed by qRT–PCR using primers targeting inside the deleted exon (mRGα2RTEx1_F gaaggagccacggagatgt, mRGα2RTEx1_R catccacgttatccagcagc) and immunoblot. RGβKO MEFs were generated using gRNAs targeting upstream (RGβsg1 agaagcagtagtggtagtgt) and downstream (RGβsg2: gctgctaactccagttgcag, for RGβKO #1 or RGβsg3: agagagtgttgggcgagagg for RGβKO #2) of exon 2 of RalGAPβ. Successful deletion of RGβ was tested using PCR screening (mRGβ screen 2F tgaaagggaaatgtcggaaa, mRGβ screen 2R tgagttcctgccttggtttt) and confirmed by qRT–PCR using primers targeting inside the deleted exon (mRGβRTEx2_F cagtggctggtagtgagagt, mRGβRTEx2_R gcaacaccaaagccataatcc) and immunoblot. All oligonucleotides used are listed in Table S5.

Table S5. CRISPR/Cas9-related oligonucleotides and cloning primers.

### Lentiviral transduction

For lentiviral transductions, HEK293FT cells were seeded at 4 × 10^6^ cells per 10-cm plate. The next day, the cells were serum-starved for 1 h before transfection with polyethyleneimine (PEI, 43896; Alfa Aesar). For this, 6 μg of pLKO.1 plasmid, 5.4 μg of packing plasmid psPAX2 (gift from Didier Trono; Addgene plasmid # 12260), and 0.6 μg of envelope plasmid pCMV-VSV-G (gift from Bob Weinberg; Addgene plasmid # 8454 [Stewart et al, 2003]) were diluted in 200 μl of PBS, mixed with 48 μl of PEI (1 mg/ml stock in PBS), and incubated for 15 min at RT before dropwise addition to the cells. Medium was changed to full growth medium 3–5 h after transfection. A pLKO.1 plasmid with a shRNA sequence targeting GFP was used as a control. Virus was harvested 48 h after transfection and either to use in vitro ADM assays (see below) or to infect MEFs that were seeded the previous day at 1 × 10^5^ cells per 10-cm dish. Antibiotic selection with 2 μg/ml puromycin was started 48 h after infection of MEFs to achieve stable expression.

### Transfection of MEFs

N-terminally ALFA-tagged RalGAPβ or RalGAPα2 was cloned into a modified pITR-TTP vector (Fitzian et al, 2021) via MluI and NotI restriction sites. κBDKO MEFs were transfected with pITR-ALFA-RalGAPβ, pITR-ALFA-RalGAPα2, and the transposase-expressing pCMV-Trp plasmid (9:1 ratio) using jetPRIME DNA and siRNA transfection reagent (101000046; Polyplus) for stable reconstitution. In the same way, codon-optimized KRas^G12D^ was cloned into a modified pITR-TTP vector via MluI and NotI restriction sites and transfected into WT and RGβKO MEFs. Oligonucleotides used are listed in Table S5. Cells were selected for 48 h with 2 μg/ml puromycin and maintained with 1.5 μg/ml puromycin afterward. To confirm exogenous expression, proteins were isolated with Bäuerle lysis buffer (20 mM Tris, pH 8, 350 mM NaCl, 20% glycerin, 1 mM MgCl_2_, 0.5 mM EDTA, 0.1 mM EGTA, 1% NP-40 supplemented with 1 mM DTT, Protease and Phosphatase Inhibitor Cocktail [A32965, A32957; Thermo Fisher Scientific]) and analyzed by immunoblot.

### In vitro ADM assay

The protocol was modified from a previously published procedure (Fleming Martinez & Storz, 2019). Pancreata from mice were dissected, washed twice in 10 ml Hank’s Buffered Salt Solution (HBSS, H6648; Sigma-Aldrich) with 50 U penicillin–streptomycin (15070063; Thermo Fisher Scientific), and cut into small pieces (∼2 mm) in fresh 10 ml HBSS with penicillin–streptomycin. After centrifugation (300*g*, 2 min, 4°C), the supernatant including fat was discarded and digestion performed with sterile-filtered 5 ml Collagenase, Type I (2 mg/ml in HBSS; 17018029; Thermo Fisher Scientific), with shaking at 220 rpm for 20 min at 37°C (Innova43 incubator shaker series, New Brunswick Scientific). With no large pieces remaining, the dissociation was stopped by placing on ice and addition of cold 5 ml HBSS + 5% FBS (S1810; Biowest) and centrifugation (300*g*, 2 min, 4°C). Acini were washed twice with cold 10 ml HBSS + 5% FBS via centrifugation and then resuspended in cold 5 ml HBSS + 5% FBS. The acinus suspension was consecutively filtered through 500-μm and 100-μm cell strainers, and filters were washed with cold 5 ml HBSS + 5% FBS. The acini were layered on top of 20 ml HBSS + 30% FBS and centrifuged (233*g*, 2 min, 4°C). The pellet was resuspended in Waymouth’s medium (31220072; Thermo Fisher Scientific) with 1% FBS, 0.1 mg/ml Soybean Trypsin Inhibitor (17075029; Thermo Fisher Scientific), and 1 μg/ml dexamethasone (D4902; Sigma-Aldrich). Volume was adjusted depending on acinus density to reach a thin acinus suspension for optimal quantification. 300 μl of Cultrex Basement Membrane Extract (BME), PathClear (3432-005-01; R&D Systems), was put in a 24-well plate and dried for 1 h at 37°C with 5% CO_2_. The acinus suspension was mixed 2:1 (v:v) with Cultrex BME on ice using 300 μl acinus suspension and 150 μl Cultrex BME and layered on top of the solidified BME layer. After 1 h of incubation at 37°C with 5% CO_2_, 1 ml of warm Waymouth’s medium, supplemented as above, was added. For inhibitor studies, 50 μM or 70 μM (f.c.) amlexanox (HY-B0713; MedChemExpress) in DMSO was dissolved in BME before mixing with the cell suspension and dissolved in the covering medium before medium changes. Medium (with or without fresh inhibitors) was changed after 24 and 72 h and the assay quantified on day 5. Seven fields per view per condition were evaluated. ADM events, defined as clear formation of a ring-like structure by an embedded acinus, and total acinus numbers were counted and percentage of ADM events determined for all conditions. The mean of the percentage of ADM events of the control condition was set as 100%, and all other values were calculated and normalized to the mean. For lentiviral transduction, virus produced in HEK293FT cells was harvested 48 h after transfection and filtered through a 0.22-μm filter. 2 ml of undiluted acinus suspension was added to 4 ml of lentivirus-containing medium with 6 μg/ml (f.c.) polybrene (H9268; Sigma-Aldrich). The acini were incubated for 1 h at 37°C with 5% CO_2_ with gentle swirling every 15 min followed by an additional 4-h incubation without movement. Afterward, the acini were embedded as described above. The remaining cell suspension was used for knockdown efficiency analysis by qRT-PCR after 3 d in suspension culture plates.

For assessment of viability, the medium was collected and the Cultrex BME plugs were scraped with 1.5 ml Cell Recovery Solution (254253; Corning) in three steps into the collected medium and incubated on ice for 75 min. The isolated cells were stained with 0.4% trypan blue solution and counted in a Neubauer chamber.

### Amylase activity assay

Peripheral blood was obtained from femoral blood vessels after termination (∼50 μl) and incubated on ice for 30 min. Serum was then separated by two centrifugation steps (800*g*, 10 min, 4°C and 9,300*g*, 2 min, 4°C) and flash-frozen in liquid nitrogen. Serum amylase levels were determined using a colorimetric Amylase Activity Assay Kit (MAK009; Sigma-Aldrich) according to the manufacturer’s protocol and analyzed on a microplate photometer (51119000; Multiskan FC; Thermo Fisher Scientific).

### GST-Sec5 pulldown

GST-tagged rat Sec5 (aa 1–99) was recombinantly expressed in *E. coli* BL21DE3 and bound to glutathione Sepharose 4B (GE17-0756-01; Merck). Washed beads were run on SDS–PAGE to estimate the amount of bound GST-Sec5 in relation to a standard BSA curve. 20 μg GST-Sec5 was used per pulldown reaction.

MEFs were lysed in Ral lysis buffer (50 mM Tris, pH 7.5, 100 mM NaCl, 4 mM MgCl_2_, 2 mM EGTA, and 1% Triton X-100 supplemented with 1 mM DTT, Pierce Protease Inhibitor Cocktail [A32965; Thermo Fisher Scientific], and Pierce Phosphatase Inhibitor Cocktail [A32957; Thermo Fisher Scientific]). Tissue samples (∼5–10 mm^3^) were lysed in Ral lysis buffer using a stick homogenizer. After 10-min incubation rotating at 4°C, lysates were cleared by centrifugation (16,100*g*, 10 min, 4°C) and protein concentration was determined with Bradford Reagent (B6916; Sigma-Aldrich). Adjusted lysates were incubated with glutathione sepharose containing 20 μg of GST-Sec5 for 25-min rotating at 4°C. Beads were washed three times with 1 ml Ral lysis buffer (100*g*, 1 min, 4°C) and then analyzed by SDS–PAGE and immunoblot.

### Immunoblotting

Proteins from MEFs were isolated with Bäuerle lysis buffer (20 mM Tris, pH 8, 350 mM NaCl, 20% glycerin, 1 mM MgCl_2_, 0.5 mM EDTA, 0.1 mM EGTA, 1% NP-40 supplemented with 1 mM DTT, Pierce Protease Inhibitor Cocktail (A32965; Thermo Fisher Scientific), and Pierce Phosphatase Inhibitor Cocktail (A32957; Thermo Fisher Scientific)). After 10-min incubation rotating at 4°C, lysates were cleared by centrifugation (16,100*g*, 10 min, 4°C) and protein concentration was determined with Bradford Reagent (B6916; Sigma-Aldrich). Proteins separated by SDS–PAGE (sodium dodecyl sulfate–polyacrylamide gel electrophoresis) were blotted on an Immobilon-FL (IPFL00010; Millipore) membrane using a semidry system (Trans-Blot Turbo Transfer System, 1704150; Bio-Rad). For imaging on the Odyssey CLx Imaging System (LI-COR), membranes were blocked for 1 h in blocking buffer (0.1% casein [E0789; Sigma-Aldrich] in 0.2 × PBS). Primary antibodies were diluted 1:1,000 in 1:1 PBS:blocking buffer with 0.1% Tween and incubated with the membranes at 4°C overnight. Secondary antibodies were applied 1:4,000 for 1 h in 1:1 PBS:blocking buffer with 0.1% Tween and 0.01% SDS: IRDye 800CW donkey anti-mouse (926-32212; LI-COR) or IRDye 680RD donkey anti-rabbit (926-68073; LI-COR). If desired for normalization, the Revert 700 Total Protein Stain Kit (926-11016; LI-COR) was used before blocking following the manufacturer’s instructions.

For chemiluminescent imaging, membranes were blocked for 1 h in 3% BSA (11930; SERVA Electrophoresis) in TBS-T (1% Tween). Primary antibodies were diluted 1:1,000 in 1.5% BSA/TBS-T and incubated with the membranes at 4°C overnight. Secondary antibodies were applied 1:4,000 for 1 h in 1% dry milk powder (T145.2; Carl Roth) in TBS-T. Secondary antibodies used were horseradish peroxidase goat anti-mouse (115-035-044; Jackson ImmunoResearch) or horseradish peroxidase goat anti-rabbit (111-035-045; Jackson ImmunoResearch).

The following primary antibodies were used: anti-RalGAPβ (Gridley et al, 2006) (provided by Dr. Gus Lienhard, Geisel School of Medicine, Dartmouth or Proteintech, 28330-1-AP), anti-RalA (610221; BD Bioscience or 13629-1-AP; Proteintech), anti-RalB (VMA00168; Bio-Rad or 12340-1-AP; Proteintech), anti-GAPDH (60004-l-lg; Proteintech), anti-β-tubulin (10094-1-AP; Proteintech), anti-RALGAPα1 (HPA000851; Sigma-Aldrich), anti-RALGAPα2 (29843-1-AP; Proteintech), anti-pAkt (4060; Cell Signaling Technologies), anti-Akt (9272; Cell Signaling Technologies), anti-pErk (9101; Cell Signaling Technologies), anti-Erk (4695; Cell Signaling Technologies), anti-pPAK1/2 (85044-1-RR; Proteintech), anti-PAK1 (21401-1-AP; Proteintech).

### FACS

Pancreata were resected, washed in PBS, and minced into small pieces (1–2 mm) in 2 ml RPMI-1640 without phenol red (R7509; Sigma-Aldrich) supplemented with 2% FBS (S1810; Biowest), 10 mM L-glutamine (G7513; Sigma-Aldrich), and 50 U penicillin–streptomycin (15070063; Thermo Fisher Scientific). After two washing steps with 5 ml RPMI/2% FBS, each minced pancreas was digested in 2 mg/ml Collagenase, Type 4 (LS004188; Worthington-Biochemical), 2 mg/ml Dispase II (D4693; Sigma-Aldrich), and 20 μg/ml DNase I (A3778; AppliChem) dissolved in 15 ml HBSS (H6648; Sigma-Aldrich) at 37°C with 5% CO_2_ for 1 h, including thorough pipetting every 15 min to enhance dissociation. The isolated cells were then washed two times with 35 ml PBS (400*g*, 5 min, 4°C). The cells were resuspended in 0.5 ml RPMI/2% FBS and filtered through a 35-μm cell strainer, the filter was washed with 0.5 ml RPMI/2% FBS, and 0.1 mg/ml Soybean Trypsin Inhibitor (17075029; Thermo Fisher Scientific) was added. For acinar cell sorting, the cells were stained 1:25 with rat MPx1 MICO-2A6 (gift from C Dorrell [Dorrell et al, 2011]) for 20 min on ice, then washed three times with 3 ml RPMI/2% FBS (400*g*, 5 min, 4°C), and stained 1:200 with FITC goat anti-rat (554016; BioLegend) for 10 min on ice. After a washing step with 3 ml RPMI/2% FBS (400*g*, 5 min, 4°C), the cells were counterstained with APC rat anti-CD45 (103112; BioLegend) and PE-Cy7 rat anti-CD19 (55284, clone 1D3; BD Biosciences) for 20 min. After a final washing step, the cells were stained 1:100 with 7-AAD Viability Staining Solution (420404; BioLegend). Cell sorting was performed on a FACSAria II using the FACSDiva version 9.0.1 and FACSuite version 1.0.6 software (BD Biosciences). Flow cytometry data were analyzed using FlowJo software version 10.5.3 (BD Life Sciences).

### RNA preparation and qRT–PCR plus primer

Tissue samples (∼5 mm^3^) were flash-frozen in liquid nitrogen and pestled, and RNA was extracted using the NucleoSpin RNA Plus Mini Kit (740984; Macherey-Nagel) according to the manufacturer’s instructions. RNA from MEFs was obtained with TRIzol Reagent (15596018; Thermo Fisher Scientific), and the Quick-RNA Microprep Kit (R1051; Zymo Research) was used to isolate RNA from transduced acini. cDNA was generated using RevertAid RT Kit (K1691; Thermo Fisher Scientific) with 500–1,000 ng of RNA per reaction. Quantitative PCR was performed with Luna Universal qRT-PCR Master Mix (M3003E; New England Biolabs) on StepOnePlus Real-Time PCR System Thermal Cycling Block (Applied Biosystems, Thermo Fisher Scientific). Data were analyzed using StepOne software (version 2.3, Applied Biosystems). The used qRT-PCR primers are listed in Table S6.

Table S6. qRT-PCR primer sequences.

### RNA library preparation and sequencing analysis

For RNA library preparation, 0.5–1 × 10^6^ MPx1-positive FACS-sorted cells were used for RNA isolation using the Quick-RNA Microprep Kit (R1051; Zymo Research) according to the manufacturer’s instructions. Library preparation of the total RNA was performed with NEBNext Ultra II Directional RNA Library Prep Kit for Illumina (E7760; New England Biolabs) and NEBNext Poly(A) mRNA Magnetic Isolation Module (E7490; New England Biolabs). Single-end read sequencing was performed using NextSeq 2000 System with a read length of 72 bp. The samples were demultiplexed with Illumina DRAGEN Bio-IT Platform. Quality control was done using FastQC version 0.11.9 (Andrews, 2010). Trimmomatic version 0.39 (Bolger et al, 2014) was used for adapter and low-quality end trimming and for general quality trimming using a sliding window of 4 bp with a minimal average base quality of 15. Reads below a minimum read length of 15 bp were discarded. The resulting reads were aligned to the Ensembl GRCm39 reference genome, using HISAT2 version 2.2.1 (Kim et al, 2019), and sorted using SAMtools version 1.16.1 (Danecek et al, 2021). Gene-based read counting was done using HTSeq version 2.0.3 (Anders et al, 2015) with the Ensembl annotation version 107.

Differential expression analysis was performed using the R package DESeq2 version 1.38.3 (Love et al, 2014). The R package biomaRt version 2.54.0 (Durinck et al, 2009) was used to convert Ensembl IDs to mgi symbols and to retrieve GO terms. KEGG annotations were retrieved with msigdbr version 7.5.1 (Dolgalev, 2022). Gene set enrichment analysis was done using the R package fgsea version 1.24.0 (Korotkevich et al, 2021 *Preprint*). A significance threshold of 0.05 for the FDR-corrected *P*-values was used to determine significantly expressed genes and significant gene sets. Plots were created using the R packages gplots version 3.4.1 (Warnes et al, 2005), ggplot2 version 3.4.1 (Wickham, 2016), pcaExplorer version 2.24.0 (Marini & Binder, 2019).

For the RNA-sequencing analysis of samples from cerulein-treated animals, the same workflow was applied with slightly different software versions: Trimmomatic version 0.38, HISAT2 version 2.1.0, HTSeq version 2.0.3 with the Ensembl annotation version 92, and DESeq2 version 1.40.1. The R package org.Mm.eg.db version 3.17.0 (Carlson, 2020) was used to convert Ensembl IDs to mgi symbols. GSEA software version 4.3.2 (Mootha et al, 2003; Subramanian et al, 2005), a joint project of UC San Diego and Broad Institute, was used for the gene set enrichment analysis with the following gene ontology and KEGG MEDICUS gene sets: ftp.broadinstitute.org://pub/gsea/msigdb/human/gene_sets/c5.all.v2023.2.Hs.symbols.gmt or ftp.broadinstitute.org://pub/gsea/msigdb/human/gene_sets/c2.cp.kegg_medicus. v2023.2.Hs.symbols.gmt. Plots for RNA-sequencing analysis of samples from cerulein-treated animals were created using the R packages with slightly different software versions: gplots version 3.1.3, ggplot2 version 3.4.2, and pcaExplorer version 2.26.1. Bar graphs were created using the R package ggplot2 version 3.3.5 and heatmaps using the R package ComplexHeatmap version 2.10.0 (Gu et al, 2016). The Venn diagram was created using an online tool (J.C. Oliveros, https://bioinfogp.cnb.csic.es/tools/venny/index.html).

### Immunohistochemistry and immunofluorescence

Pancreata were fixed for 48 h in 4% PFA, incubated in 70% ethanol, embedded in paraffin, and cut into 5-μm sections. Sections were deparaffinized with xylene (534056; Honeywell), rehydrated, and stained with Mayer’s hemalum solution (1.09249; Sigma-Aldrich) and 0.5% aqueous Eosin G (7089.1; Carl Roth) (H&E) or by periodic acid–Schiff (PAS) staining.

For immunostainings, antigen retrieval was performed using 10 mM trisodium citrate (3580.3; Carl Roth), pH 6, and heat for 20 min. Endogenous peroxidases were then inactivated with 3% H_2_O_2_ in methanol for 12 min, and sections were blocked for 1 h with 3% horse serum (H1270; Sigma-Aldrich) in PBS. Staining with primary antibodies was performed in 3% horse serum in a humidified chamber overnight at 4°C. Antibodies used were as follows: anti-CK19 (1:100, sc-33111; Santa Cruz Biotechnology), anti-arginase-1 (1:100, D4E3M, 93668; Cell Signaling Technologies), anti-CD68 (1:100, 28058-1-AP; Proteintech), anti-SMA (1:100, 14395-1-AP; Proteintech), anti-pAkt substrate (9611; Cell Signaling Technologies), anti-pErk (9101; Cell Signaling Technologies). Secondary antibody incubation was performed for 1 h at RT with biotinylated horse anti-rabbit (1:250, BA-1100; Vector Laboratories) or biotinylated horse anti-goat (1:250, BA-9500; Vector Laboratories) antibodies. The sections were covered with VECTASTAIN Elite ABC-HRP reagent (VEC-PK-7100; Vector Laboratories) for 30 min, and the DAB substrate kit (VEC-SK-4100; Vector Laboratories) was used according to the manufacturer’s instructions. If desired, H&E staining was performed. Sections were then dehydrated and mounted in DePeX (18243.01; SERVA Electrophoresis). Images were taken with an Olympus CKX53 microscope.

For immunofluorescence stainings, deparaffinization, rehydration, and antigen retrieval were performed as described above. The sections were then permeabilized using 0.2% Triton X-100 in PBS for 10 min followed by blocking in 2% HISTOPRIME donkey serum (END9010; Linaris) in PBS. Staining with primary antibodies was performed in a humidified chamber overnight at 4°C. Antibodies used were as follows: anti-CK19 (1:100, 10712-1-AP; Proteintech or 1:20, TROMAIII; Developmental Studies Hybridoma Bank), anti-Cpa1 (1:100, AF2765; Novus Biologicals), anti-RalA (1:100, 13629-1-AP; Proteintech), anti-RalB (1:100, 12340-1-AP; Proteintech), anti-laminin-α1 (1:100, L9393; Sigma-Aldrich), anti-calnexin (1:100, 10427-2-AP; Proteintech), anti-GM130 (1:100, 610822; BD Biosciences), anti-connexin-32 (1:20, R5.21C; Developmental Studies Hybridoma Bank), anti-ZO-1 (1:20, R26.4C; Developmental Studies Hybridoma Bank), anti-E-cadherin (1:50, 610181; BD Biosciences), anti-β-actin (1:50, sc-47778; Santa Cruz Biotechnology), anti-acinar-1 (1:20, 3.7A12; Developmental Studies Hybridoma Bank), anti-γ-tubulin (1:50, NB500-574; Novus), anti-Arl13b (1:100, 17711-1-AP; Proteintech), anti-acetylated tubulin (1:100, 66200-1-lg; Proteintech). Slides were incubated with the following secondary antibodies diluted 1:250 for 1 h at RT: Alexa Fluor 488 donkey anti-rabbit (711-545-152; Jackson ImmunoResearch), Cy3 donkey anti-goat (705-165-147; Jackson ImmunoResearch), Alexa Fluor 647 donkey anti-rat (712-605-153; Jackson ImmunoResearch), Cy3 donkey anti-mouse (715-165-151; Jackson ImmunoResearch), Cy-3 goat anti-mouse (405309; BioLegend), Alexa Fluor 647 donkey anti-mouse (715-605-150; Jackson ImmunoResearch).

To reduce endogenous mouse Ig staining when applying mouse antibodies, the M.O.M. (Mouse on Mouse) Immunodetection Kit Basic (BMK-2202; Vector Laboratories) was used for blocking and antibody incubation. To reduce background staining, sections were quenched using TrueVIEW Autofluorescence Quenching Kit (SP-8500; Vector Laboratories) for 5 min following the manufacturer’s instructions. Slides were mounted in VECTASHIELD Vibrance Antifade Mounting Medium with DAPI (H-1800; Vector Laboratories).

For immunofluorescence microscopy of MEFs, 2 × 10^4^ cells were seeded per well of a 12-well plate on glass coverslips. The next day, cells were serum-starved for 48 h. After washing with PBS, cells were fixed with ROTI Histofix (4% formaldehyde, P087.1; Carl Roth) for 15 min. For permeabilization, the cells were incubated twice in 0.1% Tween in PBS for 5 min. Cells were blocked in 0.1% Tween with 0.1% BSA in PBS for 45 min. Primary antibodies were applied 1:200 in 0.1% Tween with 0.1% BSA in PBS at 4°C overnight. After three washing steps with 0.1% Tween in PBS, the secondary antibodies were applied 1:250 in 0.1% Tween with 0.1% BSA in PBS for 1 h at RT protected from light. After three washing steps with 0.1% Tween in PBS, coverslips were mounted in VECTASHIELD Vibrance Antifade Mounting Medium with DAPI.

Immunofluorescence stainings were analyzed on an LSM700 laser-scanning confocal microscope (ZEISS), and images were processed using ZEISS ZEN Black SP1 software (edition 2.3).

### TEM

For electron microscopy analysis, small pieces of mouse pancreas (∼1 mm^3^) were fixed in premixed 2% PFA, 2.5% glutaraldehyde in 0.1 M sodium cacodylate buffer, pH 7.4 (SCB; 15960-01; Electron Microscopy Sciences) for 2 d at 4°C. Mouse tissue was washed three times in SCB, post-fixed for 1.5 h in 1% osmium tetroxide in SCB, washed three additional times in SCB, and dehydrated in an ascending alcohol series up to 100% ethanol, followed by 100% propylene oxide. Samples were then infiltrated with EMbed 812 (Science Services) resin using propylene oxide-to-resin ratios of 2:1, 1:1, and 1:2 followed by incubation in 100% resin three times. The tissue was cured in fresh resin at 60°C for 36 h. Ultra-thin slices of 70 nm were cut and stained with 2% uranyl acetate for 20 min and 3% lead citrate for 2 min. Images were taken by Philips CM10 transmission electron microscope (Philips) equipped with a TVIPS TEMCam F416 CCD camera (Tietz Video and Image Processing Systems).

### Analysis of scRNA-seq data

The count data from Del Poggetto et al were downloaded from GEO accession GSE181276. The data were then loaded into Seurat v 4.4.0 (Hao et al, 2021) and normalized using the standard parameters. The FindVariableFeatures function was used to select 2,000 highly variable genes. The data were subsequently scaled using ScaleData. Clustering was performed on the scaled data using the first 20 principal components and the top 2,000 highly variable genes with the FindPCA function. A Uniform Manifold Approximation and Projection (UMAP) was then generated and plotted using a clustering resolution of 0.5. Cell types were annotated using specific marker genes. Violin plots were generated using the VlnPlot function in Seurat.

### Statistics and reproducibility

Information concerning statistical analyses can be found in the figure legends. Mean ± SD is given and significance calculated via a two-sided *t* test if not indicated otherwise. n represents the number of independently performed experiments or the number of individual animals studied, if not indicated otherwise in the respective figure legend. All statistical analyses were performed with GraphPad Prism (version 10.2.20).

### Quantification of acinus size and nucleus numbers

Pancreas tissue sections of 15-wk-old WT and RGβKO mice were stained for laminin-α1 and DAPI and analyzed by confocal immunofluorescence microscopy. A minimum of 15 acini per image were measured in 5–6 separate pictures (40x objective) of two individual mice per genotype using QuPath (Bankhead et al, 2017) (version: 0.4.2). The circumference of individual acinus clusters was annotated by hand according to the laminin-α1 staining. The number of nuclei per annotated acinus was counted using the cell detection tool of QuPath. Statistical analysis was performed using GraphPad Prism (version 10.2.20).

### ADM scoring and quantification of histology

H&E-stained pancreas sections were scored in a blinded fashion based on the relative tissue area affected by ADM (score 0: 0–2%; score 1: 2–15%; score 2: 15–50%; score 3: >50%). PAS-stained sections were scanned, and areas of ADM, PanINs, dilated ducts, inflammation, and lipomatosis were annotated using QuPath (Bankhead et al, 2017) (version 0.4.2).

### Quantification of primary cilia in ducts

Pancreas tissue sections of 13- to 15-wk-old WT and RGβKO old mice were stained for Arl13b, acetylated tubulin, CK19, and DAPI and analyzed by confocal immunofluorescence microscopy. 10 z-stacks per mouse (63x objective) were taken from three individual mice per genotype and converted to maximum intensity projections. A total of 43 ducts were analyzed per genotype. Ductal primary cilia were counted in the projections, according to Arl13b and acetylated tubulin costaining, and values were calculated relative to the number of nuclei in one duct, according to DAPI and CK19 staining. Statistical analysis was performed using GraphPad Prism (version 10.2.20).

### Quantification of primary cilia in MEFs

MEFs were serum-starved for 48 h, stained for Arl13b, γ-tubulin, and DAPI, and analyzed by confocal immunofluorescence microscopy. 5 z-stacks per cell line (40x objective) were taken from two–three individual experiments and converted to maximum intensity projections. Primary cilia were counted in the projections, according to Arl13b and γ-tubulin staining, and percentage values calculated relative to the total number of cells, according to DAPI staining. Statistical analysis was performed using GraphPad Prism (version 10.2.20).

## Supplementary Material

Reviewer comments

## Data Availability

RNA-sequencing data have been deposited at the functional genomics data collection ArrayExpress (EMBL-EBI) under the titles “RNA-sequencing of isolated pancreatic acinar cells from RalGAPβ knockout mice against control mice” (ArrayExpress accession E-MTAB-15029) and “RNA-sequencing of isolated pancreatic acinar cells from RalGAPβ knockout mice against control mice treated with cerulein” (ArrayExpress accession E-MTAB-15030). The article does not report the original code. Used R packages are described in the method details. All data reported in this article will be shared by the lead contact upon request. Any additional information required to reanalyze the data reported in this article is available from the lead contact upon request.
