# Supplementary material for: RalGAP complexes control secretion and primary cilia in pancreatic disease
Source: Life Sci Alliance. 2025 Jun 9;8(8):e202403123. doi: 10.26508/lsa.202403123 (PMC12149561; doi:10.26508/lsa.202403123)
Supplement: Supplementary file 1 [file LSA-2024-03123_TableS1.docx]

**Table S1 - KEGG pathway analysis of RGβKO acinar cells in comparison to WT acinar cells.** List of KEGG pathways obtained by gene set enrichment analysis after RNA sequencing from RGβKO and WT sorted acinar cells. Given are the KEGG pathway name, p-value, adjusted p-value, log2err, enrichment score (ES), normalized enrichment score (NES), gene set size and the gene names that contributed to the leading edge. Values are rounded to 4 decimal points and pathways with a p‑value <0.05 are depicted.

| **KEGG pathway** | **p-value** | **adjusted p-value** | **log2err** | **ES** | **NES** | **size** | **Leading edge** |
| --- | --- | --- | --- | --- | --- | --- | --- |
| KEGG_RIBOSOME | 0.0000 | 0.0000 | 1.7624 | 0.9146 | 3.3366 | 83 | Rps5, Rpl26, Rpl7a, Rps6, Rps17, Rps12, Rpl14, Rpl31, Rpsa, Rps26, Rpl36a, Rpl23, Rpl28, Rpl34, Rpl12, Rps20, Rps16, Rpl6, Rps8, Rpl37, Rpl10a, Rpl37a, Rps29, Rpl23a, Rpl4, Rpl19, Rpl13, Rpl35, Rps15, Rps3a1, Rpl38, Rpl22, Rps13, Rpl27a, Rps3, Rps11, Rps27a, Rpl3, Rplp0, Rpl8, Rpl15, Rps24, Rpl39, Rpl36, Rps9, Rps25, Rpl18a, Rpl7, Rps4x, Mrpl13, Rps2, Rps27l, Rps7, Rplp1, Rpl32, Rpl5, Rps15a, Rps27, Rps19, Rpl17, Rpl22l1, Rpl27, Rplp2, Rpl18, Rpl11, Rpl10, Rpl13a, Rps21, Rpl30, Fau, Rps28, Rpl9, Rpl29, Rps18 |
| KEGG_LYSOSOME | 0.0000 | 0.0000 | 0.9545 | 0.6615 | 2.5051 | 116 | Ctsl, Lamp1, Cd63, Acp5, Ctse, Ctsk, Ctsd, Hexa, Gns, Ctns, Glb1, Gba, Fuca1, Arsb, Sumf1, Ap3s1, Ctsa, Lamp2, Gga2, Nagpa, Atp6v0b, Asah1, Acp2, Ctsz, Slc17a5, Clta, Galc, Laptm4a, Atp6v0d1, Naga, Ctsc, Aga, Gnptg, Cln5, Gnptab, Atp6ap1, Man2b1, Cd164, Gga1, Ap1m2, Ap4m1, Tpp1, Ap3m1, Naglu, Ppt2, Ctsf, Gaa, Lgmn, Ap3b1, Hgsnat, Mcoln1, Smpd1, Ctsb, Arsg, Neu1, Slc11a2, Ppt1 |
| KEGG_OXIDATIVE_  PHOSPHORYLATION | 0.0000 | 0.0000 | 0.8267 | 0.5946 | 2.2687 | 122 | Cox6c, Atp5j2, Atp5d, Atp5c1, Uqcrb, Atp6v0e, Atp5e, Atp6v1b1, Ndufb10, Uqcrfs1, Ndufs7, Ndufa1, Cox6a1, Atp6v0b, Cox7a2l, Ndufa10, Ndufb6, Cox6b1, Atp6v0d1, Ndufv3, Ndufa6, Atp5j, Atp6v1d, Cox7b, Ndufa11, Ndufs6, Ndufa8, Atp5h, Atp5k, Cox17, Ndufa2, Atp6ap1, Atp5g3, Atp5o, Cox7a2, Sdhd, Cox4i1, Atp5g1, Ndufv2, Uqcrh, Atp5pb, Uqcrq, Ndufs2, Ndufb1, Ndufb7, Cox5a, Atp5g2, Atp5l, Ndufs4, Ndufc1, Ndufv1, Ndufa3, Cox5b, Ndufa7, Ndufs3, Atp6v1e1, Ndufa5, Uqcr11, Ndufb5, Ndufs8, Sdhc, Atp6v1b2, Ndufab1, Ndufb9, Atp5a1, Cox15, Atp5b, Sdhb, Ppa1, Atp6v0a4, Ndufa4l2, Ndufa4, Uqcr10, Cox8a, Atp6v1f, Atp6v0a1, Ndufc2, Cox7b2, Cox11, Atp4a, Uqcrc1, Ndufa9, Ndufs5, Ndufb2, Ndufb3, Cyc1, Atp6v1c1, mt-Nd3, Atp6v0c |
| KEGG_PROTEIN_EXPORT | 0.0000 | 0.0000 | 0.8267 | 0.8664 | 2.4440 | 23 | Srpr, Sec61a1, Sec61g, Spcs1, Sec63, Immp2l, Spcs3, Spcs2, Sec62, Srprb, Srp14, Sec11c, Sec61b, Srp68, Oxa1l, Sec11a, Srp72, Srp19, Srp9 |
| KEGG_N_GLYCAN_BIOSYNTHESIS | 0.0000 | 0.0000 | 0.7477 | 0.7294 | 2.3467 | 45 | Mgat4a, Fut8, Rpn2, Stt3a, Alg5, Dpagt1, Ddost, Alg2, Alg11, Mgat4b, Man1a, Man2a2, Alg3, Alg9, Man1c1, B4galt2, Alg1, Man1b1, Mgat3, Dad1, Rpn1, Mogs, B4galt3, Rft1, Ganab, Dpm2, B4galt1, Tusc3, Mgat2, Alg10b, Alg6, Man2a1 |
| KEGG_PARKINSONS_DISEASE | 0.0000 | 0.0000 | 0.6594 | 0.5302 | 2.0232 | 122 | Ube2j1, Cox6c, Atp5d, Atp5c1, Uqcrb, Atp5e, Ndufb10, Slc25a5, Uqcrfs1, Ndufs7, Ndufa1, Cox6a1, Cox7a2l, Ndufa10, Ndufb6, Cox6b1, Vdac2, Ndufv3, Ndufa6, Atp5j, Septin5, Cox7b, Ube2g2, Ndufs6, Ndufa8, Atp5h, Ndufa2, Htra2, Atp5g3, Atp5o, Cox7a2, Sdhd, Pink1, Cox4i1, Atp5g1, Ndufv2, Uqcrh, Atp5pb, Uqcrq, Ndufs2, Ndufb1, Ndufb7, Cox5a, Atp5g2, Ndufs4, Ndufc1, Ndufv1, Ndufa3, Cox5b, Ndufa7, Ndufs3, Ndufa5, Vdac1, Ube2j2, Uqcr11, Ndufb5, Ndufs8, Sdhc, Ndufab1, Ndufb9, Atp5a1, Atp5b, Sdhb, Casp9, Ndufa4l2, Ndufa4, Uqcr10, Cox8a, Ndufc2, Cox7b2, Snca, Uqcrc1, Ndufa9, Ndufs5, Ndufb2 |
| KEGG_ALZHEIMERS_DISEASE | 0.0000 | 0.0000 | 0.6436 | 0.4843 | 1.9076 | 157 | Lpl, Eif2ak3, Ern1, Cox6c, Atp5d, Lrp1, Atp5c1, Hsd17b10, Uqcrb, Atp5e, Ndufb10, Uqcrfs1, Ndufs7, Ndufa1, Cox6a1, Psen2, Cox7a2l, Atf6, Ndufa10, Ndufb6, Cox6b1, Ndufv3, Ndufa6, Atp5j, Cox7b, Ndufs6, Ndufa8, Atp5h, Psen1, Ndufa2, Atp5g3, Atp5o, Cox7a2, Sdhd, Cox4i1, Atp5g1, Ndufv2, Uqcrh, Adam10, Atp5pb, Uqcrq, Ndufs2, Ppp3ca, Ndufb1, Ndufb7, Cox5a, Atp5g2, Ndufs4, Ndufc1, Ndufv1, Nos1, Tnf, Ndufa3, Cox5b, Ndufa7, Ndufs3, Ndufa5, Uqcr11, Ndufb5, Ndufs8, Sdhc, Ndufab1, Bace2, Ndufb9, Tnfrsf1a, Atp5a1, Atp5b, Psenen, Ppp3r1, Sdhb, Mapk1, Casp9, Ndufa4l2, Ndufa4, Uqcr10, Cox8a, Ndufc2, Cox7b2, Calm3, Snca, Uqcrc1, Ndufa9, Ndufs5, Ndufb2 |
| KEGG_VASCULAR_SMOOTH_MUSCLE_CONTRACTION | 0.0000 | 0.0009 | 0.5573 | -0.4714 | -1.8398 | 105 | Npr1, Adcy4, Plcb4, Acta2, Plcb1, Gucy1a1, Pla2g1b, Actg2, Prkch, Adcy2, Myh11, Ppp1r12b, Prkcg, Myl9, Itpr1, Ramp2, Agtr1a, Arhgef12, Arhgef1, Map2k1, Kcnmb1, Ppp1r12a, Adora2b, Adcy5, Cacna1d, Rock1, Kcnma1, Calm1, Prkcq, Adra1a, Calm2, Calcrl, Prkcb, Raf1, Mylk2, Rock2, Avpr1b, Adcy6, Adra1b, Adora2a, Mylk, Pla2g5, Gnaq, Cacna1c, Cald1, Adra1d, Prkce |
| KEGG_HUNTINGTONS_DISEASE | 0.0001 | 0.0013 | 0.5384 | 0.4274 | 1.7001 | 169 | Creb3, Cox6c, Atp5d, Polr2j, Atp5c1, Uqcrb, Ap2s1, Atp5e, Ndufb10, Slc25a5, Uqcrfs1, Ndufs7, Ndufa1, Cox6a1, Cox7a2l, Ndufa10, Polr2e, Ndufb6, Cox6b1, Clta, Vdac2, Ndufv3, Ap2a1, Ndufa6, Atp5j, Cox7b, Ndufs6, Ndufa8, Atp5h, Ap2a2, Ndufa2, Atp5g3, Atp5o, Cox7a2, Sdhd, Cox4i1, Atp5g1, Ndufv2, Uqcrh, Atp5pb, Uqcrq, Ndufs2, Ndufb1, Ndufb7, Cox5a, Atp5g2, Ndufs4, Ndufc1, Ndufv1, Polr2g, Ndufa3, Cox5b, Ndufa7, Ndufs3, Sod1, Dctn2, Ndufa5, Vdac1, Uqcr11, Ndufb5, Bax, Ndufs8, Sdhc, Ndufab1, Polr2i, Ndufb9, Atp5a1, Atp5b, Sdhb, Casp9, Ndufa4l2, Ndufa4, Uqcr10, Cox8a, Tfam, Ndufc2, Cox7b2, Uqcrc1, Ndufa9, Polr2c, Ndufs5, Rcor1, Ndufb2, Creb3l4, Sod2, Ndufb3, Cyc1, Creb3l3, Dnal1 |
| KEGG_AXON_GUIDANCE | 0.0001 | 0.0014 | 0.5384 | -0.4478 | -1.7979 | 127 | Sema3g, Sema3f, Ablim3, Efnb2, Nfat5, Sema3c, Rgs3, Sema6a, Rnd1, Ephb1, Sema3b, Ablim1, Sema3e, Fes, Sema6d, Efna1, Cxcl12, L1cam, Met, Sema4f, Ephb4, Sema7a, Sema5a, Arhgef12, Pak6, Plxnb1, Gnai2, Ntn4, Cxcr4, Srgap3, Limk2, Srgap2, Sema6b, Sema4c, Abl1, Rock1, Ptk2, Efna5, Efnb1, Ephb6 |
| KEGG_CALCIUM_SIGNALING_PATHWAY | 0.0001 | 0.0016 | 0.5384 | -0.3976 | -1.6679 | 164 | Itpkb, Adcy4, Ednrb, Plcb4, Plcb1, Atp2b4, Nos3, Atp2a3, Cysltr1, Pln, Adcy2, Adrb2, Bst1, Ryr2, Prkcg, Plce1, Cd38, Itpr1, Tbxa2r, Erbb4, Camk2b, Bdkrb2, Agtr1a, Cacna1i, Plcd1, Cysltr2, Ptafr, Ptgfr, Ryr3, Erbb3, Plcg1, Adora2b, Gna14, Bdkrb1, Camk2d, Cacna1d, Hrh2, P2rx6, Grin1, Oxtr, Calm1, Htr2b, Adra1a, Pde1b, Calm2, Phka1, Atp2b2, Ppp3cc, Phka2, Cckbr, Prkcb, Mylk2, Avpr1b, Cacna1h, Drd1, Adra1b, Adora2a, Sphk2, Mylk, Ltb4r2, Chrm2, Gnaq, Grm5, Cacna1c, Camk4, Adra1d, Grin2a, Slc8a2, Cacna1b, Mylk3, Atp2b1, Prkacb, Nos2, Erbb2 |
| KEGG_LEUKOCYTE_TRANSENDOTHELIAL_MIGRATION | 0.0001 | 0.0016 | 0.5384 | -0.4526 | -1.7855 | 110 | Pecam1, Esam, Cdh5, Jam2, Cldn15, Mmp9, Actn4, Rapgef3, Pxn, Prkcg, Afdn, Myl9, Cxcl12, Msn, Rapgef4, Sipa1, Ezr, Ctnna1, Cldn5, Mapk12, Cldn11, Gnai2, Plcg1, Pik3cg, Itga4, Cxcr4, Mapk11, Pik3r3, Rock1, Ptk2, Mylpf, Itk, Ctnna3, Arhgap35, Rassf5, Arhgap5, Actn3, Prkcb, Rock2, Jam3, Ctnnd1, Myl12b, Vav1 |
| KEGG_P53_SIGNALING_PATHWAY | 0.0001 | 0.0018 | 0.5188 | 0.5297 | 1.8268 | 66 | Gadd45g, Igf1, Rprm, Perp, Gadd45a, Serpine1, Thbs1, Ccnd2, Ccng1, Rchy1, Pmaip1, Rrm2, Cop1, Cdk4, Serpinb5, Ddb2, Sesn3, Ccnb1, Bax, Zmat3, Sesn2, Cdk1, Ccnb2, Casp9, Chek1, Cd82 |
| KEGG_CELL_ADHESION_MOLECULES_CAMS | 0.0001 | 0.0018 | 0.5188 | -0.4316 | -1.7247 | 119 | Pecam1, Esam, Cdh5, Cadm1, Jam2, Itga9, Cd274, Cldn15, Icam2, Cdh3, Itgb8, Itgb7, L1cam, Cntnap2, Cldn5, Selp, Sele, Neo1, Cldn11, Ncam1, Cadm3, Cd2, Itga4, Sell, Cd28, Alcam, Mag, Cntn1, Cd34, Ptprm, Cdh4, Nectin3, Mpzl1, H2-DMb1 |
| KEGG_REGULATION_OF_ACTIN_  CYTOSKELETON | 0.0001 | 0.0018 | 0.5188 | -0.3709 | -1.5895 | 204 | Pdgfc, Itga9, Pdgfd, Fgf12, Fgfr4, Myh10, Fgf1, Iqgap1, Actn4, Myh14, Fgfr2, Itga7, Pxn, Itga1, Fgfr3, Itgb8, Myl9, Enah, Msn, Bdkrb2, Itgb7, Diaph1, Arhgef12, Arhgef1, Ezr, Pak6, Dock1, Map2k1, Iqgap2, Pik3cg, Itga4, Pdgfb, Ppp1r12a, Limk2, Itga5, Itga3, Bdkrb1, Pik3r3, Fgd3, Rock1, Pip4k2a, Ptk2, Cyfip1, Mylpf, Fgf18, Ins2, Arhgap35, Rdx, Fgf13, Scin, Chrm4, Actn3, Baiap2, Raf1, Rras, Mylk2, Rock2, Fgf9, Pfn4, Crkl, Sos2, Fgfr1, Myl12b, Vav1, Ssh1, Mylk, Chrm2, Fn1, Arhgef7, Pak5, Diaph2 |
| KEGG_FOCAL_ADHESION | 0.0003 | 0.0037 | 0.4985 | -0.3767 | -1.6041 | 195 | Cav1, Parvb, Flt4, Lama3, Flt1, Reln, Kdr, Pdgfc, Itga9, Pgf, Pdgfd, Cav2, Lamc2, Flnb, Lamb2, Actn4, Vegfa, Flna, Vwf, Itga7, Pxn, Itga1, Prkcg, Ccnd1, Col11a2, Itgb8, Myl9, Col4a6, Xiap, Tln1, Itgb7, Met, Diaph1, Vtn, Lama4, Pak6, Dock1, Map2k1, Pik3cg, Comp, Itga4, Pdgfb, Ppp1r12a, Itga5, Itga3, Pik3r3, Rock1, Rasgrf1, Ptk2, Rapgef1, Mylpf |
| KEGG_CHEMOKINE_SIGNALING_PATHWAY | 0.0007 | 0.0080 | 0.4773 | -0.3713 | -1.5543 | 167 | Adcy4, Plcb4, Ccl21a, Plcb1, Nfkbia, Adcy2, Gngt2, Pxn, Cxcl12, Cxcl9, Ccl5, Cxcl10, Lyn, Pf4, Ccl24, Map2k1, Gnai2, Pik3cg, Rasgrp2, Cxcr4, Grk5, Cxcl13, Adcy5, Pik3r3, Rock1, Ptk2, Jak3, Grk4, Itk, Jak2, Stat2, Ccl6, Ikbkb, Elmo1, Prkcb, Raf1, Rock2, Crkl, Cxcr5, Adcy6, Ccl9, Sos2, Prex1, Vav1, Shc2, Gng4, Gng11, Prkacb, Gsk3b, Xcr1, Plcb3, Cxcl3, Prkcd, Sos1, Foxo3, Pik3cb, Dock2, Cxcl2, Shc3, Prkx, Nfkb1, Shc4, Pik3cd, Pard3, Ptk2b, Rac2, Gng7, Wasl, Akt3, Ccl12, Vav3 |
| KEGG_MELANOGENESIS | 0.0008 | 0.0084 | 0.4773 | -0.4371 | -1.6746 | 95 | Edn1, Adcy4, Ednrb, Plcb4, Plcb1, Wnt2b, Adcy2, Prkcg, Camk2b, Ep300, Wnt3, Map2k1, Gnai2, Wnt4, Wnt7b, Fzd8, Kitl, Fzd6, Adcy5, Camk2d, Fzd4, Fzd7, Kit, Gnao1, Fzd10, Crebbp, Calm1, Calm2, Prkcb, Raf1, Fzd5, Adcy6, Mitf |
| KEGG_MAPK_SIGNALING_PATHWAY | 0.0014 | 0.0137 | 0.4551 | -0.3353 | -1.4736 | 251 | Rasgrf2, Rasgrp3, Fgf12, Fgfr4, Fgf1, Flnb, Dusp2, Mef2c, Pla2g1b, Flna, Fgfr2, Rapgef2, Fgfr3, Prkcg, Rasgrp1, Taok2, Map3k1, Hspa2, Ptprr, Tgfb2, Tgfb3, Cacna1i, Dusp3, Mapt, Map3k8, Rps6ka5, Mapk12, Mapk8ip2, Map2k1, Fas, Pdgfb, Map4k3, Rasgrp2, Mknk2, Mapk11, Ptpn7, Elk4, Ntrk2, Cacna1d, Nfkb2, Ntf3, Rasgrf1, Map3k11, Map3k3, Taok1, Mapk8ip3, Fgf18, Cacna2d4, Mecom, Map3k12, Fgf13, Gadd45b, Daxx, Map3k13, Mapk8ip1, Ppp3cc, Map3k2, Casp3, Ikbkb, Map3k14, Prkcb, Raf1, Rras, Map4k2, Fgf9, Crkl, Hspa1l, Cacna1h, Cacna2d2, Map3k20, Trp53, Map3k5, Sos2, Fgfr1, Map3k6, Map2k6, Rasa1, Rps6ka6, Dusp16, Rasgrp4, Pla2g5 |
| KEGG_PATHWAYS_IN_CANCER | 0.0017 | 0.0163 | 0.4551 | -0.3084 | -1.3968 | 329 | Lama3, Epas1, Pgf, Fgf12, Nfkbia, Lamc2, Pld1, Ets1, Csf1r, Fgf1, Rarb, Lamb2, Wnt2b, Mmp9, Vegfa, Fgfr2, Axin2, Fgfr3, Prkcg, Ccnd1, Col4a6, Ret, Xiap, Met, Traf5, Rb1, Ptch1, Cbl, Tgfb2, Cdkn1b, Tgfb3, Tgfa, Lama4, Foxo1, Ep300, Ctnna1, Dapk1, Wnt3, Ralgds, Rara, Map2k1, Fas, Csf2ra, Plcg1, Pik3cg, Wnt4, Pdgfb, Ncoa4, Wnt7b, Fzd8, Kitl, Msh2, Bmp2, Fzd6, Itga3, Pik3r3, Abl1, Nfkb2, Hif1a, Fzd4, Fzd7, Kit, Fzd10, Ptk2, Crebbp, Fgf18, Traf1, Slc2a1, Bcl2l1, Tpr, Sufu, Ctnna3, Mecom, Cdk2, Fgf13, Mlh1, Flt3, Rassf5, Casp3, Ikbkb, Hdac1, Prkcb, Raf1, Fzd5, Fgf9, Smo, Bcr, Crkl |
| KEGG_STEROID_BIOSYNTHESIS | 0.0019 | 0.0165 | 0.4551 | 0.7037 | 1.8595 | 17 | Soat2, Cel, Sc5d, Ebp, Soat1, Cyp51, Dhcr24, Dhcr7, Nsdhl, Sqle |
| KEGG_PHOSPHATIDYLINOSITOL_SIGNALING_SYSTEM | 0.0020 | 0.0167 | 0.4317 | -0.4587 | -1.6778 | 74 | Itpkb, Plcb4, Cds2, Plcb1, Prkcg, Plce1, Itpr1, Pik3c2b, Inpp4a, Inpp4b, Plcd1, Inpp5d, Dgke, Itpk1, Plcg1, Pik3cg, Dgkh, Pik3c2a, Pik3r3, Inpp5k, Pip4k2a, Calm1 |
| KEGG_AMINOACYL_TRNA_BIOSYNTHESIS | 0.0024 | 0.0197 | 0.4317 | 0.5547 | 1.7474 | 41 | Wars1, Lars2, Sars, Mars1, Cars, Qars, Gars, Yars, Vars, Aars, Kars, Nars, Pstk, Iars, Tars |
| KEGG_RIBOFLAVIN_METABOLISM | 0.0033 | 0.0257 | 0.4317 | 0.7164 | 1.7772 | 14 | Enpp1, Acp5, Acp2, Acpp, Rfk |
| KEGG_NEUROACTIVE_LIGAND_RECEPTOR_INTERACTION | 0.0037 | 0.0278 | 0.4317 | 0.3487 | 1.4208 | 216 | Ghr, Try5, P2rx4, Prss3, Gm10334, Prss2, F2r, P2ry1, F2, Vipr1, Gria3, Ptger3, Ptger4, Vipr2, Prss1, Cckar, Adcyap1r1, Avpr1a, Gabra4, S1pr2, Lpar1, Ednra, Chrm3, Pth1r, Gabrp, Adrb1, P2rx1, Glp1r, Mas1 |
| KEGG_TIGHT_JUNCTION | 0.0042 | 0.0301 | 0.4070 | -0.3855 | -1.5509 | 123 | Ppp2r2b, Jam2, Sptan1, Myh10, Ppp2r2c, Cldn15, Actn4, Myh14, Prkch, Cgn, Myh11, Magi3, Magi1, Prkcg, Afdn, Tjp2, Myl9, Epb41l1, Tjp1, Ctnna1, Cldn5, Cldn11, Gnai2, Ppp2r2a, Prkci, Epb41l3, Mylpf, Prkcq, Ctnna3, Llgl2, Ash1l, Patj, Exoc3, Pals1, Actn3, Hcls1, Prkcb, Rras, Sympk, Jam3 |
| KEGG_ENDOCYTOSIS | 0.0049 | 0.0340 | 0.4070 | -0.3460 | -1.4550 | 178 | Flt1, Kdr, Arap3, Ehd4, Fgfr4, Pld1, Csf1r, Ehd2, Acap1, Fgfr2, Adrb2, Tfrc, Fgfr3, Pld2, Hspa2, Erbb4, Ret, Met, Dnm3, Cbl, Ehd3, Rab11fip2, Iqsec1, Iqsec3, Agap1, Dab2, Erbb3, Cxcr4, Arap2, Il2rg, Grk5, Sh3gl3, Nedd4l, Prkci, Git2, Kit, Rabep1, Epn2, Grk4, Epn3, Il2rb, Asap2 |
| KEGG_INOSITOL_PHOSPHATE_METABOLISM | 0.0053 | 0.0341 | 0.4070 | -0.4610 | -1.5805 | 54 | Itpkb, Plcb4, Plcb1, Plce1, Pik3c2b, Inpp4a, Inpp4b, Plcd1, Itpk1, Plcg1, Pik3cg, Ipmk, Pik3c2a, Inpp5k, Pip4k2a |
| KEGG_LONG_TERM_POTENTIATION | 0.0053 | 0.0341 | 0.4070 | -0.4457 | -1.6134 | 66 | Plcb4, Plcb1, Rapgef3, Prkcg, Itpr1, Camk2b, Ep300, Map2k1, Ppp1r12a, Camk2d, Crebbp, Grin1, Calm1, Calm2, Ppp3cc, Prkcb, Raf1, Rps6ka6, Gnaq, Grm5, Cacna1c, Camk4, Grin2a, Gria2, Prkacb, Plcb3, Rps6ka3, Prkx, Itpr3 |
| KEGG_VIBRIO_CHOLERAE_INFECTION | 0.0068 | 0.0410 | 0.4070 | 0.5013 | 1.6697 | 51 | Kdelr3, Sec61a1, Kdelr2, Sec61g, Kdelr1, Atp6v0e, Atp6v1b1, Prkca, Atp6v0b, Sec61b, Arf1, Atp6v0d1, Atp6v1d, Atp6ap1, Gnas, Muc2, Actb, Atp6v1e1, Actg1, Atp6v1b2 |
| KEGG_OTHER_GLYCAN_DEGRADATION | 0.0068 | 0.0410 | 0.4070 | 0.6854 | 1.7660 | 16 | Hexa, Fuca2, Glb1, Gba, Fuca1, Man2b2, Aga, Man2b1, Neu1, Engase |
| KEGG_AMINO_SUGAR_AND_NUCLEOTIDE_SUGAR_METABOLISM | 0.0076 | 0.0440 | 0.4070 | 0.5082 | 1.6294 | 44 | Nans, Hexa, Gfus, Gnpnat1, Gmds, Gnpda1, Gmppa, Pmm2, Gmppb, Galk1, Gpi1, Mpi, Pmm1, Pgm2, Amdhd2, Gfpt1, Pgm1, Pgm3, Gnpda2, Fcsk, Gale, Hk2, Cmas |
| KEGG_PROTEASOME | 0.0086 | 0.0485 | 0.3807 | 0.5267 | 1.6752 | 42 | Psmd8, Psmb6, Psmb7, Psmd12, Psmb1, Psmd7, Psmd6, Psmd3, Psmc3, Psmc1, Psma4, Pomp, Psmc5, Sem1, Psmb5, Psma3, Psma7, Psmc2, Psmd13, Psmd11, Psmd4, Psmd2, Psmb10, Psma8, Psmb4, Psmf1 |
| KEGG_JAK_STAT_SIGNALING_PATHWAY | 0.0094 | 0.0516 | 0.3807 | 0.3869 | 1.4662 | 117 | Ghr, Il6, Socs3, Il22ra1, Socs2, Ccnd2, Spry1, Tslp, Akt2, Myc, Il11ra1, Il23a, Crlf2, Ifngr2, Clcf1, Lif, Pik3r1, Socs1, Il22ra2, Spry2, Pik3r2, Spred1, Jak1, Akt1 |
| KEGG_DORSO_VENTRAL_AXIS_FORMATION | 0.0110 | 0.0585 | 0.3807 | -0.5864 | -1.6840 | 21 | Notch2, Notch4, Ets1, Notch3, Notch1, Map2k1, Spire2 |
| KEGG_CYTOKINE_CYTOKINE_RECEPTOR_INTERACTION | 0.0124 | 0.0630 | 0.3807 | -0.3195 | -1.3657 | 198 | Flt4, Flt1, Kdr, Pdgfc, Ccl21a, Tnfsf10, Csf1r, Bmpr2, Vegfa, Tnfrsf19, Tnfrsf1b, Cxcl12, Met, Cxcl9, Edar, Tgfb2, Tgfb3, Ccl5, Lepr, Csf3, Cxcl10, Relt, Inhbb, Tnfrsf25, Pf4, Ccl24, Fas, Csf2ra, Pdgfb, Cxcr4, Il2rg, Kitl, Bmp2, Tnfrsf13b, Cxcl13, Il18, Inhba, Kit, Il2rb, Il12rb2, Tnfrsf13c, Ccl6, Flt3, Ifnar1, Ifnlr1, Tnfsf13b, Csf2 |
| KEGG_PRIMARY_IMMUNODEFICIENCY | 0.0127 | 0.0630 | 0.3807 | -0.5303 | -1.6561 | 31 | Blnk, Tap1, Tap2, Cd19, Il2rg, Tnfrsf13b, Jak3, Zap70, Tnfrsf13c, Rfxank, Dclre1c, Icos |
| KEGG_NOTCH_SIGNALING_PATHWAY | 0.0130 | 0.0630 | 0.3807 | -0.4563 | -1.5312 | 47 | Jag2, Notch2, Notch4, Dll4, Notch3, Notch1, Mfng, Dll1, Ep300, Numb, Crebbp, Maml1, Hdac1, Dtx3, Kat2b, Dtx3l, Adam17 |
| KEGG_HEMATOPOIETIC_CELL_LINEAGE | 0.0132 | 0.0630 | 0.3807 | -0.3933 | -1.4446 | 75 | Cd36, Csf1r, Tfrc, Itga1, Cd38, Fcer2a, Csf3, Csf2ra, Cd2, Itga4, Cd19, Kitl, Itga5, Itga3, Cd34, Kit, Cd9, Flt3, Mme, Csf2, Dntt, Cd22 |
| KEGG_STEROID_HORMONE_BIOSYNTHESIS | 0.0137 | 0.0635 | 0.3807 | 0.5261 | 1.6029 | 34 | Ugt2b34, Cyp3a13, Ugt2a3, Hsd11b1, Cyp1b1, Hsd17b12, Srd5a3 |
| KEGG_RENAL_CELL_CARCINOMA | 0.0146 | 0.0662 | 0.3807 | -0.4056 | -1.4724 | 70 | Epas1, Pgf, Ets1, Vegfa, Met, Tgfb2, Tgfb3, Tgfa, Ep300, Pak6, Map2k1, Pik3cg, Pdgfb, Gab1, Pik3r3, Hif1a, Rapgef1, Crebbp, Slc2a1 |
| KEGG_B_CELL_RECEPTOR_SIGNALING_PATHWAY | 0.0155 | 0.0686 | 0.3807 | -0.4113 | -1.5045 | 74 | Rasgrp3, Nfkbia, Nfat5, Blnk, Inpp5d, Lyn, Malt1, Dapp1, Map2k1, Pik3cg, Cd19, Syk, Cd79b, Pik3r3, Ppp3cc, Ikbkb, Pik3ap1, Prkcb, Raf1, Cd22, Cd81, Sos2, Vav1, Cr2, Gsk3b, Rac3, Sos1, Pik3cb, Btk, Nfkb1, Nfatc1, Pik3cd, Fcgr2b, Rac2, Akt3, Vav3 |
| KEGG_ADHERENS_JUNCTION | 0.0166 | 0.0717 | 0.3525 | -0.4155 | -1.5192 | 73 | Ptprb, Iqgap1, Actn4, Lmo7, Afdn, Tjp1, Met, Ep300, Ctnna1, Nectin4, Ptprm, Crebbp, Ctnna3, Nectin3, Sorbs1, Ptprj, Actn3, Fer, Baiap2, Snai2, Ctnnd1, Fgfr1, Igf1r, Lef1, Nlk, Yes1, Erbb2, Rac3, Cdh1, Ctnnb1, Pard3, Rac2, Ssx2ip, Wasl, Wasf2, Tcf7l1, Ptprf, Nectin2, Was |
| KEGG_COMPLEMENT_AND_COAGULATION_CASCADES | 0.0170 | 0.0721 | 0.3525 | 0.4336 | 1.4951 | 66 | Plau, Kng2, Serpine1, F2r, F2, Serpina5, C4bp, Serping1, Plat, Cfi, C4b, Cfh, Fgb, Pros1, Cfb |
| KEGG_SNARE_INTERACTIONS_IN_VESICULAR_TRANSPORT | 0.0191 | 0.0788 | 0.3525 | 0.5148 | 1.5686 | 34 | Ykt6, Sec22b, Bet1, Vti1b, Use1, Gosr2, Vamp8, Stx11, Vamp4, Bnip1, Stx12, Stx5a, Vti1a, Snap23, Stx8 |
| KEGG_O_GLYCAN_BIOSYNTHESIS | 0.0203 | 0.0801 | 0.3525 | 0.5495 | 1.6105 | 28 | Galnt7, Galntl6, Galnt17, Galnt3, Gcnt4, B4galt5, C1galt1c1, Gcnt1, Galnt1, Galnt2, Galnt4, Galnt6, Galnt5 |
| KEGG_GLYCOSPHINGOLIPID_BIOSYNTHESIS_GANGLIO_SERIES | 0.0203 | 0.0801 | 0.3525 | 0.6410 | 1.5902 | 14 | B4galnt1, Hexa, Glb1, B3galt4, St6galnac4, Slc33a1 |
| KEGG_MISMATCH_REPAIR | 0.0207 | 0.0801 | 0.3525 | -0.5581 | -1.6322 | 22 | Mlh3, Pms2, Pcna, Msh2, Pold3, Mlh1, Rpa1, Rpa3, Lig1, Rpa2 |
| KEGG_WNT_SIGNALING_PATHWAY | 0.0229 | 0.0870 | 0.3525 | -0.3252 | -1.3284 | 143 | Sox17, Plcb4, Plcb1, Nfat5, Wnt2b, Axin2, Prkcg, Ccnd1, Nkd1, Camk2b, Sfrp2, Ep300, Sfrp5, Wnt3, Wnt4, Wnt7b, Fzd8, Fzd6, Dkk2, Camk2d, Rock1, Fzd4, Fzd7, Fzd10, Crebbp, Ppp2r5a, Ppp3cc, Chd8, Prkcb, Fzd5, Rock2, Trp53, Tbl1xr1, Lrp6, Lrp5, Lef1, Nlk, Csnk1e, Prkacb, Gsk3b, Fbxw11, Rac3, Sfrp4, Plcb3, Wnt9a, Ctnnb1, Mapk8, Prkx, Mapk10, Nfatc1 |
| KEGG_SELENOAMINO_ACID_METABOLISM | 0.0244 | 0.0909 | 0.3525 | 0.5368 | 1.5487 | 26 | Sephs2, Ggt6, Mars1, Ahcy, Trmt11, Mat2b, Lcmt1, Ggt5, Cbs, Cth, Scly, Ahcyl1, Mat1a, Bud23 |
| KEGG_BUTANOATE_METABOLISM | 0.0252 | 0.0919 | 0.3525 | 0.5089 | 1.5124 | 31 | Acat1, Oxct1, Acads, Acsm3, Abat, Echs1, Bdh1, Hadh, Akr1b8, Aldh5a1, Aldh9a1, Pdha1, Aldh3a2, Aldh2, Acsm2 |
| KEGG_VALINE_LEUCINE_AND_ISOLEUCINE_DEGRADATION | 0.0264 | 0.0944 | 0.3525 | 0.4631 | 1.4775 | 43 | Hsd17b10, Acat1, Oxct1, Hibadh, Acads, Abat, Echs1, Bckdhb, Bcat2, Hadh, Auh, Acaa1a, Bckdha, Aldh9a1, Hibch, Aldh3a2, Acad8 |
| KEGG_GAP_JUNCTION | 0.0290 | 0.1016 | 0.3525 | -0.3814 | -1.4209 | 82 | Pdgfc, Adcy4, Plcb4, Pdgfd, Plcb1, Gucy1a1, Adcy2, Tubb4a, Prkcg, Itpr1, Gja1, Tjp1, Map2k1, Gnai2, Pdgfb, Adcy5, Htr2b, Tubb2a, Tubb4b, Map3k2, Prkcb, Raf1, Adcy6, Drd1, Sos2, Gnaq, Grm5, Tubb3 |
| KEGG_GLYCOSYLPHOSPHATIDYLINOSITOL_GPI_ANCHOR_BIOSYNTHESIS | 0.0315 | 0.1087 | 0.3218 | 0.5358 | 1.5251 | 25 | Pigf, Pigt, Pigq, Gpaa1, Pigo, Dpm2, Pigw, Pigs, Pigu |
| KEGG_GNRH_SIGNALING_PATHWAY | 0.0384 | 0.1298 | 0.3218 | -0.3737 | -1.4291 | 91 | Adcy4, Plcb4, Plcb1, Pld1, Pla2g1b, Adcy2, Map3k1, Pld2, Itpr1, Camk2b, Mapk12, Map2k1, Mapk11, Adcy5, Camk2d, Cacna1d, Map3k3, Calm1, Calm2, Map3k2, Prkcb, Raf1, Adcy6, Sos2, Map2k6, Pla2g5, Gnaq, Cacna1c, Gnrhr, Map3k4, Prkacb, Plcb3, Pla2g4b, Map2k4, Prkcd, Sos1, Mapk7, Mapk8, Prkx, Mapk10, Itpr3 |
| KEGG_CITRATE_CYCLE_TCA_CYCLE | 0.0457 | 0.1517 | 0.3218 | 0.5200 | 1.5241 | 28 | Pcx, Mdh2, Suclg1, Pck2, Idh3b, Sdhd, Idh1, Cs, Sdhc, Idh3a, Dlst, Sdhb, Fh1, Pdha1, Suclg2, Mdh1, Dlat, Dld, Idh3g |
