# Supplementary material for: RalGAP complexes control secretion and primary cilia in pancreatic disease
Source: Life Sci Alliance. 2025 Jun 9;8(8):e202403123. doi: 10.26508/lsa.202403123 (PMC12149561; doi:10.26508/lsa.202403123)
Supplement: Supplementary file 2 [file LSA-2024-03123_TableS2.docx]

**Table S2 - GOBP pathway analysis of RGβKO acinar cells in comparison to WT acinar cells.** List of GOBP pathways obtained by gene set enrichment analysis after RNA sequencing from RGβKO and WT sorted acinar cells. Given are the GOBP pathway name, p-value, adjusted p-value, log2err, enrichment score (ES), normalized enrichment score (NES), gene set size and the gene names that contributed to the leading edge. Values are rounded to 4 decimal points and only the first 50 pathways are depicted sorted by declining NES.

| **GOBP pathway** | **p-value** | **adjusted p-value** | **log2err** | **ES** | **NES** | **size** | **Leading edge** |
| --- | --- | --- | --- | --- | --- | --- | --- |
| GOBP_CYTOPLASMIC_TRANSLATION | 0.0000 | 0.0000 | 1.4173 | 0.7522 | 2.9463 | 143 | Rps5, Rpl26, Rpl7a, Rps6, Rps17, Rps12, Rpl14, Rpl31, Rpsa, Rps26, Rpl36a, Rpl23, Rpl28, Rpl34, Rpl12, Rps20, Rps16, Rpl6, Rps8, Rpl37, Rpl10a, Rpl37a, Rps29, Rpl23a, Rpl4, Rpl19, Rpl13, Rpl35, Rps15, Rps14, Rps3a1, Rpl38, Rpl22, Eif2s2, Rps13, Rpl27a, Rps3, Rps11, Rps27a, Rpl3, Rplp0, Rpl8, Rpl15, Rps24, Rpl39, Rpl36, Rps9, Rps25, Rpl18a, Rpl7, Ybx3, Rps4x, Rps2, Rps7, Rplp1, Rpl32, Rpl5, Rps15a, Rps27, Rps19, Rpl17, Rpl22l1, Rpl27, Rplp2, Rpl18, Rpl11, Rpl10, Pabpc1, Rpl13a, Rps21, Rpl30, Ybx1, Rps28, Rpl9, Eif3a, Eif3i, Eif4b, Rpl29, Rps18, Eif2d, Eif3d, Aars, Etf1, Rpl35a, Zc3h15, Igf2bp1, Eif3m |
| GOBP_PROTEIN_N_LINKED_GLYCOSYLATION | 0.0000 | 0.0000 | 0.8871 | 0.7365 | 2.5682 | 64 | Slc39a8, Mgat4a, Fut8, Tmem258, Rpn2, Stt3a, Alg5, Krtcap2, Ube2j1, Magt1, Derl3, Dpagt1, Ddost, Alg2, Ostc, Alg11, Mgat4b, Mlec, Alg3, Nus1, Alg9, Ost4, Entpd5, Man1c1, Alg1, Srd5a3, Uggt1, Mgat3, Dad1, Rpn1, Pmm2, Mogs, Rft1, B4galt1, Pmm1, Mpdu1, Gal3st1, Tusc3, Dhdds, B4galt7, Mgat2, Nudt14, Gfpt1, Pgm3, Alg10b, Alg6, Dolk |
| GOBP_PROTEIN_LOCALIZATION_TO_ENDOPLASMIC_RETICULUM | 0.0000 | 0.0000 | 0.8140 | 0.6972 | 2.4291 | 65 | Kdelr3, Sec16b, Srpr, Sec61a1, Kdelr2, Ssr3, Sec61g, Kdelr1, Edem1, Spcs1, Tram1, Sec63, Ubac2, Chmp4b, Spcs3, Man1a, Spcs2, Sec62, Srprb, Srp14, Os9, Sec61b, Mia3, Get3, Get4, Ddrgk1, Gpaa1, Rer1, Herpud1, Srp68, Bcap31, Srp72, Pdia2, Insig1, Srp19, Btf3, Srp9 |
| GOBP_ENDOPLASMIC_RETICULUM_TO_CYTOSOL_TRANSPORT | 0.0000 | 0.0000 | 0.7477 | 0.8078 | 2.4137 | 28 | Derl1, H13, Edem1, Ube2j1, Sel1l, Derl3, Selenos, Aup1, Erlec1, Ubac2, Edem2, Os9, Sec61b, Rhbdd1, Tmem129, Ube2g2, Herpud1, Brsk2, Bcap31, Svip, Nploc4 |
| GOBP_MITOCHONDRIAL_TRANSMEMBRANE_TRANSPORT | 0.0000 | 0.0000 | 0.8634 | 0.6350 | 2.3832 | 97 | Slc39a8, Dnajc19, Slc8b1, Atp5j2, Atp5d, Atp5c1, Slc25a1, Atp5e, Grpel1, Dnlz, Slc25a5, Slc25a28, Mrpl18, Psen2, Maip1, Sfxn3, Smdt1, Timm23, Timm17b, Atp5j, Romo1, Tomm20, Bhlha15, Stoml2, Sfxn1, Slc25a3, Grpel2, Atp5h, Atp5k, Micu1, Pmpcb, Atp5o, Tomm70a, Dnajc30, Mcur1, Chchd4, Atp5pb, Tomm40l, Atp5l, Sfxn5, Slc25a22, Vdac1, Micu2, Tomm7, Antkmt, Timm44, Atp5a1, Atp5b, Slc25a33 |
| GOBP_PROTEIN_EXIT_FROM_ENDOPLASMIC_RETICULUM | 0.0000 | 0.0000 | 0.7477 | 0.7179 | 2.3744 | 48 | Sec16b, Tmem30b, Derl1, H13, Surf4, Edem1, Ube2j1, Sel1l, Lman1, Derl3, Selenos, Aup1, Erlec1, Ubac2, Edem2, Os9, Sec13, Sec61b, Rhbdd1, Tm9sf4, Tmem129, Ube2g2, Preb, Herpud1, Tmed9, Brsk2, Sar1a, Bcap31, Svip, Insig1, Nploc4 |
| GOBP_ENDOPLASMIC_RETICULUM_TO_GOLGI_VESICLE_MEDIATED_TRANSPORT | 0.0000 | 0.0000 | 0.8634 | 0.6109 | 2.3710 | 123 | Kdelr3, Sec16b, Tmed3, Kdelr2, Tmed10, Ykt6, Kdelr1, Tmed2, Scap, Sec22b, Lman1, Cope, Spast, Yipf5, Yif1a, Rab29, Arf4, Bet1, Yif1b, Ier3ip1, Rab2a, Ergic2, Sec13, Tfg, Rint1, Tmed4, Mia3, Use1, Tmed6, Gosr2, Ergic1, Tmed5, Tex261, Preb, Tmed9, Vapb, Pdcd6, Sar1a, Ergic3, Mia2, Lman2, Arcn1, Sec31a, Bcap31, Vamp4, Copg1, Tbc1d20, Insig1, Sec22c, Trappc6a, Rab1a, Trappc1, Trappc3, Stx5a, Copa, Sec23a, Sar1b, Vti1a, Sec24a, Ppp6c, Copb1 |
| GOBP_PEPTIDE_BIOSYNTHETIC_PROCESS | 0.0000 | 0.0000 | 1.4026 | 0.5182 | 2.3543 | 622 | Il6, Ang, Btg2, Krt17, Eif2ak3, Thbs1, Eif4ebp1, Rps5, Rpl26, Rpl7a, Wars1, Rps6, Rps17, Rps12, Rpl14, Rpl31, Rpsa, Rps26, Rpl36a, Rpl23, Rpl28, Rpl34, Tmed2, Rpl12, Rps20, Rps16, Rpl6, Rps8, Rpl37, Lars2, Rpl10a, Rpl37a, Rps29, Mrpl36, Eef2, Rpl23a, Rpl4, Rpl19, Sars, Rpl13, Mrpl52, Rpl35, Eef1d, Cyp1b1, Rps15, Pabpc4, Eef1b2, Rps14, Rps3a1, Tnip1, Rack1, Rpl38, Eef1a1, Rpl22, Eif2s2, Rps13, Rpl27a, Rps3, Eif3h, Rps11, Rps27a, Akt2, Rpl3, Rplp0, Rpl8, Zfp706, Paip2b, Rpl15, Rps24, Rpl39, Rpl36, Zfp598, Rps9, Eif5b, Rps25, Eif3f, Eif2s1, Rpl18a, Rpl7, Ybx3, Rps4x, Mtres1, Mrpl13, Rps2, Rps27l, Ggt6, Rps7, Mars1, Rplp1, Rpl32, Serp1, Rpl5, Mknk1, Rps15a, Rps27, Rps19, Rpl17, Mrpl18, Cars, Rpl22l1, Rpl27, Rplp2, Rrbp1, Rpl18, Rpl11, Rpl10, Eif3j1, Qars, Pabpc1, Hspb1, Rpl13a, Rps21, Rpl30, Mrps18c, Dnajc3, Ybx1, Gars, Yars, Rps28, Rpl9, Eif2a, Eif3a, Eif3i, Eif4b, Larp1b, Rpl29, Eif5a, Rps18, Exosc5, Npm1, Vars, Eif2d, Eif3l, Eif3g, Eif1ax, Mrps14, Eif2b5, Mrps28, Hbs1l, Noa1, Mrps11, Mrps18b, Mrpl35, Mrpl11, Upf3a, Cdc123, Aimp1, Mrps15, Mrpl28, Eif3d, Tyms, Aars, Etf1, Eif6, Mrpl2, Eif4e2, Mrpl43, Mrpl32, Atxn2, Lrrc47, Eef1g, Chac2, Mrpl58, Pink1, Neurl1a, Kars, Nars, Rpl35a, Larp1, Zc3h15, Aimp2, Cops5, Dhfr, Eif3k, Mrpl10, Mrpl23, Mrps24, Igf2bp1, Pkp3, Mrps7, Uqcc2, Pstk, Eif3m, Eif3e, Eif2b1, Rhoa, Dapk3, Mrpl55, Tarbp2, Eif2b4, Piwil2, Ngdn, Gclc, Eif2ak1, Coa3, Polr2g, Pa2g4, Akt1, Hagh, Cdkal1, Tnf, Lsm14b, Boll, Ndufa7, Cnot9, Magoh, Mrps34, Mrpl34, Abcf1, Rwdd1, Niban1, Mrpl16, Mrps21, Malsu1, Rps10, Srp9, Rpl21, Iars, Denr, Mrrf, Dhps, Eif2s3x, Mrpl57, Furin, Mrpl24, Slc1a1, Mtif3, Mrps2, Shmt2, Tars, Eif1ad, Ppp1ca, Noct, Dph3, Mrps5, Gspt1, Rps23, Calr, Tufm, Ggt5, Mrpl3, Ddx1, Tsfm, Eif3b, Eif3c, Sesn2, Gclm, Dnajc24, Chac1, Nt5c3b, Klhl25 |
| GOBP_ESTABLISHMENT_OF_PROTEIN_LOCALIZATION_TO_ENDOPLASMIC_RETICULUM | 0.0000 | 0.0000 | 0.6750 | 0.7243 | 2.3167 | 38 | Srpr, Sec61a1, Ssr3, Sec61g, Edem1, Spcs1, Tram1, Sec63, Chmp4b, Spcs3, Man1a, Spcs2, Sec62, Srprb, Srp14, Sec61b, Get3, Get4, Herpud1, Srp68, Srp72, Srp19, Srp9 |
| GOBP_REGULATION_OF_UBIQUITIN_PROTEIN_LIGASE_ACTIVITY | 0.0000 | 0.0000 | 0.6594 | 0.8011 | 2.3061 | 23 | Rpl23, Rps20, Rpl37, Rps15, Mad2l2, Rps7, Rpl5, Rpl11, Cdc20, Ube2c, Plk1, Fzr1, Mastl |
| GOBP_RIBOSOME_ASSEMBLY | 0.0000 | 0.0000 | 0.7195 | 0.6731 | 2.3007 | 57 | Rps5, Rpsa, Rpl6, Rpl23a, Rps15, Rps14, Rpl38, Rpl3, Rplp0, Rps27l, Rpl5, Rps27, Rps19, Rpl11, Rpl10, Rps28, Eif2a, Npm1, Mrpl20, Mrps11, Nop53, Rrs1, Eif6, Abt1, Nle1, Mrps7 |
| GOBP_PEPTIDE_METABOLIC_PROCESS | 0.0000 | 0.0000 | 1.4533 | 0.4960 | 2.2841 | 770 | Igf1, Clu, Ctsl, Il6, Ang, Btg2, Krt17, Eif2ak3, Thbs1, Eif4ebp1, Rps5, Cpxm1, Rpl26, Cpxm2, Rpl7a, Wars1, H13, Tmed10, Rps6, Rps17, Abcg1, Rps12, Rpl14, Rpl31, Rpsa, Rps26, Rpl36a, Cpn1, Ctns, Rpl23, Rpl28, Rpl34, Tmed2, Rpl12, Rps20, Rps16, Rpl6, Rps8, Rpl37, Spcs1, Trhde, Lars2, Rpl10a, Rpl37a, Rps29, Mrpl36, Eef2, Rpl23a, Rpl4, Rpl19, Sars, Rpl13, Mrpl52, Rpl35, Eef1d, Cyp1b1, Rps15, Pabpc4, Eef1b2, Rps14, Anpep, Rps3a1, Tnip1, Rack1, Rpl38, Xpnpep1, Eef1a1, Rpl22, Eif2s2, Rps13, Rpl27a, Rps3, Eif3h, Ethe1, Rps11, Rps27a, Akt2, Rpl3, Rplp0, Rpl8, Zfp706, Paip2b, Rpl15, Rps24, Rpl39, Rpl36, Zfp598, Rps9, Yipf5, Eif5b, Rtn3, Rps25, Eif3f, Eif2s1, Rpl18a, Rpl7, Ybx3, Rps4x, Mtres1, Mrpl13, Rps2, Rps27l, Ggt6, Rps7, Mars1, Rplp1, Rpl32, Serp1, Rpl5, Gsto1, Mknk1, Rps15a, Rps27, Rps19, Dpep1, Spcs3, Dnpep, Rpl17, Mrpl18, Cars, Rpl22l1, Spcs2, Rpl27, Rplp2, Rrbp1, Rpl18, Rpl11, Rpl10, Psen2, Eif3j1, Qars, Pabpc1, Hspb1, Rpl13a, Rps21, Rpl30, Mrps18c, Ctsz, Sec11c, Dnajc3, Ybx1, Gars, Yars, Rps28, Rpl9, Eif2a, Eif3a, Eif3i, Cpq, Eif4b, Slc2a13, Larp1b, Gstp1, Rpl29, Eif5a, Rps18, Exosc5, Npm1, Vars, Gstm2, Eif2d, Eif3l, Eif3g, Eif1ax, Mrps14, Eif2b5, Mrps28, Nat8f1, Ptges2, Parl, Hbs1l, Noa1, Mrps11, Mrps18b, Mrpl35, Mrpl11, Upf3a, Cln5, Cdc123, Psen1, Gstm5, Aimp1, Mrps15, Atp6ap2, Mrpl28, Eif3d, Tyms, Aars, Etf1, Eif6, Mrpl2, Eif4e2, Mrpl43, Mrpl32, Atxn2, Lrrc47, Aebp1, Mgat3, Eef1g, Chac2, Carnmt1, Mrpl58, Pink1, Lta4h, Neurl1a, Kars, Nars, Rpl35a, Larp1, Zc3h15, Aimp2, Cops5, Dhfr, Eif3k, Pcsk6, Idh1, Sec11a, Mrpl10, Mrpl23, Mrps24, Igf2bp1, Pkp3, Mrps7, Uqcc2, Pstk, Eif3m, Eif3e, Eif2b1, Tpp1, Rhoa, Dapk3, Mrpl55, Tarbp2, Eif2b4, Piwil2, Ngdn, Gclc, Eif2ak1, Coa3, Polr2g, Pa2g4, Akt1, Hagh, Cdkal1, Tnf, Lsm14b, Boll, Ndufa7, Cnot9, Magoh, Mrps34, Mrpl34, Glrx2, Abcf1, Slc30a6, Rwdd1, Niban1, Mrpl16, Sod1, Mrps21, Malsu1, Rps10, Srp9, Rpl21, Iars, Denr, Mrrf, Gpx4, Aph1c, Dhps, Slc30a5, Cpm, Eif2s3x, Mrpl57, Furin, Mrpl24, Slc1a1, Bin1, Mtif3, Mrps2, Shmt2, Bace2, Tars, Cpa3, Eif1ad, Spon1, Ppp1ca, Noct, Dph3, Psenen, Becn1, Mrps5, Gspt1, Rps23, Calr, Tufm, Ggt5, Mrpl3, Ddx1, Tsfm, Eif3b |
| GOBP_OXIDATIVE_PHOSPHORYLATION | 0.0000 | 0.0000 | 0.8513 | 0.5863 | 2.2795 | 130 | Ak4, Nupr1, Atp5j2, Atp5d, Atp5c1, Uqcrb, Myc, Atp5e, Ndufb10, Uqcrfs1, Ndufs7, Ndufa1, Ppif, Cox6a1, Cox7a2l, Ndufa10, Iscu, Ndufb6, Ndufv3, Ghitm, Ndufa6, Atp5j, Tefm, Stoml2, Ndufs6, Ndufa8, Atp5h, Coq9, Atp5k, Ndufa2, Atp5o, Cox7a2, Sdhd, Pink1, Dnajc30, Cox4i1, Chchd10, Ndufv2, Uqcrh, 1700066M21Rik, Atp5pb, Uqcrq, Ndufa12, Ndufs2, Uqcc2, Ndufb1, Rhoa, Ndufb7, Cox5a, Atp5l, Ndufs4, Ndufc1, Ndufv1, Tnf, Ccnb1, Ndufa3, Cox5b, Ndufa7, Ndufs3, Ndufa5, Uqcc3, Ndufb5, Ndufs8, Sdhc, Antkmt, Ndufab1, Shmt2, Ndufb9, Atp5a1, Cox15, Atp5b, Slc25a33, Cdk1, Ndufa4, Uqcr10, Ndufc2, Cox7b2, Snca, Uqcrc1, Nipsnap2, Ndufa9, Ndufs5, Ndufb2 |
| GOBP_AMIDE_BIOSYNTHETIC_PROCESS | 0.0000 | 0.0000 | 1.4173 | 0.4952 | 2.2734 | 744 | Il6, Ang, Btg2, Krt17, Eif2ak3, Thbs1, Eif4ebp1, B4galnt1, Rps5, Rpl26, Abca8b, Rpl7a, Wars1, Rps6, St8sia2, Rps17, Rps12, Pm20d1, Rpl14, Rpl31, Rpsa, Rps26, Rpl36a, Rpl23, Rpl28, Rpl34, Tmed2, Gba, Rpl12, Rps20, Rps16, Rpl6, Rps8, Ormdl3, Rpl37, Lars2, Rpl10a, Rpl37a, Rps29, Mrpl36, Eef2, Rpl23a, Rpl4, Rpl19, Sars, Rpl13, Mrpl52, Rpl35, Eef1d, Cyp1b1, Rps15, Pabpc4, Eef1b2, Rps14, Rps3a1, Tnip1, Rack1, Rpl38, Eef1a1, Rpl22, Eif2s2, Rps13, Rpl27a, Rps3, Eif3h, Rps11, Rps27a, Slc25a1, Akt2, Rpl3, Rplp0, Rpl8, Zfp706, Paip2b, Rpl15, Rps24, Rpl39, Rpl36, Zfp598, Rps9, Eif5b, Rps25, Eif3f, Eif2s1, Acat1, Rpl18a, Rpl7, Ybx3, Rps4x, Mtres1, Mrpl13, Rps2, Rps27l, Ggt6, Rps7, Mars1, Rplp1, Rpl32, Serp1, Rpl5, Pdk2, Mknk1, Rps15a, B3galt4, Rps27, Rps19, Rpl17, Mrpl18, St6galnac4, Cars, Rpl22l1, Rpl27, Rplp2, Rrbp1, Rpl18, Rpl11, Rpl10, Eif3j1, Asah1, Qars, Ccn1, B4galt5, Pabpc1, Hspb1, Rpl13a, Rps21, Rpl30, Hsd17b12, Mrps18c, Dnajc3, Ybx1, Gars, Yars, Rps28, Rpl9, Eif2a, Pdk3, Eif3a, Hacd1, Eif3i, Eif4b, Larp1b, Rpl29, Eif5a, Rps18, Exosc5, Npm1, Vars, Eif2d, P2rx1, Eif3l, Eif3g, Eif1ax, Mrps14, Eif2b5, Asns, Mrps28, Hbs1l, Noa1, Mrps11, Mrps18b, Mrpl35, St3gal3, Samd8, Asah2, Mrpl11, Upf3a, Cdc123, Pemt, Aimp1, Mrps15, Mrpl28, Eif3d, Tyms, Aars, Etf1, Eif6, Mrpl2, Eif4e2, Mrpl43, Mrpl32, Atxn2, Lrrc47, Eef1g, Pdk4, Chac2, Mrpl58, Pink1, Neurl1a, Kars, Nars, Rpl35a, Larp1, Zc3h15, Aimp2, Cops5, Dhfr, Eif3k, Gcdh, Mrpl10, Mrpl23, Mrps24, Igf2bp1, Pkp3, Mrps7, Uqcc2, Pstk, Eif3m, Eif3e, Eif2b1, Rhoa, B4galt3, Dapk3, Mrpl55, Tarbp2, Ppt2, Eif2b4, Piwil2, Ngdn, Gclc, Eif2ak1, Coa3, Tecr, Polr2g, Pa2g4, Akt1, Hagh, Sphk1, Cdkal1, Tnf, Lsm14b, Boll, Ormdl1, Ndufa7, Cnot9, Magoh, Mrps34, Mrpl34, Abcf1, Rwdd1, Niban1, Mrpl16, Mrps21, Malsu1, Rps10, Srp9, Rpl21, Iars, Denr, Mrrf, Dhps, B3galt1, Pdk1, Eif2s3x, Mrpl57, Furin, Smpd1, Mrpl24, Slc1a1, Sgms1, Fa2h, Mtif3, Mrps2, Shmt2, Tars, Acsf3, Eif1ad, Ppp1ca, Ppt1, Noct, Gal3st1, Dph3, Mrps5, Gspt1, Rps23, Calr, Tufm, Ggt5, Mrpl3, Ddx1, Tsfm, Eif3b, Eif3c, Sesn2, Sirt3, Gclm, Elovl1, Dnajc24, Chac1, Nt5c3b, Klhl25 |
| GOBP_NEGATIVE_REGULATION_OF_UBIQUITIN_PROTEIN_LIGASE_ACTIVITY | 0.0000 | 0.0000 | 0.7338 | 0.9264 | 2.2647 | 12 | Rpl23, Rps20, Rpl37, Rps15, Mad2l2, Rps7, Rpl5, Rpl11 |
| GOBP_MITOCHONDRIAL_ATP_SYNTHESIS_COUPLED_PROTON_TRANSPORT | 0.0000 | 0.0001 | 0.6273 | 0.8354 | 2.2564 | 17 | Atp5j2, Atp5d, Atp5c1, Atp5e, Atp5j, Stoml2, Atp5h, Atp5k, Atp5o, Dnajc30, Atp5pb, Atp5l, Antkmt, Atp5a1, Atp5b |
| GOBP_RESPONSE_TO_ENDOPLASMIC_RETICULUM_STRESS | 0.0000 | 0.0000 | 0.9653 | 0.5328 | 2.2485 | 251 | Clu, Xbp1, Thbs4, Eif2ak3, Agr2, Thbs1, Nupr1, Atf3, Rcn3, Derl1, Dnajb12, H13, Cdk5rap3, P4hb, Serinc3, Ern1, Srpx, Creb3, Tmed2, Erp27, Edem1, Ube2j1, Amfr, Cebpb, Rack1, Ufc1, Stc2, Sel1l, Derl3, Selenos, Aup1, Eif2s1, Ubxn1, Serp1, Erlec1, Ubac2, Man1a, Erp44, Edem2, Atf6, Tmbim6, Os9, Canx, Foxred2, Dnajc3, Bok, Sec61b, Gorasp2, Rhbdd1, Rnf121, Edem3, Pigbos1, Ubxn6, Tmem129, Get4, Ddrgk1, Eif2b5, Pmaip1, Rnf186, Scamp5, Anks4b, Tmx1, Pik3r1, Bhlha15, Ube2g2, Tmem259, Herpud1, Grina, Ppp2cb, Man1b1, Vapb, Brsk2, Ern2, Tbl2, Selenok, Uggt1, Pik3r2, Ufm1, Faf1, Rnft1, Cops5, Bcap31, Tmub2, Stub1, Uba5, Manf, Lpcat3, Erp29, Pdia2, Nr1h3, Svip, Ficd, Dnajc10, Nhlrc1, Mbtps1, Nploc4, Fbxo6, Niban1, Trib3, Ube2j2, Bax, Erlin1, Derl2, Sgta, Herpud2, Calr, Atg10, Sdf2l1, Ngly1, Pdia3, Sesn2, Chac1 |
| GOBP_RETROGRADE_VESICLE_MEDIATED_TRANSPORT_GOLGI_TO_ENDOPLASMIC_RETICULUM | 0.0000 | 0.0000 | 0.6750 | 0.6781 | 2.2437 | 49 | Kdelr3, Kdelr2, Copz2, Tmed10, Kdelr1, Sec22b, Cope, Arf4, Copz1, Ergic2, Kif1c, Rint1, Scyl1, Use1, Ergic1, Rer1, Pick1, Ergic3, Lman2, Arcn1, Atp9a, Pitpnb, Arf3, Bnip1, Tmem115, Golph3, Copa, Nbas |
| GOBP_ATP_SYNTHESIS_COUPLED_PROTON_TRANSPORT | 0.0000 | 0.0001 | 0.6105 | 0.7758 | 2.2333 | 23 | Atp5j2, Atp5d, Atp5c1, Atp5e, Atp5j, Stoml2, Atp5h, Atp5k, Atp5g3, Atp5o, Dnajc30, Atp5g1, Atp5pb, Atp5g2, Atp5l, Antkmt, Atp5a1, Atp5b |
| GOBP_MITOCHONDRIAL_TRANSPORT | 0.0000 | 0.0000 | 0.8634 | 0.5431 | 2.2208 | 189 | Atpif1, Dnajc19, Sh3glb1, Nol3, Eya2, Rhou, Atp5j2, Atp5d, Atp5c1, Lman1, Mpv17l, Atp5e, Grpel1, Ndufa13, Dnlz, Immp2l, Slc25a5, Bcs1l, Ppif, Arih2, Maip1, Bap1, Bok, Vdac2, Timm23, Slc35f6, Slc25a46, Timm17b, Atp5j, Mff, Pmaip1, Parl, Romo1, Tomm20, Bcl2l2, Timm22, Stoml2, Grpel2, Bnip3l, Atp5h, Psen1, Atp5k, Vps11, Pmpcb, Timm13, Htra2, Oxa1l, Atp5o, Tomm70a, Pink1, Tomm5, Dnajc30, Chchd10, Tomm22, Bcap31, Atg13, Rhot2, Chchd4, Atp5pb, Timm8b, Tomm40l, Atp5l, Gclc, Pdcd5, Sae1, Samm50, Ube2j2, Bax, Tomm7, Antkmt, Slc25a10, Stard3, Cnp, Timm44, Atp5a1, Atp5b, Timm8a1, Ubl5 |
| GOBP_PEPTIDYL_ASPARAGINE_MODIFICATION | 0.0000 | 0.0001 | 0.6105 | 0.7673 | 2.2172 | 24 | Fut8, Rpn2, Stt3a, Alg5, Ube2j1, Magt1, Derl3, Ddost, Ostc, Ost4, Uggt1, Dad1, Rpn1, Hif1an, Tusc3, Mgat2, Nudt14 |
| GOBP_GLYCOSYLATION | 0.0000 | 0.0000 | 0.9101 | 0.5324 | 2.2146 | 231 | Galnt7, Slc39a8, Galntl6, B4galnt1, Extl3, Mgat4a, Nans, Fut8, Tmem258, Rpn2, Galnt17, St8sia2, Stt3a, Alg5, Gba, Krtcap2, Vegfb, Ube2j1, Magt1, Derl3, Dpagt1, Ddost, Fut4, Alg2, Slc35c1, Rxylt1, Serp1, Ostc, Alg11, Mgat4b, Nagpa, B3galt4, Galnt3, Tmem59, St6galnac4, Gcnt4, Man1a, Man2a2, Fut2, B4galt5, Plod1, Dpy19l1, Mlec, Alg3, Pomgnt1, Nus1, B3gnt9, Alg9, Slc35c2, Ost4, Edem3, Ramp1, Entpd5, C1galt1c1, Gcnt1, B3galt6, Man1c1, B4galt2, Fuom, Pomt2, St3gal3, Alg1, Cln5, Man1b1, Psen1, Srd5a3, Pofut2, Uggt1, Mgat3, Dad1, Galnt1, Rpn1, Cog1, Mt3, Pmm2, Extl1, Plod2, Mogs, Gmppb, St6galnac2, B4galt3, Pomgnt2, Rft1, B3gnt8, Galnt2, Large1, Cog6, Dpm2, B4galt1, B3galnt2, B3galt1, Pmm1, Plod3, Galnt4, Mpdu1, Gal3st1, Tusc3, B3gat3, B3gnt2, Large2, Dhdds, B4galt7, Crppa, Mgat2, Galnt6, Nudt14, Gfpt1 |
| GOBP_PROTEIN_FOLDING | 0.0000 | 0.0000 | 0.8513 | 0.5421 | 2.2086 | 186 | Clu, Ppic, Sil1, Ppib, Dnajb12, P4hb, Dnajc19, Cct4, Ptges3l, Erp27, Tcp1, Prdx4, P3h1, Fkbp9, Qsox1, Tor2a, Sdf2, Pdia6, Lman1, Grpel1, Fkbp2, Dnlz, Fkbp11, Ppif, Cct5, Wdr83os, Erp44, Atf6, Selenof, Qsox2, Hspb1, Tbca, Canx, Crtap, Dnajc3, Hspe1, Entpd5, Ero1b, Tor1b, Pfdn5, Dnajc2, Dnajb11, Grpel2, Dnajc21, Pofut2, Uggt1, Grn, Cct7, St13, Pfdn1, Chchd4, Fkbp8, B2m, Erp29, Mogs, Pdia2, Dnajc10, Pdcl3, Cct6b, Dnaja2, Pdcd5, Dnajc25, Mesd, Hspbp1, Hspa13, Fkbp5, Mpdu1, Pfdn2, Dnajc5, Dnajc7, Calr, Sdf2l1, Hspa9, Ngly1, Pdia3, Cdc37, Dnajc24 |
| GOBP_RIBOSOMAL_SMALL_SUBUNIT_ASSEMBLY | 0.0000 | 0.0003 | 0.5756 | 0.8087 | 2.1842 | 17 | Rps5, Rpsa, Rps15, Rps14, Rpl38, Rps27l, Rps27, Rps19, Rps28, Mrps11, Abt1, Mrps7 |
| GOBP_TRANSLATIONAL_INITIATION | 0.0000 | 0.0000 | 0.7195 | 0.5688 | 2.1832 | 113 | Eif2ak3, Eif4ebp1, Rps5, Rps17, Tmed2, Rps3a1, Eif2s2, Rps3, Eif3h, Paip2b, Eif5b, Eif3f, Eif2s1, Eif3j1, Hspb1, Rpl13a, Dnajc3, Eif2a, Eif3a, Eif3i, Eif4b, Npm1, Eif2d, Eif3l, Eif3g, Eif1ax, Eif2b5, Cdc123, Eif3d, Eif6, Eif4e2, Larp1, Cops5, Eif3k, Eif3m, Eif3e, Eif2b1, Eif2b4, Eif2ak1, Polr2g, Tnf, Boll, Denr, Eif2s3x, Mtif3, Eif1ad, Ppp1ca, Ddx1, Eif3b, Eif3c, Klhl25, Atf4, Eif3j2 |
| GOBP_RESPONSE_TO_TOPOLOGICALLY_INCORRECT_PROTEIN | 0.0000 | 0.0000 | 0.8012 | 0.5483 | 2.1747 | 155 | Clu, Xbp1, Thbs4, Eif2ak3, Agr2, Thbs1, Atf3, Derl1, Dnajb12, Cdk5rap3, Ern1, Creb3, Tmed2, Erp27, Edem1, Amfr, Tram1, Rack1, Stc2, Derl3, Selenos, Aup1, Eif2s1, Serp1, Erlec1, Erp44, Edem2, Atf6, Tmbim6, Hspb1, Os9, Dnajc3, Bok, Hspe1, Rhbdd1, Edem3, Pigbos1, Tmem129, Ddrgk1, Tor1b, Serpinh1, Pik3r1, Bhlha15, Herpud1, Vapb, Ern2, Tbl2, Uggt1, Cops5, Stub1, Manf, Ficd, Mbtps1, Fbxo6, Optn, Ube2j2, Bax, Hspa13, Derl2, Herpud2, Sdf2l1, Hspa9, Ngly1, Chac1, Atf4, Dnajb9, Rnf5, Tmem33, Dnajb1 |
| GOBP_POSTTRANSLATIONAL_PROTEIN_TARGETING_TO_ENDOPLASMIC_RETICULUM_MEMBRANE | 0.0000 | 0.0006 | 0.5573 | 0.8422 | 2.1740 | 14 | Sec61a1, Sec61g, Sec63, Chmp4b, Sec62, Sec61b, Get3, Get4 |
| GOBP_OLIGOSACCHARIDE_LIPID_INTERMEDIATE_BIOSYNTHETIC_PROCESS | 0.0000 | 0.0002 | 0.6105 | 0.8006 | 2.1671 | 18 | Dpagt1, Alg2, Alg11, Alg3, Nus1, Alg9, Alg1, Srd5a3, Rft1, Mpdu1, Dhdds, Alg10b, Alg6, Dolk |
| GOBP_PROTEIN_IMPORT_INTO_MITOCHONDRIAL_MATRIX | 0.0000 | 0.0003 | 0.5933 | 0.7820 | 2.1649 | 19 | Dnajc19, Grpel1, Dnlz, Timm23, Timm17b, Romo1, Tomm20, Grpel2, Tomm70a, Tomm40l, Tomm7, Timm44 |
| GOBP_RIBOSOMAL_SMALL_SUBUNIT_BIOGENESIS | 0.0000 | 0.0000 | 0.6750 | 0.6068 | 2.1624 | 71 | Rps5, Rps6, Rps17, Rpsa, Rps16, Rps8, Rps15, Rps14, Rpl38, Rps24, Rps25, Utp23, Rps27l, Rps7, Rps27, Rps19, Rps21, Rps28, Riok3, Npm1, Mrps11, Rrs1, Abt1, Utp3, Lsm6, Nop9, Mrps7, Ngdn, Utp4, Fam207a, Nob1 |
| GOBP_AEROBIC_RESPIRATION | 0.0000 | 0.0000 | 0.8140 | 0.5336 | 2.1573 | 176 | Ak4, Nupr1, Atp5j2, Atp5d, Atp5c1, Uqcrb, Myc, Atp5e, Ndufb10, Uqcrfs1, Ndufs7, Ndufa1, Ppif, Cox6a1, Cox7a2l, Ndufa10, Iscu, Ndufb6, Mdh2, Ndufv3, Ghitm, Ndufa6, Atp5j, Tefm, Suclg1, Mtfr1l, Stoml2, Ndufs6, Ndufa8, Atp5h, Idh3b, Coq9, Atp5k, Nop53, Ndufa2, Oxa1l, Atp5o, Cox7a2, Sdhd, Pink1, Dnajc30, Cox4i1, Chchd10, Ndufv2, Uqcrh, Idh1, 1700066M21Rik, Atp5pb, Uqcrq, Ndufa12, Cs, Ndufs2, Uqcc2, Ndufb1, Rhoa, Ndufb7, Cox5a, Atp5l, Ndufs4, Ndufc1, Ndufv1, Tnf, Ccnb1, Ndufa3, Cox5b, Ndufa7, Surf1, Ndufs3, Ndufa5, Uqcc3, Ndufb5, Ndufs8, Sdhc, Antkmt, Ndufab1, Shmt2, Ndufb9, Idh3a, Atp5a1, Cox15, Dlst, Atp5b, Sdhb, Slc25a33, Sirt3, Cdk1, Nnt, Fh1, Mrps36, Ndufa4, Uqcr10, Bnip3, Ndufc2, Pdha1, Cox7b2, Snca, Uqcrc1, Nipsnap2, Ndufa9, Ndufs5, Ndufb2 |
| GOBP_COTRANSLATIONAL_PROTEIN_TARGETING_TO_MEMBRANE | 0.0000 | 0.0003 | 0.5756 | 0.7612 | 2.1410 | 21 | Srpr, Sil1, Sec61a1, Ssr3, Tram1, Sec63, Sec62, Srp14, Ssr1, Sec61b, Ssr2, Srp68, Srp72, Srp19, Srp9, Tram2, Tram1l1, Zfand2b |
| GOBP_CELLULAR_AMIDE_METABOLIC_PROCESS | 0.0000 | 0.0000 | 1.4246 | 0.4578 | 2.1401 | 1018 | Igf1, Clu, Ctsl, Il6, Ang, Btg2, Krt17, Eif2ak3, Thbs1, Eif4ebp1, B4galnt1, Rps5, Cpxm1, Rpl26, Cpxm2, Smpdl3a, Abca8b, Rpl7a, Wars1, H13, Hexa, Tmed10, Rps6, St8sia2, Rps17, Abcg1, Rps12, Pm20d1, Rpl14, Rpl31, Rpsa, Rps26, Rpl36a, Cpn1, Ctns, Rpl23, Rpl28, Rpl34, Tmed2, Gba, Rpl12, Rps20, Rps16, Rpl6, Rps8, Ormdl3, Rpl37, Spcs1, Trhde, Lars2, Rpl10a, Rpl37a, Rps29, Mrpl36, Eef2, Rpl23a, Rpl4, Rpl19, Sars, Rpl13, Mrpl52, Rpl35, Eef1d, Cyp1b1, Rps15, Pabpc4, Eef1b2, Rps14, Anpep, Rps3a1, Tnip1, Rack1, Rpl38, Xpnpep1, Eef1a1, Rpl22, Eif2s2, Rps13, Rpl27a, Rps3, Eif3h, Cel, Ethe1, Rps11, Rps27a, Slc25a1, Akt2, Rpl3, Rplp0, Rpl8, Zfp706, Paip2b, Rpl15, Rps24, Rpl39, Rpl36, Zfp598, Rps9, Yipf5, Eif5b, Rtn3, Rps25, Eif3f, Eif2s1, Acat1, Rpl18a, Rpl7, Ybx3, Rps4x, Mtres1, Mrpl13, Rps2, Rps27l, Ggt6, Rps7, Mars1, Rplp1, Rpl32, Serp1, Rpl5, Mthfd2, Pdk2, Gsto1, Mknk1, Rps15a, B3galt4, Rps27, Rps19, Dpep1, Spcs3, Dnpep, Rpl17, Mrpl18, St6galnac4, Nnmt, Cars, Rpl22l1, Spcs2, Rpl27, Rplp2, Rrbp1, Rpl18, Rpl11, Rpl10, Psen2, Eif3j1, Asah1, Qars, Pipox, Ccn1, B4galt5, Pabpc1, Hspb1, Rpl13a, Rps21, Rpl30, Hsd17b12, Mrps18c, Ctsz, Sec11c, Dnajc3, Ybx1, Gars, Yars, Rps28, Rpl9, Eif2a, Pdk3, Eif3a, Hacd1, Cln6, Eif3i, Galc, Cpq, Eif4b, Slc2a13, Larp1b, Naga, Gstp1, Rpl29, Eif5a, Rps18, Exosc5, Npm1, Vars, Gstm2, Eif2d, P2rx1, Eif3l, Eif3g, Eif1ax, Mrps14, Eif2b5, Asns, Mrps28, Gnpda1, Nat8f1, Ptges2, Parl, Hbs1l, Noa1, Mrps11, Acot7, Mrps18b, Mrpl35, Acsm3, St3gal3, Samd8, Asah2, Mrpl11, Upf3a, Acot11, Cln5, Cdc123, Pemt, Psen1, Gstm5, Aimp1, Nudt8, Mrps15, Atp6ap2, Mrpl28, Sgpl1, Eif3d, Tyms, Aars, Etf1, Eif6, Mrpl2, Eif4e2, Mrpl43, Vnn1, Htra2, Mrpl32, Atxn2, Lrrc47, Aebp1, Mgat3, Eef1g, Pdk4, Chac2, Slc19a1, Carnmt1, Mrpl58, Pink1, Lta4h, Neurl1a, Kars, Nars, Rpl35a, Larp1, Zc3h15, Aimp2, Cops5, Dhfr, Eif3k, Pcsk6, Idh1, Gcdh, Sec11a, Mrpl10, Mrpl23, Mrps24, Igf2bp1, Pkp3, Mrps7, Tm9sf2, Uqcc2, Pstk, Eif3m, Eif3e, Eif2b1, Tpp1, Rhoa, B4galt3, Dapk3, Mrpl55, Tarbp2, Ppt2, Fpgs, Eif2b4, Vnn3, Piwil2, Ngdn, Gclc, Eif2ak1, Coa3, Tecr, Polr2g, Pa2g4, Akt1, Hagh, Sphk1, Cdkal1, Tnf, Lsm14b, Mvk, Boll, Ormdl1, Ndufa7, Cnot9, Magoh, Mrps34, Mrpl34, Glrx2, Abcf1, Slc30a6, Rwdd1, Niban1, Mrpl16, Sod1, Mrps21, Malsu1, Rps10, Srp9, Rpl21, Iars, Denr, Mrrf, Mvd, Gpx4, Aph1c, Dhps, Slc30a5, B3galt1, Cpm, Pdk1, Eif2s3x, Cyp2c65, Mrpl57, Aldh1l2, Furin, Smpd1, Mrpl24, Slc1a1, Sgms1, Bin1, Fa2h, Mtif3, Mrps2, Neu1, Shmt2, Bace2, Tars, Acsf3, Cpa3, Eif1ad, Spon1, Ppp1ca, Ppt1, Noct, Gal3st1, Dph3, Dlst, Psenen, Becn1, Mrps5, Gspt1, Rps23, Pgam1, Calr, Tufm, Ggt5, Mrpl3, Ddx1, Tsfm, Eif3b, Amdhd2, Eif3c, Sesn2, Sirt3, Gclm, Elovl1, Dnajc24, Acot9, Chac1, Nt5c3b, Klhl25 |
| GOBP_RIBOSOMAL_LARGE_SUBUNIT_BIOGENESIS | 0.0000 | 0.0000 | 0.6594 | 0.6037 | 2.1328 | 69 | Rpl26, Rpl7a, Rpl14, Rpl6, Rpl10a, Rpl23a, Rpl35, Rpl38, Rpl3, Rplp0, Nsa2, Rpl7, Rpl5, Gtf3a, Rpl11, Rpl10, Nhp2, Npm1, Mrpl20, Nop53, Rrs1, Eif6, Rpl35a, Nle1, Znhit6, Pes1, Rsl1d1, Malsu1, Mak16, Rpf1 |
| GOBP_ENDOPLASMIC_RETICULUM_UNFOLDED_PROTEIN_RESPONSE | 0.0000 | 0.0001 | 0.6273 | 0.5925 | 2.1236 | 74 | Xbp1, Eif2ak3, Agr2, Atf3, Derl1, Cdk5rap3, Ern1, Creb3, Tmed2, Amfr, Rack1, Stc2, Derl3, Selenos, Eif2s1, Serp1, Erlec1, Atf6, Os9, Bok, Pigbos1, Ddrgk1, Pik3r1, Bhlha15, Herpud1, Vapb, Ern2, Tbl2, Cops5, Stub1, Ficd, Mbtps1 |
| GOBP_GLYCOPROTEIN_METABOLIC_PROCESS | 0.0000 | 0.0000 | 0.9326 | 0.4864 | 2.1160 | 352 | Igf1, Ctsl, Bgn, Galnt7, Slc39a8, Phlda1, Galntl6, Extl3, Chpf, Mgat4a, Fut8, Tmem258, Cst3, Rpn2, Itm2a, Galnt17, St8sia2, Egflam, Stt3a, Alg5, Krtcap2, Vegfb, Edem1, Ube2j1, Magt1, Amfr, Adamts12, Prkcsh, Itm2c, Tnip1, Derl3, Dpagt1, Ddost, Fut4, Alg2, Spock2, Slc35c1, Rxylt1, Serp1, Ostc, Alg11, Mgat4b, Nagpa, B3galt4, Galnt3, Tmem59, St6galnac4, Gcnt4, Man1a, Man2a2, Fut2, Col11a1, Erp44, B4galt5, Edem2, Plod1, Dpy19l1, Mlec, Alg3, Pomgnt1, Nus1, B3gnt9, Alg9, Slc35c2, Ost4, Edem3, Ramp1, Soat1, Entpd5, C1galt1c1, Gcnt1, Aga, B3galt6, Man1c1, Dcn, Bmpr1b, B4galt2, Pomt2, St3gal3, Alg1, Man1b1, Psen1, Gnptab, Srd5a3, Pofut2, Uggt1, Pxylp1, Man2b1, Necab1, Mgat3, Dad1, Galnt1, Rpn1, Hs3st3b1, Pcsk6, Mt3, Pmm2, Aatf, Extl1, Plod2, Tm9sf2, Mogs, Gmppb, St6galnac2, B4galt3, Xylt1, Pomgnt2, Rft1, Adamts7, B3gnt8, Gpc1, Galnt2, Large1, Ganab, Fbxo6, Cela1, Slc2a10, Rab1a, Dse, Dpm2, B4galt1, B3galnt2, B3galt1, Pmm1, Plod3, Porcn, Bace2, Galnt4, Mpdu1, Golph3, Chsy3, Sulf1, Gal3st1, Tusc3, Ust, B3gat3, B3gnt2, Large2, Dhdds, B4galt7, Ngly1, Crppa, Mgat2, Galnt6, Hs2st1, Nudt14 |
| GOBP_GOLGI_VESICLE_TRANSPORT | 0.0000 | 0.0000 | 0.8871 | 0.4948 | 2.1154 | 282 | Lamp1, Kdelr3, Sec16b, Wipi1, Tmed3, Arfgap3, Kdelr2, Copz2, Tmed10, Ykt6, Kdelr1, Tmed2, Surf4, Scap, Sec22b, Rack1, Ap3s1, Golga7, Arl1, Lman1, Cope, Spast, Yipf5, Yif1a, Rab29, Arf4, Nkd2, Copz1, Cyth3, Gga2, Scamp1, Bet1, Yif1b, Ier3ip1, Rabif, Rab2a, Ergic2, Vti1b, Kif1c, Rab26, Sec13, Tfg, Rab8a, Rint1, Scyl1, Tmed4, Mia3, Use1, Coro7, Ap2a1, Napa, Tmed6, Gosr2, Ergic1, Tmed5, Osbpl5, Tex261, Rer1, Preb, Tmed9, Vapb, Pdcd6, Sar1a, Pick1, Ergic3, Mia2, Snx8, Lman2, Snx3, Arcn1, Sec31a, Phaf1, Cog1, Gga1, Bcap31, Ap4m1, Atp9a, Vamp4, Rab34, Copg1, Tbc1d20, Lypla1, Insig1, Sys1, Sec22c, Trappc6a, Pitpnb, Arf3, Golga5, Rab31, Rab1a, Cog6, Optn, Trappc1, Bnip1, Trappc3, Tmem115, Kif16b, Stx5a, Golph3, Copa, Nbas, Rab7b, Sec23a, Sar1b, Golga4, Vti1a, Sec24a, Scamp2, Snap23, Ppp6c, Copb1, Pkdcc, Arf5 |
| GOBP_COLLAGEN_METABOLIC_PROCESS | 0.0000 | 0.0000 | 0.6594 | 0.5675 | 2.1082 | 92 | Ctsl, Try5, Il6, Prss3, Cygb, Gm10334, Ctsk, Ccn2, Prss2, F2r, Rcn3, F2, Cst3, Prss1, Mrc2, Bmp4, Col1a1, P3h1, Emilin1, Fap, Col1a2, Mfap4, P3h3, Mmp7, Mmp2, Serpinh1, Mmp19, Errfi1, Mmp14, Runx1, Gm5771, P3h4, Try10, Itgb1, Furin, Pdgfrb, Plod3, Ctsb, Mmp16, Col5a1, Tgfb1 |
| GOBP_SKIN_MORPHOGENESIS | 0.0000 | 0.0003 | 0.5933 | 0.8798 | 2.1078 | 11 | Gba, Col1a1, Itgb4, Col1a2, Psen1, Errfi1, Trp63 |
| GOBP_INTRACELLULAR_PROTEIN_TRANSMEMBRANE_TRANSPORT | 0.0000 | 0.0002 | 0.6105 | 0.6336 | 2.1070 | 51 | Sec61a1, Dnajc19, Sec61g, Tram1, Sec63, Akt2, Grpel1, Dnlz, Sec62, Sec61b, Timm23, Timm17b, Romo1, Tomm20, Grpel2, Pex5, Pex7, Tomm70a, Chchd4, Tomm40l, Tomm7, Timm44, Tram2, Rtn2, Tram1l1, Zfand2b, Pex14 |
| GOBP_ORGANONITROGEN_COMPOUND_BIOSYNTHETIC_PROCESS | 0.0000 | 0.0000 | 1.6407 | 0.4423 | 2.1055 | 1513 | Igf1, Slc6a9, Il6, Galnt7, Ang, Slc39a8, Fabp5, Btg2, Krt17, Gls2, Prpsap1, Eif2ak3, Ak4, Thbs1, Phlda1, Galntl6, Eif4ebp1, B4galnt1, Extl3, Chpf, Wipi1, Atpif1, Rps5, Ddc, Mgat4a, Rpl26, Abca8b, Rpl7a, Fut8, Wars1, Tmem258, Rpn2, Sephs2, Hexa, Itm2a, Rps6, Serinc3, Galnt17, Chst2, St8sia2, Rps17, Pla2g4a, Rps12, Stt3a, Rpl14, Rpl31, Rpsa, Alg5, Rps26, Rpl36a, Pcbd1, Rpl23, Rpl28, Rpl34, Tmed2, Gba, Rpl12, Rps20, Krtcap2, Rps16, Rpl6, Vegfb, Rps8, Mthfd1l, Ube2j1, Ormdl3, Rpl37, Magt1, Csnk1g2, Lars2, Rpl10a, Rpl37a, Rps29, Mrpl36, Eef2, Rpl23a, Rpl4, Rpl19, Sars, Rpl13, Mrpl52, Rpl35, Eef1d, Cyp1b1, Rps15, Ak3, Pabpc4, Atp5j2, Eef1b2, Itm2c, Rps14, Atp5d, Rps3a1, Tnip1, Rack1, Rpl38, Eef1a1, Rpl22, Atp5c1, Eif2s2, Rps13, Rpl27a, Rps3, Nme6, Eif3h, Golga7, Apob, Rps11, Rps27a, Slc25a1, Spta1, Pigf, Derl3, Akt2, Nme1, Dpagt1, Rpl3, Rplp0, Rpl8, Ddost, Zfp706, Paip2b, Rpl15, Myc, Rps24, Rpl39, Fut4, Alg2, Aldh18a1, Rpl36, Guk1, Zfp598, Rps9, Atp5e, Eif5b, Rps25, Eif3f, Eif2s1, Adcy7, Zdhhc3, Acat1, Rpl18a, Rpl7, Ybx3, Rps4x, Slc35c1, Rxylt1, Mtres1, Mrpl13, Rps2, Rps27l, Pycr1, Ckb, Ggt6, Rps7, Mars1, Rplp1, Rpl32, Serp1, Rpl5, Ostc, Alg11, Pdk2, Mgat4b, Mknk1, Nagpa, Rps15a, B3galt4, Sgpp1, Rps27, Rps19, Galnt3, Rpl17, Tmem59, Mrpl18, St6galnac4, Nnmt, Cars, Rpl22l1, Gcnt4, Man1a, Rpl27, Rplp2, Rrbp1, Rpl18, Man2a2, Fut2, Rpl11, Rpl10, Eif3j1, Asah1, Qars, Ccn1, B4galt5, Pabpc1, Smox, Pigt, Ckmt1, Plod1, Dpy19l1, Mlec, Chpt1, Alg3, Hspb1, Pomgnt1, Rpl13a, Rps21, Pid1, Rpl30, Nus1, B3gnt9, Hsd17b12, Mrps18c, Asnsd1, Dnajc3, Ybx1, Gars, Yars, Rps28, Rpl9, Urod, Has1, Cept1, Eif2a, Alg9, Pdk3, Eif3a, Hacd1, Slc35b2, Slc35c2, Ost4, Eif3i, Edem3, Hs3st6, Eif4b, Larp1b, Hhat, Ramp1, Gstp1, Soat1, Rpl29, Eif5a, Rps18, Psat1, Entpd5, Exosc5, Npm1, Vars, Gstm2, Phgdh, Zdhhc12, Eif2d, Ap2a1, P2rx1, C1galt1c1, Hpca, Eif3l, Gcnt1, Atp5j, Eif3g, B3galt6, Eif1ax, Mrps14, Eif2b5, Asns, Mrps28, Man1c1, Akr1a1, Pigq, Hbs1l, Abcb6, Bmpr1b, B4galt2, Noa1, Mocs2, Gpaa1, Pomt2, Stoml2, Mrps11, Acot7, Mrps18b, Mrpl35, Paox, Glud1, St3gal3, Alg1, Samd8, Iba57, Asah2, Mrpl11, Upf3a, Atp5h, Cdc123, Pemt, Man1b1, Psen1, Atp5k, Spns2, Srm, Aimp1, Mrps15, Mrpl28, Abat, Sgpl1, Eif3d, Tyms, Aars, Etf1, Eif6, Mrpl2, Eif4e2, Mrpl43, Srd5a3, Clip3, Mrpl32, Atp5g3, Selenok, Pofut2, Slc25a39, Uggt1, Atxn2, Pxylp1, Lrrc47, Necab1, Atp5o, Mgat3, Odc1, Eef1g, Dad1, Tyw1, Pdk4, Chac2, Galnt1, Rfk, Rpn1, Mrpl58, Pink1, Mocs3, Cmpk1, Neurl1a, Dnajc30, Hs3st3b1, Kars, Nars, Rpl35a, Atp5g1, Larp1, Slc1a3, Zc3h15, Aimp2, Cops5, Dhfr, Eif3k, Mt3, Pmm2, Atg13, Atg7, Aatf, Hprt, Extl1, Atg5, Gcdh, Zdhhc6, Atp5pb, Mrpl10, Mrpl23, Plod2, Mrps24, Igf2bp1, Bcat2, Pkp3, Uck2, Lpcat3, Mrps7, Nmnat3, Tm9sf2, Uqcc2, Pstk, Eif3m, Eif3e, Qprt, Eif2b1, Nfs1, Mogs, Gmppb, St6galnac2, Rhoa, B4galt3, Dapk3, Xylt1, Mrpl55, Pomgnt2, Atp5g2, Tarbp2, Rft1, Wipi2, Ppt2, Atp5l, Svip, Fpgs, Eif2b4, Piwil2, Ngdn, Pigo, Gclc, Eif2ak1, Coa3, Tecr, Polr2g, Pa2g4, B3gnt8, Akt1, Hagh, Sphk1, Nos1, Cdkal1, Tnf, Lsm14b, Boll, Galnt2, Umps, Ormdl1, Ndufa7, Large1, Glul, Ppm1a, Cnot9, Magoh, Mrps34, Mrpl34, Abcf1, Rwdd1, Niban1, Pgap3, Mrpl16, Slc2a10, Mrps21, Malsu1, Rps10, Tmem14c, Srp9, Dse, Dpm2, B4galt1, Rpl21, Uqcc3, Iars, Denr, Mrrf, B3galnt2, Dhps, Nme4, B3galt1, Oaz1, Pmm1, Pdk1, Nadsyn1, Eif2s3x, Antkmt, Mrpl57, Pigw, Furin, Pigs, Pdgfrb, Plod3, Smpd1, Mrpl24, Egf, Porcn, Slc1a1, Sgms1, Fa2h, Mtif3, Mrps2, Ampd2, Shmt2, Bace2, Tars, Galnt4, Acsf3, Mpdu1, Eif1ad, Golph3, Chsy3, Zdhhc7, Pigu, Ppp1ca, Slc11a2, Ppt1, Atp5a1, Noct, Gal3st1, Dph3, Tusc3, Ust, Tgfb1, Cox15, B3gat3, Atp5b, Mrps5, Gspt1, Gucy1b1, Rps23, B3gnt2, Large2, Calr, Tufm, Ggt5, Dhdds, Mrpl3, Ddx1, Atg10, Tsfm, Eif3b, Atic, B4galt7, Slc44a3, Eif3c, Rab38, Crppa, Sesn2, Sirt3, Gclm, Mgat2, Elovl1, Dnajc24, Galnt6, Hs2st1, Nudt14, Chac1, Nt5c3b, Mtap, Klhl25 |
| GOBP_PROTEIN_TRANSMEMBRANE_TRANSPORT | 0.0000 | 0.0002 | 0.6105 | 0.6033 | 2.0982 | 62 | Sec61a1, Tmed10, Dnajc19, Sec61g, Tram1, Sec63, Ednra, Akt2, Grpel1, Dnlz, Sec62, Sec61b, Timm23, Timm17b, Romo1, Tomm20, Timm22, Grpel2, Pex5, Pex7, Tomm70a, Tomm22, Chchd4, Ap4m1, Tomm40l |
| GOBP_PROTEIN_INSERTION_INTO_ER_MEMBRANE | 0.0001 | 0.0012 | 0.5384 | 0.7281 | 2.0959 | 23 | Sec61a1, Emc7, Tram1, Wdr83os, Emc10, Mmgt1, Emc4, Emc3, Get3, Get4, Caml, Sgta, Tram2, Emc6, Tram1l1 |
| GOBP_REGULATION_OF_PROTEIN_EXIT_FROM_ENDOPLASMIC_RETICULUM | 0.0001 | 0.0010 | 0.5573 | 0.7126 | 2.0947 | 25 | Sec16b, Tmem30b, Edem1, Ube2j1, Derl3, Erlec1, Ubac2, Edem2, Os9, Tm9sf4, Ube2g2, Brsk2, Sar1a, Bcap31, Svip, Insig1, Derl2, Sar1b, Tmem30a |
| GOBP_SRP_DEPENDENT_COTRANSLATIONAL_PROTEIN_TARGETING_TO_MEMBRANE | 0.0001 | 0.0012 | 0.5384 | 0.7966 | 2.0905 | 15 | Sec61a1, Ssr3, Tram1, Sec63, Srp14, Sec61b, Srp68, Srp72, Srp19, Srp9, Tram2, Tram1l1, Zfand2b |
| GOBP_ERAD_PATHWAY | 0.0000 | 0.0000 | 0.6594 | 0.5500 | 2.0898 | 107 | Rcn3, Derl1, Dnajb12, H13, Edem1, Ube2j1, Amfr, Sel1l, Derl3, Selenos, Aup1, Ubxn1, Erlec1, Ubac2, Man1a, Edem2, Os9, Canx, Foxred2, Sec61b, Rhbdd1, Rnf121, Edem3, Ubxn6, Tmem129, Get4, Ube2g2, Tmem259, Herpud1, Man1b1, Brsk2, Uggt1, Faf1, Rnft1, Bcap31, Tmub2, Stub1, Svip, Dnajc10, Nploc4, Fbxo6, Ube2j2, Erlin1, Derl2, Sgta, Calr, Sdf2l1, Ngly1 |
| GOBP_PROTEIN_TARGETING_TO_MITOCHONDRION | 0.0000 | 0.0000 | 0.6750 | 0.5536 | 2.0803 | 99 | Atpif1, Dnajc19, Sh3glb1, Rhou, Lman1, Grpel1, Ndufa13, Dnlz, Immp2l, Arih2, Bap1, Timm23, Timm17b, Mff, Parl, Romo1, Tomm20, Timm22, Grpel2, Bnip3l, Vps11, Pmpcb, Timm13, Htra2, Tomm70a, Pink1, Tomm5, Tomm22, Bcap31, Atg13, Chchd4, Timm8b, Tomm40l, Pdcd5, Sae1, Samm50, Ube2j2, Tomm7, Timm44, Timm8a1, Ubl5 |
| GOBP_RIBOSOME_BIOGENESIS | 0.0000 | 0.0000 | 0.8753 | 0.4852 | 2.0759 | 290 | Rps5, Rpl26, Rpl7a, Rps6, Rps17, Rpl14, Imp3, Rpsa, Rps16, Rpl6, Rps8, Rpl10a, Mrpl36, Rpl23a, Rpl35, Rps15, Rps14, Rpl38, Rpl3, Rplp0, Rps24, Rps25, Nsa2, Rpl7, Utp23, Rps27l, Rps7, Rpl5, Rps27, Gtf3a, Rps19, Rpl27, Rpl11, Rpl10, Nhp2, Rps21, Rps28, Eif2a, Ddx54, Pih1d1, Ddx49, Riok3, Rbis, Exosc5, Npm1, Mrpl20, Mrps11, Mphosph10, Lyar, Nsun4, Nop53, Rrs1, C1d, Eif6, Abt1, Nop10, Utp3, Lsm6, Eri3, Rpl35a, Nop9, Pop5, Imp4, Aatf, Nle1, Lsg1, Mrps7, Znhit6, Rrp8, Ngdn, Rbfa, Pa2g4, Pes1, Utp14a, Rsl1d1, Isg20, Pwp1, Glul, Malsu1, Wdr55, Utp4, Fam207a, Mak16, Rpf1, Mterf3, Wdr43, Mrps2, Nob1, Rrp9, Wdr74, Frg1, Tsr3, Trmt112, Tsr1, Rpusd3, C1qbp, Ppan, Exosc2, Sart1, Cul4a, Gtf2h5, Nol10, Bop1, Gtpbp4, Exosc4, Srfbp1, Mphosph6, Tfb2m, Utp15, Las1l, Ltv1, Dhx29, Tbl3, Wdr12, Ybey, Ddx56, Rpl7l1, Rrn3 |
| GOBP_COPII_COATED_VESICLE_BUDDING | 0.0001 | 0.0012 | 0.5384 | 0.6752 | 2.0697 | 31 | Sec16b, Tmed10, Tmed2, Scap, Sec13, Tfg, Mia3, Preb, Vapb, Pdcd6, Sar1a, Sec31a, Tbc1d20, Insig1, Rab1a, Sec23a, Sar1b, Sec24a, Ppp6c |
| GOBP_PROTEIN_LOCALIZATION_TO_MITOCHONDRION | 0.0000 | 0.0000 | 0.6750 | 0.5329 | 2.0683 | 123 | Atpif1, Dnajc19, Sh3glb1, Rhou, Lman1, Grpel1, Ndufa13, Dnlz, Immp2l, Bcs1l, Arih2, Maip1, Bap1, Timm23, Timm17b, Mff, Rnf186, Parl, Romo1, Tomm20, Timm22, Grpel2, Bnip3l, Vps11, Pmpcb, Timm13, Htra2, Oxa1l, Tomm70a, Pink1, Tomm5, Tomm22, Bcap31, Mtch2, Atg13, Chchd4, Timm8b, Tomm40l, Akt1, Pdcd5, Sae1, Ap3b1, Samm50, Ube2j2, Bax, Tomm7, Timm44, Marchf5, Timm8a1, Ubl5 |
