# Supplementary material for: RalGAP complexes control secretion and primary cilia in pancreatic disease
Source: Life Sci Alliance. 2025 Jun 9;8(8):e202403123. doi: 10.26508/lsa.202403123 (PMC12149561; doi:10.26508/lsa.202403123)
Supplement: Supplementary file 3 [file LSA-2024-03123_TableS3.docx]

**Table S3 - GOCC pathway analysis of cerulein-treated RGβKO acinar cells in comparison to treated WT acinar cells.** List of GOCC pathways obtained by gene set enrichment analysis after RNA sequencing from cerulein-treated RGβKO and WT sorted acinar cells. Given are the GOCC pathway name, p-value, q-value, enrichment score (ES), normalized enrichment score (NES), gene set size. Values are rounded to 4 decimal points and only the first 50 pathways are depicted sorted by increasing NES.

| **GOCC pathway** | **p-value** | **q-value** | **ES** | **NES** | **size** |
| --- | --- | --- | --- | --- | --- |
| GOCC_CILIARY_PLASM | 0.0000 | 0.0006 | -0.5179 | -2.2432 | 156 |
| GOCC_IMMUNOGLOBULIN_COMPLEX | 0.0000 | 0.0034 | -0.6131 | -2.1277 | 46 |
| GOCC_MICROBODY | 0.0000 | 0.0106 | -0.4710 | -2.0251 | 140 |
| GOCC_AXONEMAL_MICROTUBULE | 0.0000 | 0.0208 | -0.5689 | -1.9397 | 38 |
| GOCC_AXONEMAL_DYNEIN_COMPLEX | 0.0020 | 0.0254 | -0.6561 | -1.9077 | 50 |
| GOCC_MICROBODY_LUMEN | 0.0000 | 0.0277 | -0.5345 | -1.8923 | 65 |
| GOCC_MICROBODY_MEMBRANE | 0.0000 | 0.0378 | -0.4926 | -1.8495 | 232 |
| GOCC_MOTILE_CILIUM | 0.0000 | 0.0525 | -0.3914 | -1.7907 | 149 |
| GOCC_SPERM_MIDPIECE | 0.0022 | 0.0680 | -0.4986 | -1.7421 | 275 |
| GOCC_TERMINAL_BOUTON | 0.0022 | 0.0722 | -0.5037 | -1.7313 | 122 |
| GOCC_9PLUS2_MOTILE_CILIUM | 0.0000 | 0.0754 | -0.4038 | -1.7235 | 468 |
| GOCC_BRUSH_BORDER_MEMBRANE | 0.0060 | 0.1152 | -0.4547 | -1.6497 | 291 |
| GOCC_CYTOPLASMIC_REGION | 0.0000 | 0.1151 | -0.3565 | -1.6476 | 231 |
| GOCC_DENDRITE_MEMBRANE | 0.0122 | 0.1189 | -0.4878 | -1.6428 | 101 |
| GOCC_NEURON_PROJECTION_MEMBRANE | 0.0044 | 0.1225 | -0.4443 | -1.6315 | 410 |
| GOCC_NEURON_PROJECTION_TERMINUS | 0.0000 | 0.1242 | -0.3852 | -1.6251 | 174 |
| GOCC_A_BAND | 0.0195 | 0.1338 | -0.4999 | -1.6055 | 285 |
| GOCC_PROTON_TRANSPORTING_V_TYPE_ATPASE_COMPLEX | 0.0258 | 0.1444 | -0.5127 | -1.5840 | 395 |
| GOCC_9PLUS0_NON_MOTILE_CILIUM | 0.0023 | 0.1555 | -0.3808 | -1.5640 | 390 |
| GOCC_SYNAPTIC_CLEFT | 0.0295 | 0.1755 | -0.5345 | -1.5415 | 447 |
| GOCC_MITOCHONDRIAL_MATRIX | 0.0000 | 0.1852 | -0.3093 | -1.5281 | 384 |
| GOCC_NON_MOTILE_CILIUM | 0.0046 | 0.1896 | -0.3548 | -1.5181 | 92 |
| GOCC_M_BAND | 0.0377 | 0.1898 | -0.5270 | -1.5144 | 90 |
| GOCC_PHOTORECEPTOR_OUTER_SEGMENT | 0.0068 | 0.1942 | -0.3884 | -1.5084 | 250 |
| GOCC_COPI_COATED_VESICLE_MEMBRANE | 0.0608 | 0.1981 | -0.5302 | -1.5013 | 451 |
| GOCC_NEURONAL_DENSE_CORE_VESICLE | 0.0462 | 0.2121 | -0.4905 | -1.4820 | 171 |
| GOCC_CELL_BODY_MEMBRANE | 0.0473 | 0.2152 | -0.4690 | -1.4797 | 272 |
| GOCC_DENSE_CORE_GRANULE | 0.0323 | 0.2221 | -0.4378 | -1.4719 | 81 |
| GOCC_DYNEIN_AXONEMAL_PARTICLE | 0.0586 | 0.2234 | -0.5044 | -1.4686 | 77 |
| GOCC_CILIARY_TRANSITION_ZONE | 0.0281 | 0.2616 | -0.3725 | -1.4211 | 196 |
| GOCC_PROTON_TRANSPORTING_TWO_SECTOR_ATPASE_COMPLEX | 0.0467 | 0.2774 | -0.4010 | -1.4040 | 145 |
| GOCC_CILIARY_MEMBRANE | 0.0374 | 0.2815 | -0.3726 | -1.4002 | 25 |
| GOCC_POTASSIUM_CHANNEL_COMPLEX | 0.0338 | 0.2853 | -0.3577 | -1.3937 | 138 |
| GOCC_PHOTORECEPTOR_INNER_SEGMENT | 0.0466 | 0.2906 | -0.3691 | -1.3892 | 250 |
| GOCC_T_CELL_RECEPTOR_COMPLEX | 0.0781 | 0.2955 | -0.4230 | -1.3828 | 31 |
| GOCC_SMOOTH_ENDOPLASMIC_RETICULUM | 0.0870 | 0.3012 | -0.4305 | -1.3727 | 115 |
| GOCC_CHROMOSOME_CENTROMERIC_CORE_DOMAIN | 0.1022 | 0.3053 | -0.4947 | -1.3676 | 183 |
| GOCC_POSTSYNAPTIC_SPECIALIZATION_MEMBRANE | 0.0482 | 0.3168 | -0.3310 | -1.3556 | 92 |
| GOCC_SARCOPLASMIC_RETICULUM_MEMBRANE | 0.0819 | 0.3188 | -0.4004 | -1.3499 | 65 |
| GOCC_ENZYME_ACTIVATOR_COMPLEX | 0.1252 | 0.3246 | -0.4966 | -1.3423 | 191 |
| GOCC_AMPA_GLUTAMATE_RECEPTOR_COMPLEX | 0.1119 | 0.3489 | -0.4415 | -1.3167 | 441 |
| GOCC_GABA_ERGIC_SYNAPSE | 0.0649 | 0.3518 | -0.3400 | -1.3140 | 134 |
| GOCC_GOLGI_ASSOCIATED_VESICLE_MEMBRANE | 0.0872 | 0.3561 | -0.3633 | -1.3105 | 177 |
| GOCC_POSTSYNAPTIC_MEMBRANE | 0.0299 | 0.3769 | -0.2825 | -1.2920 | 88 |
| GOCC_ATPASE_DEPENDENT_TRANSMEMBRANE_TRANSPORT_COMPLEX | 0.1692 | 0.3786 | -0.4500 | -1.2903 | 27 |
| GOCC_POSTSYNAPTIC_SPECIALIZATION | 0.0244 | 0.3839 | -0.2696 | -1.2821 | 405 |
| GOCC_SARCOPLASMIC_RETICULUM | 0.0932 | 0.3887 | -0.3382 | -1.2770 | 62 |
| GOCC_NEURON_SPINE | 0.0363 | 0.3938 | -0.2892 | -1.2710 | 64 |
| GOCC_PHOTORECEPTOR_CONNECTING_CILIUM | 0.1226 | 0.3937 | -0.3709 | -1.2705 | 384 |
| GOCC_MANCHETTE | 0.1745 | 0.3993 | -0.4469 | -1.2667 | 97 |
