# Supplementary material for: RalGAP complexes control secretion and primary cilia in pancreatic disease
Source: Life Sci Alliance. 2025 Jun 9;8(8):e202403123. doi: 10.26508/lsa.202403123 (PMC12149561; doi:10.26508/lsa.202403123)
Supplement: Supplementary file 4 [file LSA-2024-03123_TableS4.docx]

**Table S4 - shRNA oligonucleotide sequences**

| **Oligonucleotide** | **Sequence** |
| --- | --- |
| shmRalBP1 #1 F | CCGG ccagagaatttgcttaccaaa CTC GAG tttggtaagcaaattctctgg TTTTTG |
| shmRalBP1 #1 R | AATTCAAAAA ccagagaatttgcttaccaaa CTC GAG tttggtaagcaaattctctgg |
| shmRalBP1 #2 F | CCGG gcacaggagatagctagt CTT CTC gagaagactagctatctcctgtgc TTTTTG |
| shmRalBP1 #2 R | AATTCAAAAA gcacaggagatagctagt CTT CTC gagaagactagctatctcctgtgc |
| shmRalBP1 #3 F | CCGG gccagcttgctgaagcagtat CTCGAG atactgcttcagcaagctggc TTTTTG |
| shmRalBP1 #3 R | AATTCAAAAA gccagcttgctgaagcagtat CTCGAG atactgcttcagcaagctggc |
| shmSec5 #2 F | CCGG ggtcggaaagacaaggcagat CTCGAG atctgccttgtctttccgacc TTTTTG |
| shmSec5 #2 R | AATTCAAAAA ggtcggaaagacaaggcagat CTCGAG atctgccttgtctttccgacc |
| shmSec5 #3 F | CCGG gaacgccctcaacgtacttca CTCGAG tgaagtacgttgagggcgttc TTTTTG |
| shmSec5 #3 R | AATTCAAAAA gaacgccctcaacgtacttca CTCGAG tgaagtacgttgagggcgttc |
| shmRalA/B #2 F | CCGG gagtttgtagaagactatga CTCGAG tcatagtcttctacaaactc TTTTTG |
| shmRalA/B #2 R | AATTCAAAAA gagtttgtagaagactatga CTCGAG tcatagtcttctacaaactc |
| shmRalA/B #4 F | CCGG gacaactacttccgaagcgga CTCGAG tccgcttcggaagtagttgtc TTTTTG |
| shmRalA/B #4 R | AATTCAAAAA gacaactacttccgaagcgga CTCGAG tccgcttcggaagtagttgtc |
| shmTBK1 #1 F | CCGG gaacgcagactagcttataat CTCGAG attataagctagtctgcgttc TTTTTG |
| shmTBK1 #1 R | AATTCAAAAA gaacgcagactagcttataat CTCGAG attataagctagtctgcgttc |
| shmTBK1 #3 F | CCGG gttggagtgacattctaccat CTCGAG atggtagaatgtcactccaac TTTTTG |
| shmTBK1 #3 R | AATTCAAAAA gttggagtgacattctaccat CTCGAG atggtagaatgtcactccaac |
| shmRGβ #4 F | CCGG aacctgggaagtcttactgtt CTCGAG aacagtaagacttcccaggtt TTTTTG |
| shmRGβ #4 F | AATTCAAAAA aacctgggaagtcttactgtt CTCGAG aacagtaagacttcccaggtt |
| shGFP F | CCGG gcaagctgaccctgaagttca CTC GAG tgaacttcagggtcagcttgc TTTTTG |
| shGFP R | AATT CAAAAA gcaagctgaccctgaagttca CTC GAG tgaacttcagggtcagcttgc |
