# Supplementary material for: RalGAP complexes control secretion and primary cilia in pancreatic disease
Source: Life Sci Alliance. 2025 Jun 9;8(8):e202403123. doi: 10.26508/lsa.202403123 (PMC12149561; doi:10.26508/lsa.202403123)
Supplement: Supplementary file 5 [file LSA-2024-03123_TableS5.docx]

**Table S5 - CRISPR/Cas9-related oligonucleotides and cloning primers**

| **Oligonucleotide** | **Sequence** |
| --- | --- |
| RGα1sg1_F | CACC G CAGCACGGAGAGACCCACGT |
| RGα1sg1_R | AAAC ACGTGGGTCTCTCCGTGCTG C |
| RGα1sg2_F | CACC G TGTGTGCTCCTTGTAAACAG |
| RGα1sg2_R | AAAC CTGTTTACAAGGAGCACACA C |
| mRGα1 screen 2F | AAGACTACGGGTTCCACTGG |
| mRGα1 screen 2R | TGCTGGAGATCTGGTGCTAG |
| mRGα1RTEx1_F3 | GACGTGAAGAAGTCCACCC |
| mRGα1RTEx1_R3 | GGTCAATAGATTCTGCATTCTCG |
| RGα2sg1_F | CACC G TCA GGC ACA GAG GTT ACC CG |
| RGα2sg1_R | AAAC CGGGTAACCTCTGTGCCTGA C |
| RGα2sg2_F | CACC G GCG GAC CAG AGG CAC CCG TG |
| RGα2sg2_R | AAAC CACGGGTGCCTCTGGTCCGC C |
| mRGα2 screen 4F | GAGTGAGAGATGGTAGTTCAACG |
| mRGα2 screen 4R | GGAGGTCACTGCGCAGACTCGAAGC |
| mRGα2RTEx1_F | GAAGGAGCCACGGAGATGT |
| mRGα2RTEx1_R | CATCCACGTTATCCAGCAGC |
| RGβsg1_F | CACC G AGA AGC AGT AGT GGT AGT GT |
| RGβsg1_R | AAAC ACACTACCACTACTGCTTCT C |
| RGβsg2_F | CACC G GCT GCT AAC TCC AGT TGC AG |
| RGβsg2_R | AAAC CTGCAACTGGAGTTAGCAGC C |
| RGβsg3_F | CACC G AGA GAG TGT TGG GCG AGA GG |
| RGβsg3_R | AAAC CCTCTCGCCCAACACTCTCT C |
| mRGβ screen 2F | TGAAAGGGAAATGTCGGAAA |
| mRGβ screen 2R | TGAGTTCCTGCCTTGGTTTT |
| mRGβRTEx2_F | CAGTGGCTGGTAGTGAGAGT |
| mRGβRTEx2_R | GCAACACCAAAGCCATAATCC |
| RGβ_MluI_F | ATAACGCGTTACTCTGAGTGGAGGTCACTGC |
| RGβ_NotI_R | ATATAGCGGCCGCCTAAGAACTGCAGTTCTTGAGTCC |
| RGα2_MluI_F | ATATAACGCGTTTCAGCAGACGATCTCACGG |
| RGα2_ NotI_R | ATAGCGGCCGCTCAGTCTGTGCCGCTCAG |
| KRas_MluI_F | ATAACGCGTACGGAATATAAGCTTGTGG |
| KRas_NotI_R | ATATAGCGGCCGCTCACATAATTACACACTTTGTC |
