# Supplementary material for: RalGAP complexes control secretion and primary cilia in pancreatic disease
Source: Life Sci Alliance. 2025 Jun 9;8(8):e202403123. doi: 10.26508/lsa.202403123 (PMC12149561; doi:10.26508/lsa.202403123)
Supplement: Supplementary file 6 [file LSA-2024-03123_TableS6.docx]

**Table S6 - qPCR primer sequences**

| **qPCR Primer** | **Sequence** |
| --- | --- |
| mRalBP1_F1 | GCAGCAGCCCTAGTGAACA |
| mRalBP1_R1 | CCGGTACAATCCAGGGAACTT |
| mTBK1_F1 | ACTGGTGATCTCTATGCTGTCA |
| mTBK1_R1 | TTCTGGAAGTCCATACGCATTG |
| mSec5_F1 | GGGAAAACGGAGGTGCAAGT |
| mSec5_R1 | CTGGTCATGTAAGGTTGATGGAG |
| mRalA_F2 | ATGTACGACGAGTTTGTAGAGGA |
| mRalA_R2 | CCCGCTGTATCTAAGATGTCGAT |
| mRalB_F2 | GCTCCCTGGTACTTCACAAGG |
| mRalB_R2 | ATGGCCGCATAGTCCTCCT |
| mMist1_F2 | TGACCGCCACCATACTTACTA |
| mMist1_R2 | GCTGGTATAATTTAGGGCCTGG |
| mRGβ_F4 | CATCATGGCTCGAGAAACCT |
| mRGβ_R4 | GATTCTCAGCAATGCCACCT |
| mSox9_F | CAAGACTCTGGGCAAGCTCTG |
| mSox9_R | TCCGCTTGTCCGTTCTTCAC |
| mPtf1a_F | TGC GCT TGG CCA TAG GCT ACA TTA |
| mPtf1a_R | AGA TGA TAA CCT TCT GGG CCT GGT |
| mGAPDH_F | GGTGAAGGTCGGTGTGAACG |
| mGAPDH_R | CTCGCTCCTGGAAGATGGTG |
| mCK19_F2 | GGA CCC TCC CGA GAT TAC AAC CA |
| mCK19_R2 | GCC AGC TCC TCC TTC AGG CTC T |
| mAmylase_F2 | TGG CGT CAA ATC AGG AAC ATG |
| mAmylase_R2 | GGC TGA CAA AGC CCA GTC ATC A |
| mInsulin_F1 | AGACCTTGGCGTTGGAGGTGGCCCG |
| mInsulin_R1 | GCAGAGGGGTGGGGCGGGTCGAC |
